# Single-cell transcriptome identifies molecular subtype of autism spectrum disorder impacted by de novo loss-of-function variants regulating glial cells

**DOI:** 10.1186/s40246-021-00368-7

**Published:** 2021-11-21

**Authors:** Nasna Nassir, Asma Bankapur, Bisan Samara, Abdulrahman Ali, Awab Ahmed, Ibrahim M. Inuwa, Mehdi Zarrei, Seyed Ali Safizadeh Shabestari, Ammar AlBanna, Jennifer L. Howe, Bakhrom K. Berdiev, Stephen W. Scherer, Marc Woodbury-Smith, Mohammed Uddin

**Affiliations:** 1 College of Medicine, Mohammed Bin Rashid University of Medicine and Health Sciences, Dubai, UAE; 2 Biomedical Engineering Department, McGill University, Montréal, QC Canada; 3 The Centre for Applied Genomics (TCAG), The Hospital for Sick Children, Toronto, ON Canada; 4 Genetics and Genome Biology, The Hospital for Sick Children, Toronto, ON Canada; 5 Mohammed Bin Rashid University of Medicine and Health Sciences, Dubai, UAE; 6 The Mental Health Center of Excellence, Al Jalila Children’s Speciality Hospital, Dubai, UAE; 7 Molecular Genetics, University of Toronto, Toronto, ON Canada; 8 Biosciences Institute, Newcastle University, Newcastle upon Tyne, UK; 9 Cellular Intelligence (Ci) Lab, GenomeArc Inc., Toronto, ON Canada

**Keywords:** Single-cell transcriptomics, Autism spectrum disorder, De novo LOF variant, Glial cell type, Brain tissue

## Abstract

**Background:**

In recent years, several hundred autism spectrum disorder (ASD) implicated genes have been discovered impacting a wide range of molecular pathways. However, the molecular underpinning of ASD, particularly from the point of view of ‘brain to behaviour’ pathogenic mechanisms, remains largely unknown.

**Methods:**

We undertook a study to investigate patterns of spatiotemporal and cell type expression of ASD-implicated genes by integrating large-scale brain single-cell transcriptomes (> million cells) and de novo loss-of-function (LOF) ASD variants (impacting 852 genes from 40,122 cases).

**Results:**

We identified multiple single-cell clusters from three distinct developmental human brain regions (anterior cingulate cortex, middle temporal gyrus and primary visual cortex) that evidenced high evolutionary constraint through enrichment for brain critical exons and high pLI genes. These clusters also showed significant enrichment with ASD loss-of-function variant genes (*p* < 5.23 × 10^–11^) that are transcriptionally highly active in prenatal brain regions (visual cortex and dorsolateral prefrontal cortex). Mapping ASD de novo LOF variant genes into large-scale human and mouse brain single-cell transcriptome analysis demonstrate enrichment of such genes into neuronal subtypes and are also enriched for subtype of non-neuronal glial cell types (astrocyte, *p* < 6.40 × 10^–11^, oligodendrocyte, *p* < 1.31 × 10^–09^).

**Conclusion:**

Among the ASD genes enriched with pathogenic de novo LOF variants (i.e. *KANK1*, *PLXNB1*), a subgroup has restricted transcriptional regulation in non-neuronal cell types that are evolutionarily conserved. This association strongly suggests the involvement of subtype of non-neuronal glial cells in the pathogenesis of ASD and the need to explore other biological pathways for this disorder.

**Supplementary Information:**

The online version contains supplementary material available at 10.1186/s40246-021-00368-7.

## Background

Autism spectrum disorder (ASD) is a neurodevelopmental disorder of childhood onset whose aetiology is principally genetic [1–3] and whose phenotype is highly heterogeneous [4, 5]. Even though hundreds of ASD-implicated genes have been reported [6], no single gene accounts for > 1% of cases [7]. Moreover, the phenotypic heterogeneity has confounded gene discovery and an understanding of the molecular pathways from ‘brain to behaviour’. Recent advances in single-cell technologies have enabled the investigation of this molecular heterogeneity at the single-cell level. Scrutinising molecular subtypes of ASD at the single-cell level will facilitate a greater understanding of ASD’s brain mechanisms, and their translation into accurate early diagnosis, better treatment outcomes and ultimately a precision medicine approach to ASD [8–10].

Brain development during the gestational stage introduces several progenitor brain cells that are responsible for forming an orderly cellular and mechanistically heterogeneous structure. Understanding how different brain cell types communicate with each other, and with sensory input and functional output, as the basis of cognition and perception is a central challenge in neuroscience. Although many mutations in ASD-implicated genes are reported, and an understanding of their association with brain pathways is evolving, a true sense of how cellular heterogeneity maps onto ASD molecular subtypes is still unknown.

Multiple independent studies have used developing human brain transcriptomic data to map ASD candidate genes onto spatiotemporal brain regions and molecular pathways [9, 11–16], but these studies are mostly limited to transcriptomics derived from bulk tissue or involve only limited analysis on single-cell transcriptomics [6]. In neurodevelopmental disorders, evidence is emerging from single-cell data that genes with clinically pathogenic mutations functionally impact a subset of primary brain cells, with the most consistent evidence converging on the neuron and its subtypes rather than non-neuronal cells such as astrocytes and oligodendrocytes [17–19]. This demonstrates the ability of single-cell OMICs to identify the role of particular cell types in the brain in the functional mechanisms of human cognition.

The distinct pattern of gene expression at single-cell resolution has led to the identification [20] of numerous known and unknown cell types in the human brain. Through the integrated analysis of mutational data and single-cell transcriptomics, it is possible to map those brain cell types that confer a significant risk of disease pathophysiology. In this study, we aim to identify the role of neuronal and non-neuronal cell types in ASD pathogenesis by investigating patterns of cell-type expression of ASD implicated genes by leveraging large-scale mutation and brain single-cell transcriptome data. Our findings suggest that, among those with ASD, a subgroup harbour loss-of-function mutation in genes that are evolutionarily constrained and regulate non-neuronal cell types in the brain.

## Results

### Curation of de novo missense and loss-of-function variants associated with ASD

We performed a systematic literature search to identify variants relevant to ASD. The ASD variant data were compiled from 26 studies (Additional file 2: Table S1) where ASD is the main phenotype (Additional file 1: Fig. S1). We included articles that used data from whole-exome, whole-genome and targeted sequencing (Additional file 1: Fig S1) cohorts (excluding case reports). Among the variants, 92.4% (156,688 out of 169,580) were reported to be de novo and 0.863% (1463 out of 169,580) were rare inherited. Variants in the dataset were classified based on their location in the genome. More than 90% of variants overlap noncoding DNA sequences reported in autism whole genome sequencing projects (e.g. MSSNG, ASC), including intergenic regions, introns and untranslated regions. Focusing on variants with direct impact on protein structure, *de nov*o exonic and splicing variants were found to make up 6.23% (10,565 out of 169,580) of our curated data. Among the de novo variants, 62.01% (6,651 out of 10,565) were classified as missense (Additional file 1: Fig. S2, Additional file 2: Table S2) and 10.29% (1087 out of 10,565) were loss of function (LOF) (splicing (203), nonsense (470), frameshift (414)). We mostly focused on de novo LOF variants (impacting 852 unique genes) (Additional file 2: Table S2) for our downstream analysis due to the consistent association of LOF mutations with clinical ASD [21, 22]. Our pathway enrichment analysis of all the de novo ASD LOF variant genes identified multiple pathways including neuronal regulation and differentiation (p < 5.29 × 10^–11^), cytoskeletal and helicase activity (p < 1.07 × 10^–07^), brain development (p < 1.76 × 10^–07^), synapse and neurotransmission (p < 4.07 × 10^–05^) (Additional file 1: Figs. S3, S4 and Additional file 2: Table S3).

### Construction of single-cell clusters and identification of genes that are differentially expressed across clusters

**Fig. 1:**
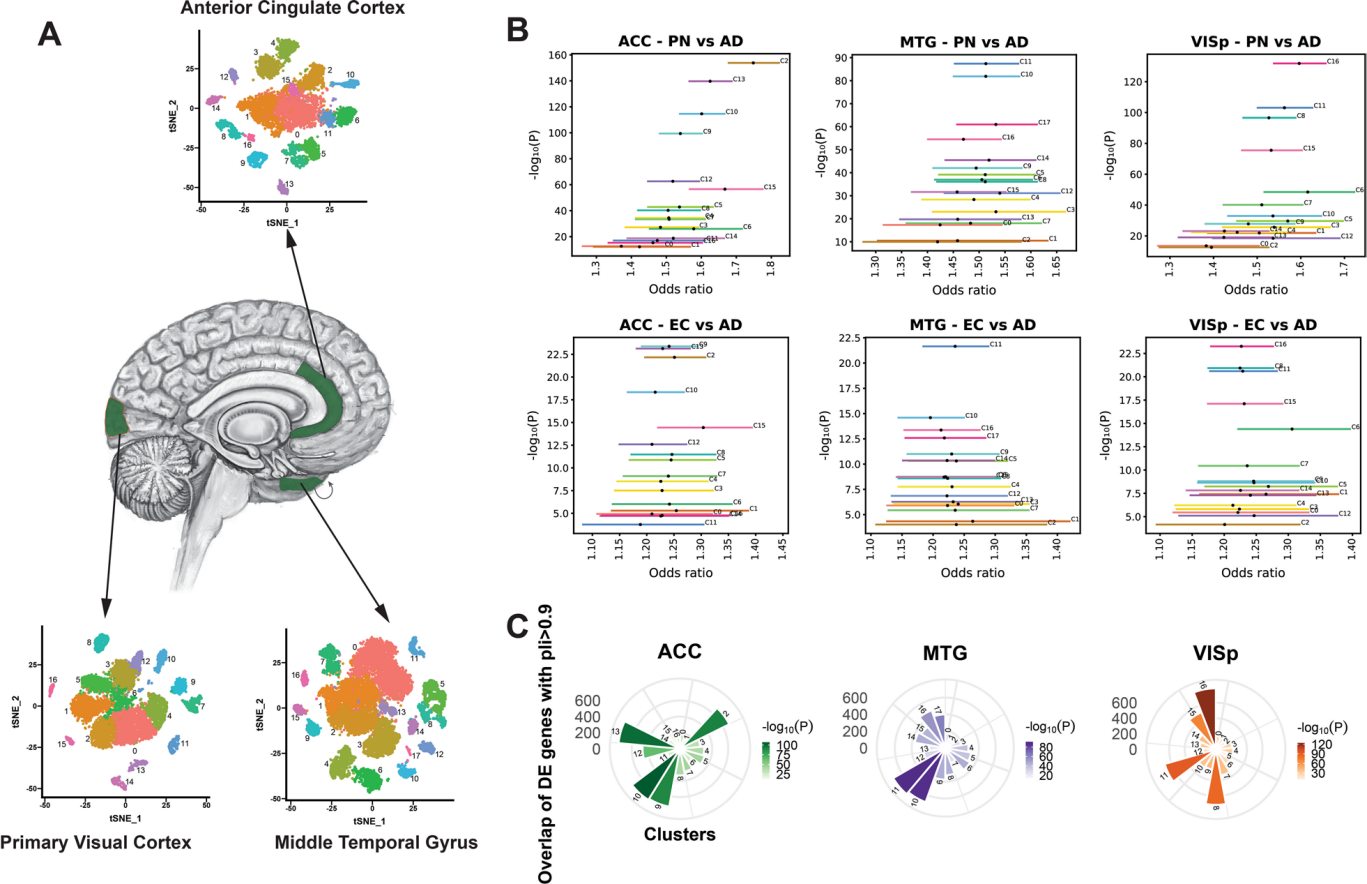
Constrained cluster analysis. **A** Clustering of single-cell RNA seq data from three distinct post-mortem neurotypical human brain regions (coloured green)—anterior cingulate cortex (ACC), middle temporal gyrus (MTG) and primary visual cortex (VISp). Clustering of single-cell transcriptome data is shown for each region defined by the tSNE plot (tSNE1 x-axis and tSNE2 y-axis), and each colour represents a unique cluster. **B** Enrichment of critical exon genes for each cluster is shown. Enrichment was conducted between differentially expressed genes of a cluster (with unique colour horizontal line) and their overlap with pre-computed critical exon matrix from prenatal (PN), early childhood (EC) and adult (AD) brain RNA seq data. Y-axis shows the significance (-log(p) and x-axis shows odds ratio (OR) of critical exon gene enrichment for each cluster and compared between the developmental stages. **C** Enrichment of high pLI (> 0.90) genes across all clusters shown in radial plots. Enrichment of each brain region shown in unique colours and the width and height of the graph represents OR and the colour intensity represents –log(p) of the enrichment value

We have used single-cell RNAseq data from 3 brain regions ACC (Anterior cingulate cortex), MTG (middle temporal gyrus) and VISP (primary visual cortex) derived from 8 neurotypical human tissue Allen Brain Atlas donors comprising 32,209 nuclei (ACC-7,283; MTG-15,928; VISP-8,998)[20]. RNAseq data were extensively processed and clustered (Additional file 1: Fig. S5) (detailed in Methods section) using Seurat v.3 [23], resulting in 17, 17 and 18 distinct cell clusters for ACC, MTG and VISP, respectively (Fig. 1A). The clusters are of varying sizes ranging from 100 to 3364 cells (Additional file 1: Fig. S6). To identify differentially expressed genes (DEGs) across clusters in all three brain regions, we have applied four statistical tests (Wilcox [24], t test [25], Bimod [26], MAST [27]) and a stringent cutoff of pval_adj_ < 0.001. Genes that were significant across all four tests were then used for further downstream analysis (Additional file 3: Table S4). This conservative analysis yielded differentially expressed genes for each cluster ranging from 349 to 2671 genes per cluster (Additional file 3: Table S4).

### Identification of cell clusters that are enriched for constraint genes

We next aimed to prioritise clusters for downstream analysis by identifying those enriched for highly constrained genes intolerant of mutations. To identify single-cell clusters that are regulated by constraint genes, we used two different approaches: i) brain critical exons and ii) pLI (probability of being LoF intolerant). Brain critical exons identify genes with exons that are both highly expressed and show constraint against mutation accumulation, whereas the pLI score characterises genes in terms of their tolerance to LOF mutation. Brain critical exons are reported to be highly constrained in human brain [28]. We have constructed a brain critical exon database using the gnomAD mutation database and RNA-seq data from 196 developing human brains [29] from three developmental stages (prenatal, early childhood and adulthood) following the method described in our previous work [28]. Our enrichment analysis identified that differentially regulated cluster genes are significantly enriched with brain critical genes in clusters ACC (2, 13, 10, 9), MTG (11, 10, 17, 16), VISP (16, 11, 8, 15). Brain critical exons are predominantly enriched in differentially expressed cluster genes that are highly expressed in prenatal stages compared to early childhood and adulthood stage (Fig. 1B, Additional file 2: Table S5). The most significant clusters are ACC-cluster 2 (*p* < 1.47 × 10^–154^, OR = 1.74), MTG-cluster 11 (*p* < 3.99 × 10^–88^, OR = 1.51) and VISP-cluster 16 (*p* < 1.43 × 10^–132^, OR = 1.59). A gene-based score, pLI [30] was applied with a cutoff of pLI ≥ 0.9 to identify single -cell clusters that are highly intolerant of LOF mutations. The clusters with most significant (see Additional file 2: Table S6) intolerant genes are ACC (10, 13, 9, 2), MTG (11, 10, 16, 17), VISP (16, 11, 8, 15) (Fig. 1C). pLI is highly effective in quantitating haplo-insufficient genes [31] and our analysis showed that ACC-cluster 10 (*p* < 8.55 × 10^–106^), MTG-cluster 11 (*p* < 9.05 × 10^–91^), and VISP-cluster 16 (*p* < 4.04 × 10^–122^) were those clusters most enriched for genes that are LOF intolerant and haplo-insufficient. In addition, we conducted replication-timing analysis as it has been reported that somatic mutations discovered in cancers [32, 33] and ASD gene mutations [34] are associated with late DNA replication. We found that the cluster genes are constrained in late replication timing compared to early replication (Additional file 1: Fig. S7, Additional file 4: Table S7).

### Genes harbouring ASD de novo LOF and missense variants are enriched in constrained clusters

**Fig. 2:**
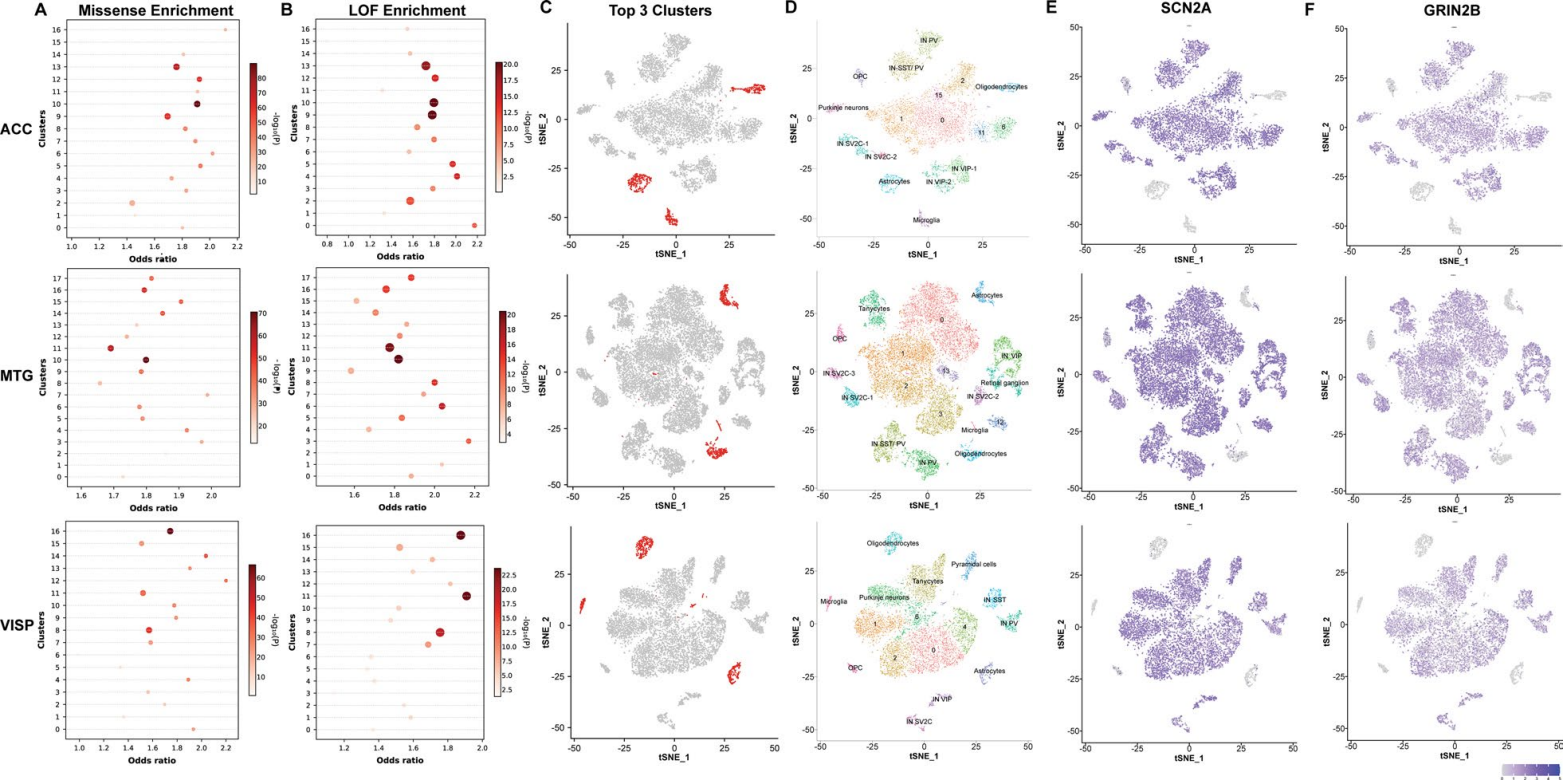
Enrichment of ASD missense and LOF variant genes across non-neuronal clusters of 3 brain regions—ACC (anterior cingulate cortex), MTG (middle temporal gyrus) and VISp (primary visual cortex). **A** Enrichment of ASD missense variants across clusters. The y axis here represents clusters, x axis odds ratio, the size of the circle by overlap gene size and the gradient represent p-value. Overlap size varies from 60 to 720, **B** Enrichment of ASD LOF variants across clusters. **C** LOF enriched clusters that are constrained are highlighted in red. **D** Scatterplot visualisation of cells after principal-component analysis and t-distributed stochastic neighbour embedding (tSNE), coloured by Seurat clustering and annotated by major cell types (neuron, oligodendrocytes, astrocytes, microglia, OPC) according to expression of known marker genes. We were unable to assign cell type identity for clusters 0, 1, 2, 6, 11, 15 (ACC), 0, 1, 2, 3, 12, 13 (MTG) and 0, 1, 2, 4, 6 (VISp) with the available set of known cell type markers. **E** Feature plot of neuronal bias gene, *SCN2A* across ACC, MTG and VISp regions. **F** Feature plot of neuronal bias gene, *GRIN2B* across ACC, MTG and VISp regions

We next conducted enrichment analysis between the single-cell clusters and 6651 de novo missense variants (impacting 4017 genes) and 1087 de novo LOF variants (impacting 852 genes) derived from our curation of the ASD mutational landscape as described above (Additional file 4: Table S8). Using the GeneOverlap package in R, we observed that ACC (10, 9,13), MTG (10, 11, 6) and VISP (16, 11, 8) clusters were most significantly enriched (Fig. 2A-C) for genes harbouring ASD LOF and missense variants (*p* < : ACC (1.55 × 10^–09^, 1.89 × 10^–09^, 1.16 × 10^–08^), MTG (1.31 × 10^–09^, 3.11 × 10^–09^, 6.74 × 10^–08^), VISP (5.23 × 10^–11^, 6.40 × 10^–11^, 6.04 × 10^–09^)). Interestingly, the de novo ASD LOF variant enriched clusters were also those clusters that we have found to be significantly constrained (based on enrichment of high pLI genes and critical exons). In addition, we have conducted enrichment analysis on three additional sets of genes that harbour i) multiple de novo LOF ii) multiple de novo missense and iii) multiple de novo LOF or missense variants in ASD. We observed the initial LOF enriched clusters ACC (10, 9, 13), MTG (10, 11, 6), VISP (16, 11, 8) are also highly enriched (see Additional file 1: Fig. S8, Additional file 4: Table S9 for enrichment) for these three additional sets of genes. These clusters are chosen for further downstream analysis.

Previous studies implicated that fragile X mental retardation protein (FMRP) genes are found to harbour clinically relevant de novo variants in ASD, and the phenotypic overlaps between fragile X and ASD are also well documented [35]. The phenotypic and genotypic overlap is also observed with epilepsy and intellectual disability. We conducted enrichment analysis of FMRP protein targets, epilepsy and ID LOF gene lists (Additional file 3: Table S10) across clusters and observed enrichment in the same highly constrained clusters found to be enriched for genes harbouring de novo ASD LOF/missense variants, hereafter referred to as ‘ASD LOF enriched clusters’ (Additional file 4: Table S11). Of note, the housekeeping genes (negative control) (Additional file 3: Table S10) were not enriched in any of the clusters across brain regions. Further, the enrichment analysis of pathway genes involved in autism spectrum disorder (Additional file 3 and Additional file 4: Table S10, S12) was also carried out.

### Subtypes of glial cells are enriched with ASD de novo LOF mutated genes

Classifying single-cell transcriptomic clusters in terms of cell identity is complex; consequently, to assign cluster identity we have used two independent approaches. First, we used an unbiased approach to identify cell types enriched in each cluster based on their molecular signatures employed by a set of known marker genes (Fig. 2D). For our analysis, we used 865 known gene markers curated from literature [36–38] (Additional file 3: Table S13). Initial analysis on two selected well-studied ASD genes (*SCN2A, GRIN2B*) known to harbour pathogenic de novo LOF variants that are involved in critical neuronal inhibitory and excitatory functions confirmed their enrichment in various neuronal subtypes (Fig. 2E, F).

**Fig. 3:**
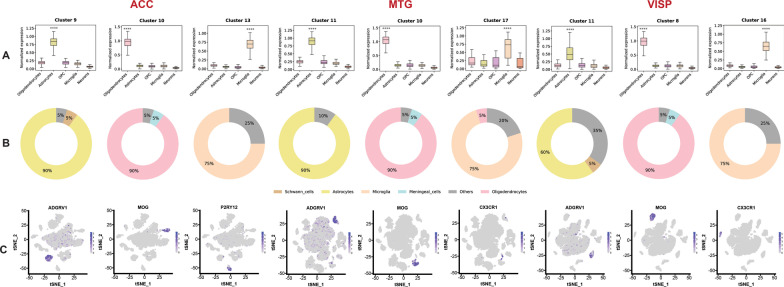
Expression of known marker genes in clusters of 3 brain regions, ACC (anterior cingulate cortex), MTG (middle temporal gyrus) and VISp (primary visual cortex). **A** Boxplot representing the average expression of cluster genes related to specific cell types (known marker genes); box plots showing median, interquartile range (IQR) with whiskers adding IQR to the 1st and 3rd quartile. Y-axis represents normalised gene expression, x-axis represents known marker genes of brain cell types, oligodendrocytes, astrocytes, OPC, microglia and neurons. **B** Donut plot displaying the composition of top 20 DE genes in comparison to known marker gene list (yellow-astrocytes, orange-microglia, pink-oligodendrocytes, grey-others). **C** Feature plot to visualise the expression of known marker gene related to specific cell types on tSNE plot. Expression of astrocytes (ADGRV1), oligodendrocytes (MOG) and microglia (P2RY12/ CX3CR1) across different clusters are also shown

We next conducted enrichment analysis for the entire de novo LOF mutated genes. In this way, we identified that the top ASD de novo LOF enriched clusters are characterised by a significantly higher expression of non-neuronal marker genes for astrocytes, oligodendrocytes, microglia and oligodendrocyte progenitor cells (OPC) compared to neurons. We observed that top ASD de novo LOF cluster genes were over-represented among non-neuronal cell markers (Fig. 3A, Additional file 1: Fig. S9). Also, LOF genes had higher composition of known non-neuronal marker genes compared to neuronal marker genes (Additional file 3: Table S14).

Secondly, we further looked at the composition of the top 20 DE genes, which represent the primary regulatory molecular machinery that defines each cluster. Our analysis observed that the top 3 significant clusters for each brain region included in our study were composed of non-neuronal cells, i.e. astrocytes, oligodendrocytes and microglia marker genes (Fig. 3B, Additional file 1: Fig. S10). We further looked for the evidence from expression of well-characterised non-neuronal marker genes for primary brain cell types. Our analysis found that multiple non-neuronal marker genes have restrictively high expression in our selected top three ASD de novo LOF enriched clusters (Fig. 3C). Based on these three lines of evidence, we observed that a subset of ASD LOF enriched constrained clusters were non-neuronal, i.e. comprised astrocytes, oligodendrocytes, and microglia. As such, this indicates that a subgroup of ASD-implicated genes is preferentially involved in non-neuronal brain processes, thereby earmarking these non-neuronal elements in the aetiology of ASD among those individuals harbouring such variants. Hierarchical clustering of cell type depicting lineage relationship based on DEGs was performed using Slingshot [39] (Additional file 1: Fig. S11). Venn diagrams depicting the overlap between genes among all clusters across 3 brain regions were also plotted (Additional file 1: Fig. S12).

### Spatiotemporal characterisation of ASD LOF enriched clusters

**Fig. 4:**
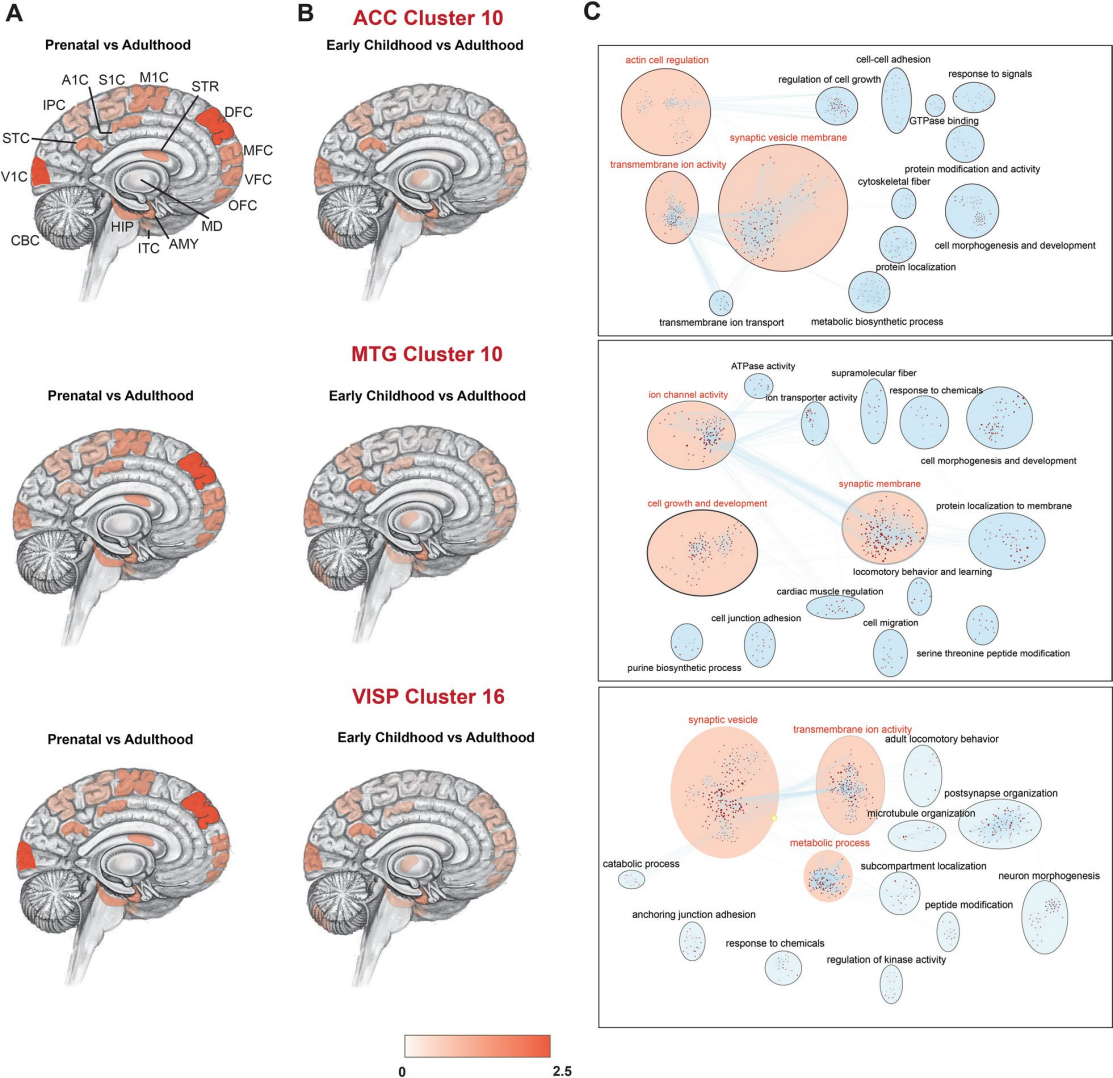
Spatiotemporal association analysis of critical exons in cluster genes. **A** Spatiotemporal heat maps (with the locations of brain tissue samples depicted on the medial surface) include information on 16 brain regions that were outlined in 3 developmental human brain stages (prenatal, early childhood and adult). Enrichment of critical exons show highest expression in prenatal stage and DFC/V1C region. For each brain region, (AMY, amygdaloid complex; CBC, cerebellar cortex; V1C, primary visual cortex; STC, posterior (caudal) superior temporal cortex; IPC, posterior inferior parietal cortex; A1C, primary auditory cortex; S1C, primary somatosensory cortex; M1C, primary motor cortex; STR, striatum; DFC, dorsolateral prefrontal cortex; MFC, medial prefrontal cortex; VFC, ventrolateral prefrontal cortex; OFC, orbital frontal cortex; MD, mediodorsal nucleus of thalamus; ITC, inferolateral temporal cortex; HIP, hippocampus), the colour gradient reflects the association Odds ratio between expression levels and the burden of rare missense variants. **B** Pathway network analysis in ASD LOF enriched clusters drawn using Cytoscape. Colour gradient and size of nodes are represented by pvalue and Odds ratio, respectively. Top 3 pathway clusters are highlighted in red

For further characterising these non-neuronal ASD LOF enriched clusters, we examined the spatiotemporal dynamics across three developmental periods (prenatal, early childhood and adulthood) and in 16 brain regions. We observed the strongest expression in the prenatal developmental stage (Fig. 4A, B, Additional file 5: Table 15). Further, critical genes in the ASD LOF enriched clusters showed significant expression in DFC (*p* < 2.88 × 10^–277^) and V1C (*p* < 4.64 × 10^–214^) brain regions, both known to be critical for cognitive function. The association of DFC and V1C is consistent in all top ASD LOF enriched clusters derived from three brain regions. In addition, network analysis identified that synaptic vesicle, ion activity and cell regulation are significantly enriched and common among all three ASD LOF clusters derived from the three brain regions (Fig. 4C, Additional file 1: Fig. S13, Additional file 6: Table S16).

### Replication of conserved expression pattern of ASD LOF genes in non-neuronal cell types

**Fig. 5:**
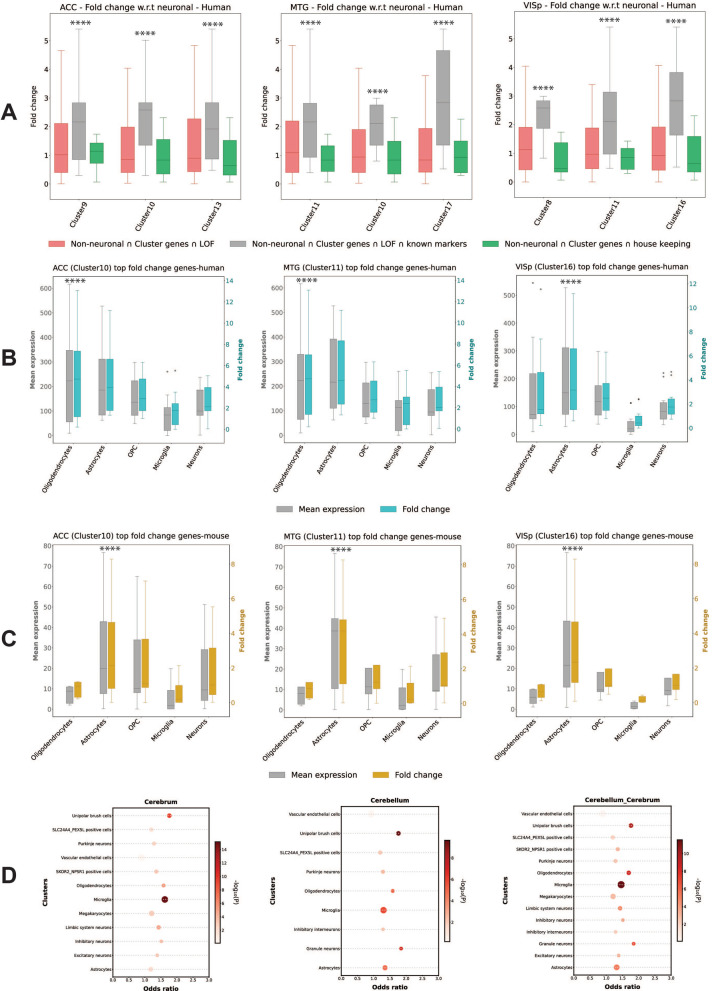
Replication of association between cluster genes and cell type in other validation datasets. **A** Fold change (y axis) of top cluster genes intersected with LOF genes, non-neuronal genes, known marker genes and housekeeping genes across ACC, MTG and VISp brain regions. **B** Cluster-cell type associations (plotted by mean expression (grey) for the 10 most fold change (blue) genes on y axis in 3 significant clusters across oligodendrocytes, astrocytes, OPC, microglia, neurons among ACC, MTG and VISp brain regions. **C** Mean expression (grey) of 10 most fold change (yellow) genes on y axis in 3 significant clusters across oligodendrocytes, astrocytes, OPC, microglia, neurons among ACC, MTG and VISp brain regions. **D** Enrichment of ASD LOF variant genes across cell types of cerebrum and cerebellum regions of fetal brain. The y axis here represents cell types, x axis odds ratio, the size of the circle by overlap gene size and the gradient represent *p* value

For replicating our findings, we examined additional human and mouse brain single-cell transcriptomes. Unlike our discovery analysis using single-cell transcriptomics data, this human brain single-cell dataset used pre-sorted (based on markers) brain cells [40]. We found that the top ASD de novo LOF enriched clusters were characterised by high expression in non-neuronal cells (astrocytes: *p* < 4.01 × 10^–03^; oligodendrocytes: *p* < 1.22 × 10^–04^) (Fig. 5A, Additional file 1: Fig. S14). The data also showed neuronal expression for other de novo ASD LOF enriched clusters consistent with our previous observation. We also analysed the expression of top ten-fold change genes (Additional file 3: Table S4) across clusters (Fig. 5B, Additional file 1: Fig. S15) and observed higher expression in non-neuronal cells compared to neurons. The mean expression of ASD LOF genes was also plotted, which shows higher expression within non-neuronal marker genes compared to neuronal markers (Additional file 1: Fig. S16).

To assess the robustness of our results and to examine evolutionary conservation across other mammals, we used a dataset that identified broad categories of cell types from mouse brain regions [41]. Our analysis confirmed that genes from the ASD de novo LOF enriched clusters show significant expressed in astrocytes (Fig. 5C, Additional file 1: Fig. S17). A second independent mouse brain single-cell transcriptome dataset [42] also showed a similar pattern (Additional file 1: Fig. S18).

We further used cell type-specific gene expression data from another human brain [43] dataset to quantify mean expression of the top ten DEGs from each cluster across fetal and adult astrocytes. We consistently observed that top ten fold change genes from our previous analysis were more active in adult astrocytes, postnatally after the neurons mature (Additional file 1: Fig. S19). Enrichment of ASD LOF genes across cell types of cerebrum and cerebellum in fetal brain [44] shows expression in non-neuronal cells (microglia, oligodendrocytes) and unipolar brush cells (Fig. 5D). We thus observed distinct cell type association between ASD risk genes and glial-specific gene expression throughout development. These results not only support the genetic evidence indicating that non-neuronal cells may play a role in ASD, but also indicate involvement of non-neuronal cell type-related disease aetiology of ASD.

**Fig. 6:**
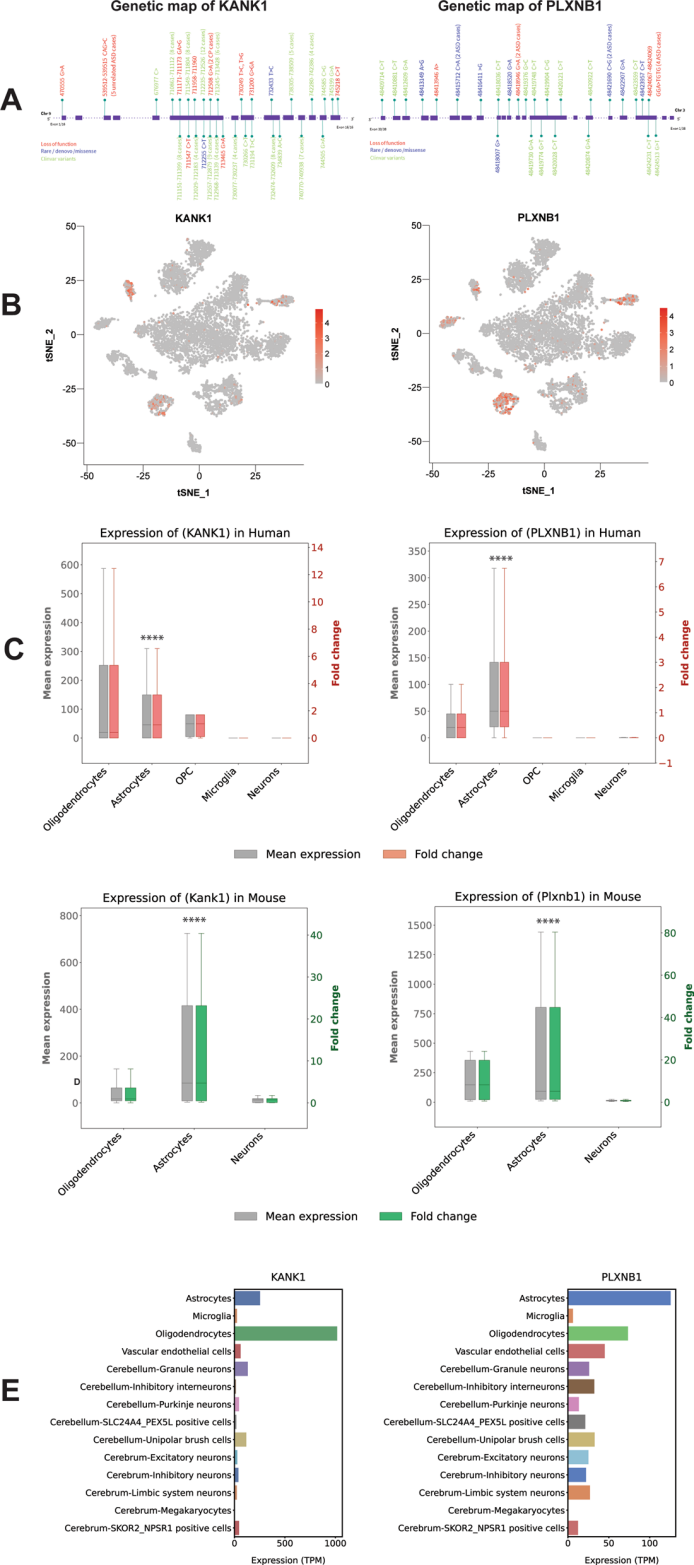
Genes impacted with clinically relevant mutations with restricted non-neuronal brain cell expression. **A** Loss-of-function (LOF) (red text), de novo/rare missense variants (blue text), ClinVar variants (green text) and their genomic location within the exons (blue squares) of non-neuronal genes (*KANK1* and *PLXNB1*). Detailed variant information is provided in Additional file 3: Tables 17, 18. **B** Feature plot showing restrictive expression (scale red to white) of *KANK1* and *PLXNB1* across single-cell clusters from human brain. **C** Mean expression (grey) and fold change (red) of *KANK1* and *PLXNB1*- human primary brain cell types (oligodendrocytes, astrocytes, oligodendrocyte progenitor cell (OPC), microglia, neurons). **D** Mean expression (green) and fold change (red) of *KANK1* and *PLXNB1*- mouse primary brain cell types (oligodendrocytes, astrocytes, neurons). **E** Expression (TPM) of *KANK1* and *PLXNB1* across cell types of cerebrum and cerebellum regions of fetal brain

Our results suggest a subset of ASD genes have significant non-neuronal bias in expression. By way of example, both *KANK1* and *PLXNB1* showed very restricted expression in non-neuronal cell types in all three brain regions. Both *KANK1* and *PLXNB1* are shown to harbour multiple LOF variants in our curated ASD mutation data set. We have identified additional 2 de novo LOF variants, 14 LOFs with unknown inheritance and 4 de novo missense from 11 ASD cases for *KANK1* from other autism genetic research laboratory data (including MSSNG database) (Fig. 6A, Additional file 3: Table S17). Similarly, for *PLXNB1*, we have identified 5 de novo LOF variants, 2 LOFs with unknown inheritance and 10 de novo missense in 8 ASD cases (Fig. 6A, Additional file 3: Table S18). There is significant enrichment of frameshift mutations in *PLXNB1* (p = 0.0143) and splice site mutations in *KANK1* (p = 0.0012, Additional file 3: Table S19) compared to control (gnomAD). In addition, we looked into other databases (Clinvar entries, DECIPHER) to accumulate more variants from cases (Fig. 6A, Additional file 3: Tables S17, S18). We found that 24 of 115 *KANK1* and 5 of 35 *PLXNB1* variants are damaging mutations (based on rare variant ACMG guidelines). Further analysis on CNV shows enrichment of de novo CNVs for both *PLXNB1* and *KANK1* gene within ASD and neurodevelopmental disorder cases (Additional file 1: Fig. S20, Additional file 3: Table S17, S18). Both *KANK1* and *PLXNB1* have shown restricted high expression in astrocytes in both of our human single-cell data (Fig. 6B, Additional file 1: Fig. S21). This pattern of restricted astrocyte expression for both genes was found to be significant compared to neuron expression in human brain [40] and multiple mouse brain single-cell transcriptome data [41, 42] (Fig. 6C and Fig. 6D, Additional file 1: Figs. S22, S23, S24). Further, these critical genes for ASD were highly expressed in fetal astrocytes compared to adult astrocytes (Additional file 1: Fig. S25). Expression of critical genes, *KANK1* and *PLXNB1* across cell types of cerebrum and cerebellum in fetal brain [44] shows expression in non-neuronal cells (Fig. 6E, Additional file 1: Fig. S26). *KANK1* and *PLXNB1* were expressed in non-neuronal cell types (Additional file 1: Fig. S27) in other single-cell datasets too [45, 46].

## Discussion

In this study, we leveraged large-scale transcriptomic data, including human and mouse brain single-cell transcriptomes, and systematically integrated ASD mutation data from published ASD sequencing projects to examine the association between these mutations and cell type. Although much of the literature has emphasised the role of neurons [47, 48] in neurodevelopmental disorders, we report robust evidence for an association between non-neuronal cell types (astrocyte and oligodendrocyte) and a subgroup of ASD de novo LOF variants. The observed restricted non-neuronal cell expression among ASD candidate genes suggests molecular evidence that deficits in non-neuronal function are implicated in ASD.

Our results showed robust association of non-neuronal cell types with de novo ASD LOF variants. Moreover, this association was found to be conserved in multiple rodent datasets. Our non-neuronal cell clusters are small in size, but the association was replicated across clusters in all three brain regions studied, particularly in view of their potential role in neurodevelopmental disorders more generally as discussed subsequently. Moreover, the small size of the clusters is perhaps unsurprising given that the Allen Brain Atlas has neuronal bias with approximately 90% neuronal cells. Our analysis also revealed that DEGs in the large single-cell clusters are also enriched for de novo ASD LOFs; are neuronal in identity; and that the size of these clusters is larger compared to the top three ASD de novo LOF enriched clusters. Overall enrichment analysis revealed that ASD de novo LOF mutated genes have molecular aetiology consisting of multiple neuronal subtypes and a molecular subtype of ASD restrictively expressed in non-neuronal brain cells. Given our results, we hope there is now an impetus to look beyond neuronal cells in the pathogenesis of ASD.

Our spatiotemporal analysis of the top brain cell type clusters shows DEGs are highly active in prenatal stage and within DFC and V1C regions of the brain. The prenatal molecular origin of ASD has been confirmed in multiple independent studies [12, 28, 49]. ASD is often associated with early brain overgrowth, particularly involving the prefrontal cortex [50], which plays a central role in mediating working memory [51] and context-dependent prioritisation of off-task thought [52]. Visual hypersensitivity is a common sensory deficit in ASD, which may impact behaviour and learning [53]. Hypersensitivity to visual stimuli observed in ASD is caused by altered connectivity in visual pathways and attention networks, thereby contributing to social communication vulnerabilities [54]. Gaze aversion and lack of joint attention, core diagnostic elements of severe ASD, are both mediated by incoming visual information [55]. The enrichment of ASD mutations in V1C clusters therefore aligns with the early clinical phenotype, and our data therefore support the need to investigate V1C and its functional connections with these early clinical traits observed in ASD patients. Similarly, DFC pathways are often impacted in ASD, either in the context of its comorbidity with ADHD or its wider association with executive dysfunction. ADHD, a disorder of attention and behavioural control, is largely mediated by DFC pathways and is diagnosed in more than 40% of children with ASD [56]. Moreover, prefrontal cortical regions have always been strongly implicated in ASD in relation to both their role in decision-making and social behaviour, and the patterns of impairments demonstrable by imaging [57]. To identify the molecular convergence, we have conducted cluster specific pathway enrichment analysis. Interestingly, our results on LOF mutated genes show major biological pathways enriched for the top clusters that include synaptic vesicle, transmembrane ion activity networks, development and metabolic processes, cell growth and actin cell regulation.

Unlike neurons, the glial cells (including astrocytes and oligodendrocytes) do not transmit signals, but they constitute a ‘supportive’ environment to chaperon the neurons and shape the neuronal network [58, 59]. It is unsurprising, therefore, that their dysfunction has been described in brain diseases such as schizophrenia, ASD and Alzheimer’s Disease [60–62]. Astrocytes, for example, are the most abundant cell type in the central nervous system and are involved in a wide variety of specific functions, including axonal guidance, response to inflammation, wound healing and the construction of the blood brain barrier [43, 63]. Moreover, the functional neural circuit involves numerous types of cells, with astrocyte as one of the key cell types for synapse formation and function [64, 65]. Similarly, the myelin forming oligodendrocyte has recently been shown to express post-synaptic proteins. Glial cells (astrocytes and microglia) are vital in modulating neural connectivity during development, and glial dysfunction has been hypothesised to be a key contributor to the development of ASD [66]. However, to date no systematic study has been undertaken to elucidate the role of genes regulating glial cells in either ASD or related neurodevelopmental phenotypes.

Several studies have already identified aberrant non-neuronal mediated process in ASD. For example, it was previously reported that microglial activation in ASD was associated with a neuron-specific reaction in the dorsolateral prefrontal cortex [67]. This is consistent with the findings that abnormal activation of microglia and astrocytes occur in multiple brain regions of autistic patients [68–70]. A recent study reported that an elevated synthesis of proteins in microglia causes social impairments, cognitive deficits and repetitive behaviour in male mice, each an important component of the ASD phenotype [71]. Since immune molecules and cells such as microglia play a role in synaptic development and function [72], the observed immune up-regulation may be related to abnormal ongoing plasticity in ASD brain [73]. Transcripts upregulated in autism were preferentially involved in immune functions, while transcripts downregulated in autism were involved in neural functions, including calcium signalling and long-term potentiation pathways [74]. These studies, therefore, provide important clues to the role played by non-neuronal cells in disease, but much remains to be learned about their involvement in the pathophysiology of ASD and related disorders.

Considering the genes that underlie these non-neuronal processes, by applying single-cell transcriptomics it is now possible to find cell type-specific roles for genes. The largest published autism RNA-seq (from bulk tissues) study of post-mortem brain tissue found evidence for cortex-specific differential gene expression and alternative splicing events, with enrichment for genes expressed in microglia and astrocytes [9]. Our study showed that such glial cell type regulatory genes are also harbouring de novo LOF variants in a subgroup of ASD individuals.

We further validated these results using gene expression databases with complementary brain cell types, where cells were pre-sorted based on known markers. Validation of non-neuronal expression bias of cluster genes intersected with LOF genes in mouse indicates broad conservation of core brain cellular functions across species. We further identified few non-neuronal bias genes (*PLXNB1, KANK1, TANC2, GLUL*) from our study which were among the top fold change genes across three brain regions. Multiple variants in *PLXNB1* and *KANK1* in ASD cases were reported and these genes were prominently expressed in astrocytes compared to neurons in both human and mouse. Transcriptional profiling experiments indicated that *PLXNB1* is expressed in both neurons and glial cells in the cerebral cortex, with the highest expression found in glia [41, 75]. Furthermore, this gene is expressed in inhibitory and excitatory neurons and glia in the developing hippocampus [76]. *KANK1* is reported to be associated with cerebral palsy, and many CP cases are also usually reported to have autism and other NDD.

Since ASD research so far has principally been focused on neurons and not the non-neuronal cell types, future studies should now examine the role of other cell types in identifying disease mechanisms of ASD. Here, we have provided evidence that different functions in different cell types may be dysregulated in ASD; investigating functional interactions between ASD candidate genes in different cell types in normal human brains may provide new insight into the genetic heterogeneity of ASD. Specifically, we suggest that different cell types may play unique roles in the pathogenesis of the disorder. Also, cell clusters driving the association of ASD appear to be similar to those of fMRI/epilepsy. Although each of these conditions has been previously linked to ASD, our findings might help further define an emerging molecular subtype of ASD.

As in oncology, where the identification of cancer subtypes enabled the development of effective targeted treatments [77–79], the identification of molecular ASD subtypes is expected to result in similar opportunities for therapeutic development. Overall, the work shown in the present study represents a proof of concept for the value of using massive amounts of multimodal data to push the boundaries of existing knowledge, thereby moving us closer to precision medicine for ASD.

## Conclusions

In summary, our integrative approach, incorporating multi-dimensional transcriptomic data from different sources, has facilitated an entirely novel understanding of the potential brain architecture in ASD. First, we observed that genes forming a natural cluster tend to have shared functions in different brain regions. And secondly, we showed that non-neuronal cell types may be implicated in a molecular subtype of ASD. In conclusion, by using an integrative framework, we were able to examine the convergence of clinical mutations onto specific disease-related pathways. The robust analytical framework provided in this work might be used to uncover functional modules for other genetic diseases, improving their risk assessment. The convergence of molecular subtypes of ASD risk genes to brain cell types and pathways will be crucial for the future development of more effective ASD diagnosis and therapeutics by targeting relevant cell types associated with ASD.

## Methods

### Data collection

The data were compiled from 26 published cohort studies to extract reported mutations for further analysis. Studies include reported mutations from MSSNG, Autism Sequencing Consortium (ASC), Simon Simplex Consortium (SSC) and other studies with population cohort reports on autism spectrum disorder that applied exome, whole genome or targeted sequencing approaches. This resulted in a total of 169,580 genetic mutations/variants reported by all the articles that include 156,688 de novo mutations/variants impacting autism candidate genes. These variants were reported from 40,122 cases with ASD collectively from all the exome/genome sequencing cohort studies. A summary of all key information from 26 studies is listed (Additional file 2: Table S1).

### Single-cell transcriptome data

We obtained publicly available single-cell RNA-seq data from Allen Brain Atlas (https://portal.brain-map.org/atlases-and-data/rnaseq), which were created from intact nuclei derived from three brain regions, viz. ACC (Anterior Cingulate Cortex), MTG (Middle Temporal Gyrus) and VISP (Primary Visual Cortex). 8 human tissue donors ranging in age from 24 to 66 years were analysed (ACC-7,283 nuclei; MTG-15,928 nuclei; VISP-8,998 nuclei). Nuclei were sampled from postmortem and neurosurgical (MTG only) donor brains and expression was profiled with SMART-Seq v4 or 10× Genomics Chromium Single-Cell 3' v3 RNA-sequencing. Raw read (fastq) files were aligned to the GRCh38 human genome sequence. For alignment, Illumina sequencing adapters were clipped from the reads using the fastqMCF program. After clipping, the paired-end reads were mapped using Spliced Transcripts Alignment to a Reference (STAR) using default settings. Quantification was performed using summerizeOverlaps from the R package GenomicAlignments. Expression levels were calculated as counts per million (CPM) of exonic plus intronic reads. Intronic and exonic read counts were summed, and log2-transformed expression was centred and scaled across nuclei.

### Dimensionality reduction, clustering and t-SNE visualisation

The filtered single-cell RNA seq data from ACC, MTG and VISP regions were used for unbiased clustering using Seurat v.3 [23]. Seurat is an R toolkit designed for QC, analysis and exploration of single-cell transcriptomic analysis. Highly variable genes (2000 features) were found using Seurat object FindVar and these were scaled up after applying linear transformation regressing on the percentage of mitochondrial reads (Additional file 1: Fig. S5). Principal component analysis was performed to reduce the dimensionality of the data by RunPCA using the highly variable features. Elbow Plot was used to identify the number of significant PCA for downstream analysis by localising the last PC before the explained variance reaches plateau (Additional file 1: Fig. S5). First 24, 22 and 24 PCs for ACC, MTG and VISP regions, respectively, were used as input in FindNeighbors to construct KNN graphs using PCs. Clusters were created using FindClusters (resolution = 0.8, 0.5, 0.6 for ACC, MTG and VISP regions, respectively). To visualise nuclear transcriptomic profiles in two-dimensional space, t-distributed stochastic neighbour embedding (t-SNE) [80] was performed with the selected PCs and perplexity = 30. Further, DimPlot using reduction = tsne was plotted (Fig. 1A).

### Gene set enrichment

For ASD de novo mutated genes, we have created multiple sets of genes, that includes i) all genes impacted by de novo LOF variants in ASD ii) multiple de novo LOF genes ii) multiple de novo missense and iii) multiple de novo or missense (union) variants in ASD. In addition, we curated all FMRP1 targeted gene list [81], epilepsy and intellectual disability genes from SFARI. Only unique genes were retained for each gene set for enrichment analysis. We have used control set (housekeeping genes) with non-brain expressed genes (non-critical exon) and low pLI score (0.05). We tested whether different gene sets were enriched in clusters using GeneOverlap package in R and used Bonferroni method for multiple corrections.

### Cluster annotation to define cell identity

We used known marker genes to map clusters to cell types, minimising potential bias due to differential expression of individual genes. We determined the brain cell types in each of the clusters by evaluating the expression of known marker genes for neurons, astrocytes, oligodendrocytes, microglia, OPC (Additional file 3: Table S13), obtained from literature by performing unbiased gene marker analysis. Differentially expressed genes were calculated by using four tests (Wilcox, t test, Bimod, MAST) in each cluster and were used to analyse cell type markers. Cluster genes overlapping with marker genes were used for further analysis. We used boxplot to visualise the average expression of genes related to specific cell types, donut plot to display the composition of top 20 DE genes and Featureplot to visualise the expression of a marker gene related to specific cell type. Clusters were annotated based on the Boxplot, Dotplot and Featureplot.

### Spatiotemporal expression data from human brain

We downloaded normalised RNA-seq data for spatiotemporal expression profiles of human brains from the BrainSpan database (http://www.brainspan.org/static/download.html). The data set used consisted of spatiotemporal expression profiles from 42 donors across 26 regions from the BrainSpan database [82]. Those regions found in more than 2 donors were retained (16 regions) and a total of 15,55,39,169 brain expression data point was used for analysis. Donors were selected so that each developmental period included at least two age- and sex-matched donors. The developmental periods were categorised into three groups: prenatal (8 to 37 weeks post-conception), early childhood (10 months to 15 years) and adulthood (> 17 years). For each donor, we obtained expression data from 16 brain regions within the 3 developmental periods. Illumina Genome Analyser II’s (GAIIx) was used for RNA sequencing, reads were aligned and mapped to Reference genome (Gencode v10), and normalising was carried out according to sequencing depth and size of the element based on the RSEQtools framework [83]. The expression level of genes, exons and spike-in RNAs were measured in the commonly used units of RPKM (reads per kilobase of exon model per million mapped reads) [84].

### Constraint single-cell brain cluster analysis

Critical exons (CE) are a measure that computes exon level burden of non-synonymous mutation and brain expression [28]. CE computes the correlation between mutation burden and brain expression to identify exons that are conserved for mutation accumulation and high in brain expression. For CE computation, for each exon we have computed exon level expression from 42 brain spatiotemporal transcriptomic samples (as described before) and used gnomAD to compute non-synonymous mutation burden normalised by the exon length. Next, we used pLI score, a second method to identify constraint single-cell brain cluster genes. To quantify statistical significance, we have used R package and applied proportion test and Fisher’s exact t-test for CE and pLI enrichment, respectively.

### Pathway analysis

Analysis of the genetic data in the mutation database created was subsequently done by biological pathway analysis to identify the biological pathways affected by the mutations in the database. The analysis utilised the KEGG pathway database which is a collection of manually drawn pathway maps representing the current knowledge on the molecular interaction, reaction, and relation networks (http://www.genome.jp/kegg/pathway.html) and GO database (http://geneontology.org/). In this analysis, we assessed the overlap between the mutated genes in our database and the KEGG-GO pathways. Pathway gene sets less than 50 and greater than 1000 were excluded. If an overlap between our dataset and a KEGG-GO pathway is significant based on the FET, it is said to be enriched; hence, we evaluated the enrichment of genes in KEGG pathways against a common background consisting of all the genes in the data set. The pathways are identified by their name and unique KEGG ID. The significantly enriched set (Fisher’s exact test, *p* < 1.0 × 10^−3^ and FDR < 0.01) was used for network construction. Cytoscape (https://cytoscape.org/) which is a visualisation tool was utilised to map and construct a network of the biological pathways associated with ASD. The networks were plotted with colour gradient and size of node representing p-value and odds ratio, respectively. GSEA [85] was used for functional enrichment analysis.

### Cell type-specific gene expression dataset for validation

We have used independent single-cell transcriptome datasets from human and mouse brain regions to replicate our findings. Single-cell RNA sequencing data of 466 cells with known (sorted using known primary brain cell markers) cell type (neurons-131, astrocytes-62, oligodendrocytes-38, microglia-16, OPC-18) were used [40] to study the cell fate of cluster genes. We filtered out any genes which were not expressed in more than 75 percentiles of the cells and computed the mean expression for each gene in gene sets of interest (LOF genes, cluster genes intersected with LOF genes) across cell type from the expression data. Fold change was calculated with respect to neurons and the expression of top 10 most fold change genes were also plotted.

The replication datasets were a mouse study that sampled purified neurons, astrocytes, oligodendrocyte precursor cells, oligodendrocytes and microglia from mouse cerebral cortex [41]. We used the pre-computed mean expression data and plotted the expression of top 10 most fold change genes. Other datasets used were nuclear transcriptional data of the nervous system using flow sorting of genetically labelled nuclei [42], RNA-Seq of human astrocytes [43]. For quantifying enrichment of ASD LOF variants across brain cell types, DE genes from 44, 428 cells of cerebellum and 39,495 cells of cerebrum (fetal) were used [44]. Fetal brain data collected from 59 samples were sequenced using sci-ATAC-seq3, and gene expression data collected on an overlapping set of tissues were leveraged to annotate cell types. We used pre-computed gene expression data and plotted the mean expression of genes across brain cell types. Student’s t-tests were used to compare the mean expression and fold change, and Fisher’s exact test was used to examine the association.

## Supplementary Information

**Additional file 1.** Additional methods, dataset availability, Supplementary Figures, 1-27.

**Additional file 2.** Supplementary Tables - 1, 2, 3, 5, 6.

**Additional file 3.** Supplementary Tables - 4, 10, 13, 14, 17, 18, 19

**Additional file 4.** Supplementary Tables - 7, 8, 9, 11, 12.

**Additional file 5.** Supplementary Table - 15.

**Additional file 6.** Supplementary Table - 16.

## Data Availability

The datasets supporting the conclusions of this article are included within the article (and its Additional files).
