# Supplementary material for: Single-cell transcriptome identifies molecular subtype of autism spectrum disorder impacted by de novo loss-of-function variants regulating glial cells
Source: Hum Genomics. 2021 Nov 21;15:68. doi: 10.1186/s40246-021-00368-7 (PMC8607722; doi:10.1186/s40246-021-00368-7)
Supplement: Supplementary file 1 — Additional file 1. Additional methods, dataset availability, Supplementary Figures, 1-27. [file 40246_2021_368_MOESM1_ESM.docx]

**Supplementary Document:**

**Single cell transcriptome identifies molecular subtype of autism spectrum disorder impacted by *de novo* loss of function mutation genes regulating glial cells**

Nasna Nassir, ^1#^ Asma Bankapur, ^1#^ Bisan Samara, ^2^ Abdulrahman Ali, ^1^ Awab Ahmed, ^1^ Ibrahim Inuwa, ^1^ Mehdi Zarrei, ^3,4^ Seyed Ali Safizadeh Shabestari, ^1^ Ammar AlBanna, ^1^ Jennifer L. Howe, ^3,4^ Bakhrom Berdiev, ^1^ Stephen W. Scherer, ^3,4,5^ Marc Woodbury-Smith, ^3,6^ Mohammed Uddin^1,7^*

1. College of Medicine, Mohammed bin Rashid University of Medicine and Health Sciences, Dubai, UAE

2. Biomedical Engineering Department, McGill University, Montréal, QC, Canada

3. The Centre for Applied Genomics (TCAG), The Hospital for Sick Children, Toronto, Ontario, Canada

4. Genetics and Genome Biology, The Hospital for Sick Children, Toronto, Ontario, Canada

5. Molecular Genetics, University of Toronto, Ontario, Canada

6. Biosciences Institute, Newcastle University, Newcastle upon Tyne, UK

7. Cellular Intelligence (Ci) Lab, GenomeArc Inc., Toronto, ON, Canada

# Contributed equally to this work

*Corresponding Author

Mohammed Uddin, PhD

College of Medicine, Mohammed bin Rashid University of Medicine and Health Sciences, Dubai, UAE

Email: [mohammed.uddin@mbru.ac.ae](mailto:mohammed.uddin@mbru.ac.ae)

**Data collection.** Our goal was to use ASD mutation data to identify appropriate cell types implicated in ASD. We thus obtained relevant genetic variants associated with ASD through extensive literature search from peer reviewed original articles. The search was done using PubMed and Google scholar. The articles selected fulfilled these inclusion criteria: original articles (written in English language) investigating genotypes and phenotypes of individuals who were primarily diagnosed with ASD (study types included case-control studies, family-based studies, case-studies and case reports) published recently (post 2010). Studies that re-analysed previously published data were excluded. Articles reporting genetic variants of individuals with other disorders (epilepsy, schizophrenia etc.) as a primary diagnosis with ASD as a co-morbidity were also excluded. The search was not restricted to any study design, time, type of genetic variant or inheritance pattern (inherited or *de novo*). The keywords such as ‘autism gene’, ‘autism variants’, ‘autism mutation’, ‘autism sequencing’ etc. with appropriate Boolean operators (AND, OR, NOT) were used for the search in PubMed and Google Scholar.

A data extraction sheet was used to list all the relevant information such as the article information (title, PMID etc.) and variant information (WGS/WES/other, cohort number, origin, etc.). A sample of the data extraction sheet is presented (Supplementary Table 1). Subsequently, the core genetic mutations that correlated specifically with ASD were identified from each article. To ensure consistency, all genomic coordinates from different studies were converted to the most recent genome build version (GRCh38/hg38) using the UCSC lift over tool. The majority (>98.0%) of coordinates were converted successfully, but few coordinates failed due to region deletion in hg38, hence these were excluded. Genomic coordinates were inferred from the RefSeq IDs using TransVar [1] web tool in case they were not explicitly reported in the study, which was the case in [2]. Our list contained each mutation/variant reported in the obtained articles (in ASD patients) together with its location in the genome (chromosome number and coordinate). After build conversion, functional annotation of all genetic variants was conducted in ANNOVAR [3] using the hg38 version of the databases listed (Supplementary Table 1). ANNOVAR input file included the following information in tab-delimited format: chromosome number, variant start and end coordinates, reference (ref) and alternative (alt) nucleotides. If the nucleotide information was not available, the ref and alt columns were filled with zero “0”, and a dash “-” was used to indicate an Indel. Entrez IDs were then mapped to gene symbols using R.

**Population.** A close examination of the data shows multiple salient characteristics of the sample which are summarized in Supplementary Figure 1. Most children sequenced (around 44%) presented primarily with ASD phenotype plus DD (developmental delay) as a comorbidity and around 28% of sequenced children had the phenotype of ASD alone. These two phenotype categories contributed to 72% of the sample with the rest of the 28% being distributed among ASD phenotypes mixed with Intellectual disability (ID), Schizophrenia (SZ) and other disorders. Most of the studies reviewed were family-based studies (50%) in which parents and at least two of their offspring were sequenced and case-control studies (27%) where cases of ASD patients were compared to controls. In terms of ethnicity, most of the articles did their study on Caucasian population (18 out of 26, the articles did not specify but the studies were set up in Western countries). In other 8 studies, patients with other ethnicities were sequenced and the distribution can be seen in Supplementary Figure 1. The sequencing methodology used by the studies were mostly WES (whole exome sequencing), and WGS (whole genome sequencing) with some studies using High Resolution Melt (HRM) and Molecular Inversion Probes (MIP) (Supplementary Figure 1).

**Single cell transcriptome data.** For the processing of RNA-seq data matrix, cells which have more than 99% 0’s in all genes were filtered out, and all genes which have more than 99% 0’s in all cells were filtered out.

Summary statistics after quality control

| **Reading matrix** | **Original matrix** | **Filtered matrix** |
| --- | --- | --- |
| ACC | (50281, 7283) | (25572, 7271) |
| MTG | (50281, 15928) | (27823, 15928) |
| VISP | (50281, 8998) | (23824, 8967) |

**Dimensionality reduction, clustering and t-SNE visualization.** The filtered single cell RNA seq data from ACC, MTG and VISP regions were used for unbiased clustering using Seurat v.3 [4]. ERCC abundances on the raw counts were calculated before creating a Seurat object by gripping on ^ERCC and ERCC was removed from count.data. After creating the Seurat object, cells which have unique feature counts over 9000, 11000, 11000 and less than 150, 1000, 300 were filtered out for ACC, MTG and VISP regions respectively (Supplementary Figure. 5). Then, the percentage of MTN genes were derived and cells with mitochondrial counts greater than 0.15, 0.4, 0.25 were filtered out for ACC, MTG and VISP regions respectively (Supplementary Figure 5).

**Enrichment of cluster genes with high pLI genes.** pLI scores were obtained from Exac database and it reflects the tolerance of a given gene to loss of function and pLI ≥ 0.9 are extremely LoF intolerant. We did enrichment analysis of genes with high pLI scores viz highly intolerant genes with cluster genes (GeneOverlap) and plotted p value as the gradient, OR as the width and overlap size as the radial diameter.

**Exome data.** Critical exon data was obtained from Allen brain institute (RNAseq Gencode ver10 exon data). We used data from the ExAC database [5] to calculate the burden of LoF mutations (splice acceptor variant, splice donor variant, stop gain, frameshift variant, stop loss, start loss, inframe insertion, inframe deletion, missense variant, protein altering variant, splice region variant) in human populations (variants were obtained from 60,706 exomes).

**Burden of rare missense mutations.** Mutation burdens were defined as the ratio between the number of mutations in an exon to the exonic length in bp (base pair).

**Brain critical exon data.** The expression data for exons were obtained from Abi database and the mutation scores from Exac database. Burden score was calculated as mutational frequency per length of exon. Exons were classified as critical exons if the expression value was >75th percentile of expression data (6.388519) and <75th percentile burden score (0.09541985)). Number of CE = 28940649, Number of Non CE = 126598520, Percentage of critical exons= (28940649/155539169)*100 = 18.60666299432267.

Although many psychiatric and neurological disorders have onsets in adolescence, some have onsets earlier (autism) or later (Alzheimer’s and Parkinson’s disease). It is thus important to map cluster genes at specific developmental times. To check which brain region and stage the cluster genes are active, we looked for enrichment of ‘brain-critical exons’ in ASD LOF enriched clusters in prenatal (PN) vs adulthood (AD) and early childhood (EC) vs AD. Odds ratio was plotted as gradient onto the brain landscape displaying 16 brain regions.

**DNA replication timing analyses.** We used data from replication domain database [6] for NPC differentiation type BG01 cells. The average replication timing was calculated and annotated using Gencode ver 32. Averaged replication scores per gene were computed and then mapped the respective genes from the gene list. We defined early replicating genes as genes that fall within regions with replication timing Z≥75th percentile of positive values and late replicating genes as genes that fall within regions with replication timing Z≤75th percentile of negative values. Gene Overlap of early and late replication timing genes with cluster genes were carried out.

**Data Availability**

The following previously published datasets were used:

1. A survey of human brain transcriptome diversity at the single cell level (Darmanis et al 2015), Publicly available at the NCBI Gene Expression Omnibus (accession no. GSE67835)
2. An RNA-Seq transcriptome and splicing database of neurons, glia, and vascular cells of the cerebral cortex (Zhang et al., 2014), Publicly available at the NCBI Gene Expression Omnibus (accession no. GSE52564)
3. A Comprehensive Analysis of Cell Type-Specific Nuclear RNA From Neurons and Glia of the Brain (Reddy et al., 2017), Publicly available at the NCBI Gene Expression Omnibus (accession no. GSE73391)
4. Purification and Characterization of Progenitor and Mature Human Astrocytes Reveals Transcriptional and Functional Differences with Mouse (Zhang et al., 2016), Publicly available at the NCBI Gene Expression Omnibus (accession no. GSE73721)
5. A human cell atlas of fetal gene expression (Cao et al., 2020), Publicly available at descartes.brotmanbaty.org.

**Supplementary figures**


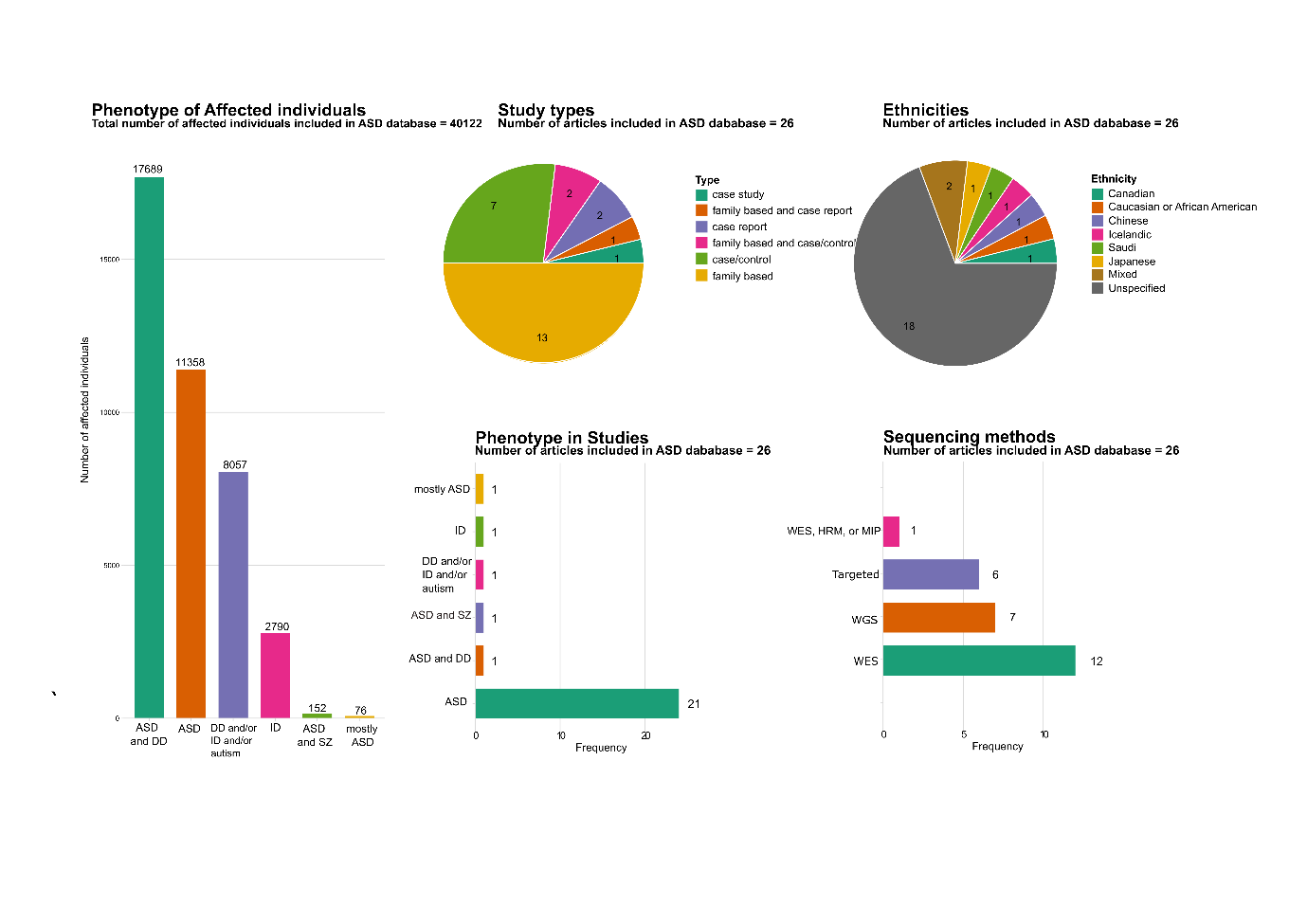


**Supplementary Figure. 1| Summary of ASD mutation data collected from literature.** A) Phenotype distribution of affected individuals included in the study. B) Pie chart displaying the study types of the articles included. C) Pie chart displaying the ethnicities of affected individuals. D) Frequency of articles phenotype wise. E) Frequency of sequencing methods used in the articles.


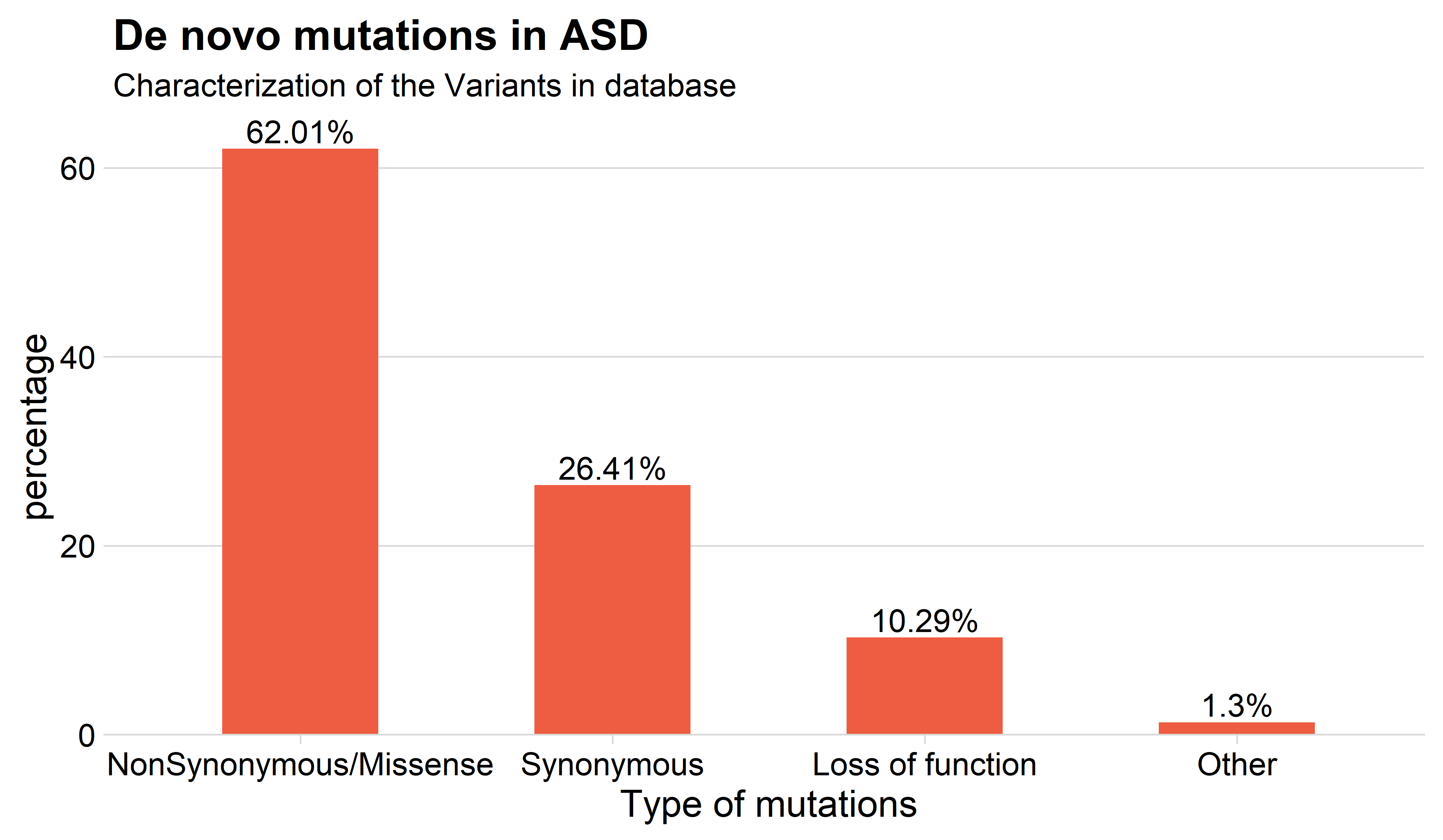


**Supplementary Figure. 2| Composition of *de novo* ASD mutation genes.** Percentage of ASD mutation genes across missense, synonymous and LOF.


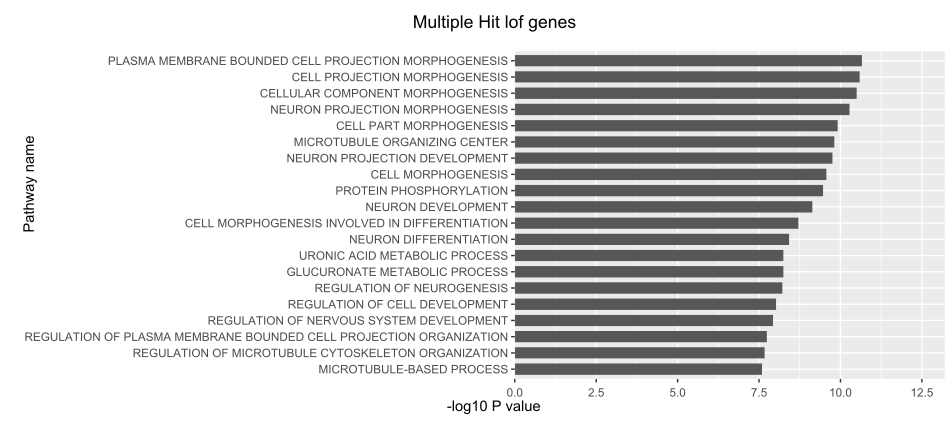


**Supplementary Figure. 3| Top biological pathways enriched for ASD LOF genes.** Y axis represent pathways enriched for ASD LOF genes and x axis represents the associated statistical significance as −log10 (P) of z statistics.


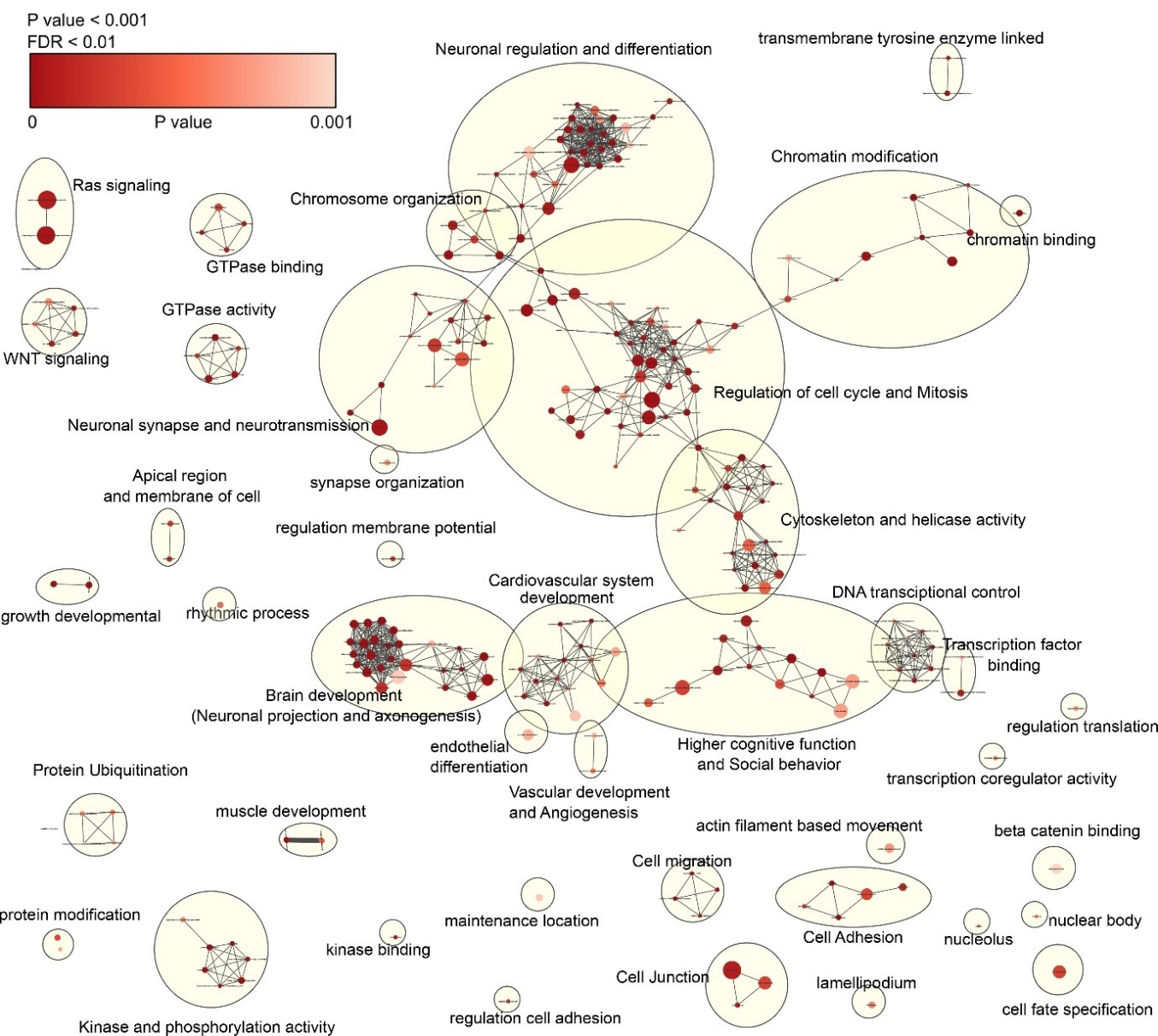


**Supplementary Figure. 4| Pathway visualization of ASD LOF genes.** Pathway network analysis in ASD LOF genes drawn using Cytoscape. Colour gradient and size of nodes are represented by pvalue and Odds ratio, respectively.


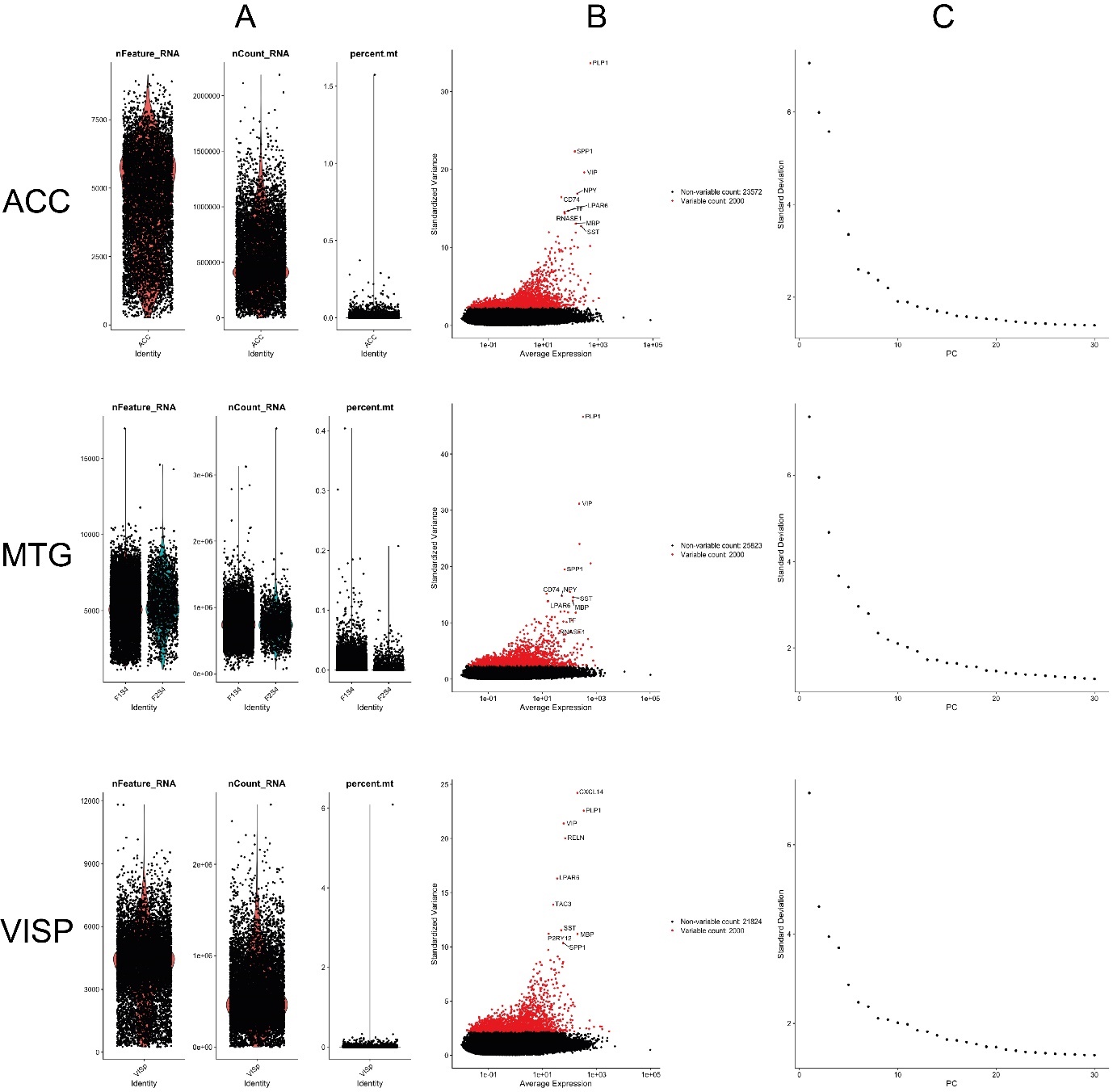


**Supplementary Figure. 5| Processing of RNA seq data using Seurat.** A) Feature counts, mitochondrial counts (QC). B) Highly variable genes. C) Elbow plot to identify significant PCs.


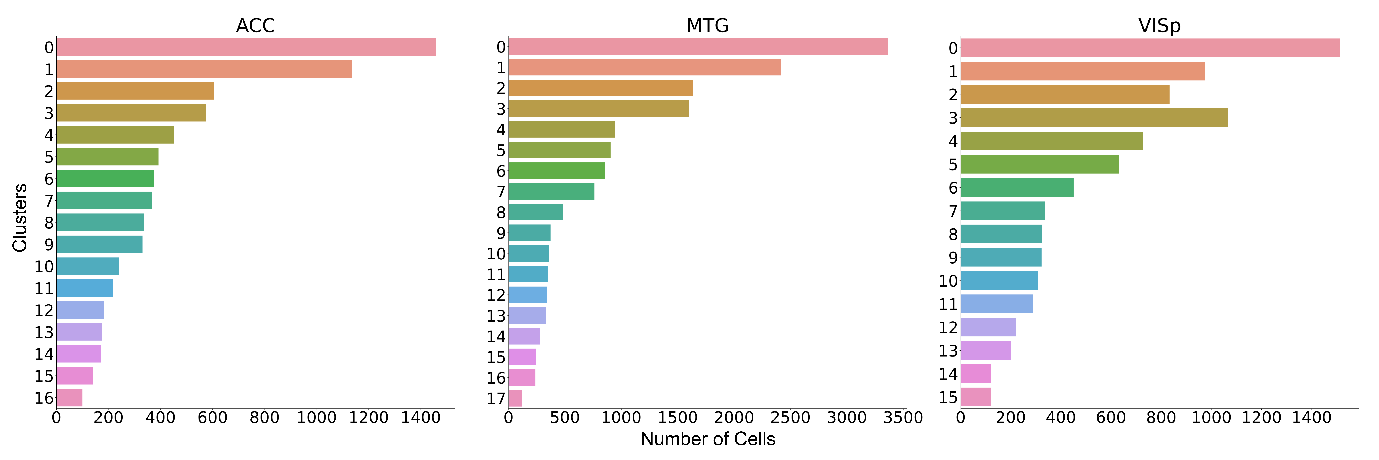


**Supplementary Figure. 6| Number of cells in each cluster across ACC, MTG and VISP.** Y axis represent clusters and x axis represents number of cells.


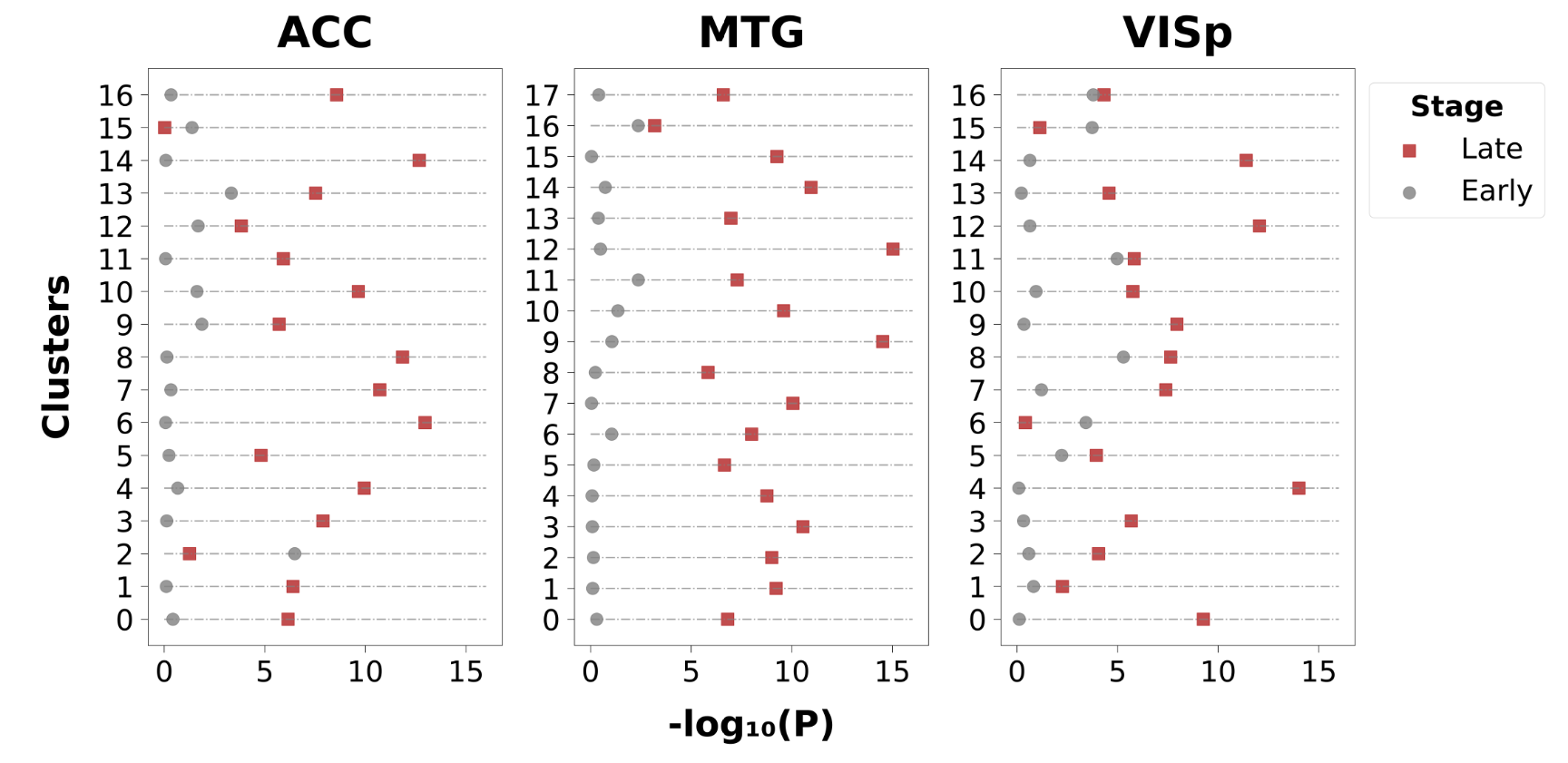


**Supplementary Figure. 7| Enrichment of replication timing genes across clusters.** Y axis represent clusters and x axis represent enrichment associated -logP value. Late replication timing genes (red) are enriched in cluster genes compared to early replication genes (grey).


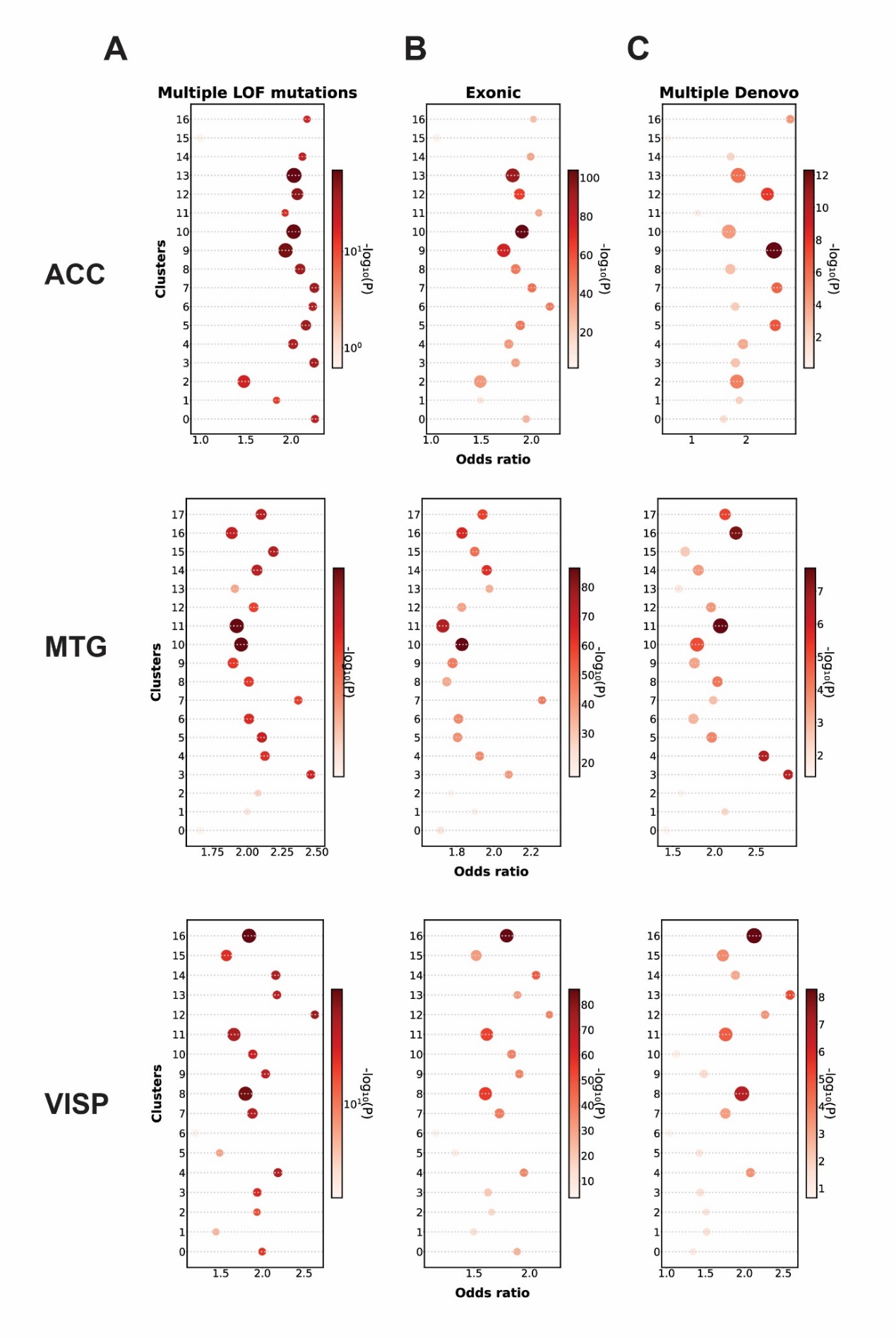


**Supplementary Figure. 8| Enrichment of ASD (A) multiple LOF, (B) exonic, and (C) multiple *de novo* mutation genes in ACC, MTG and VISp regions.** The y axis here represents clusters, x axis odds ratio, the size of the circle by overlap gene size and the gradient represent p-value.


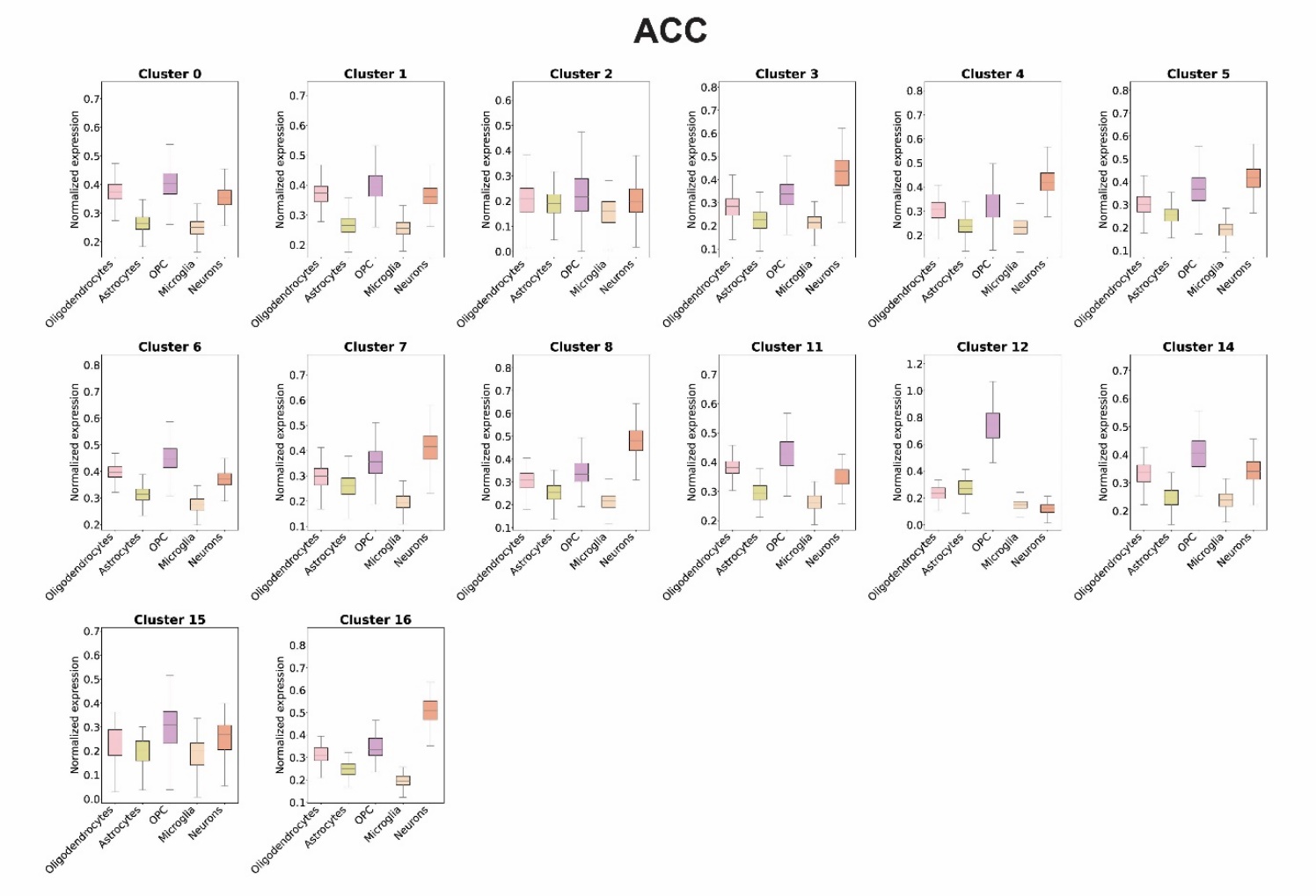


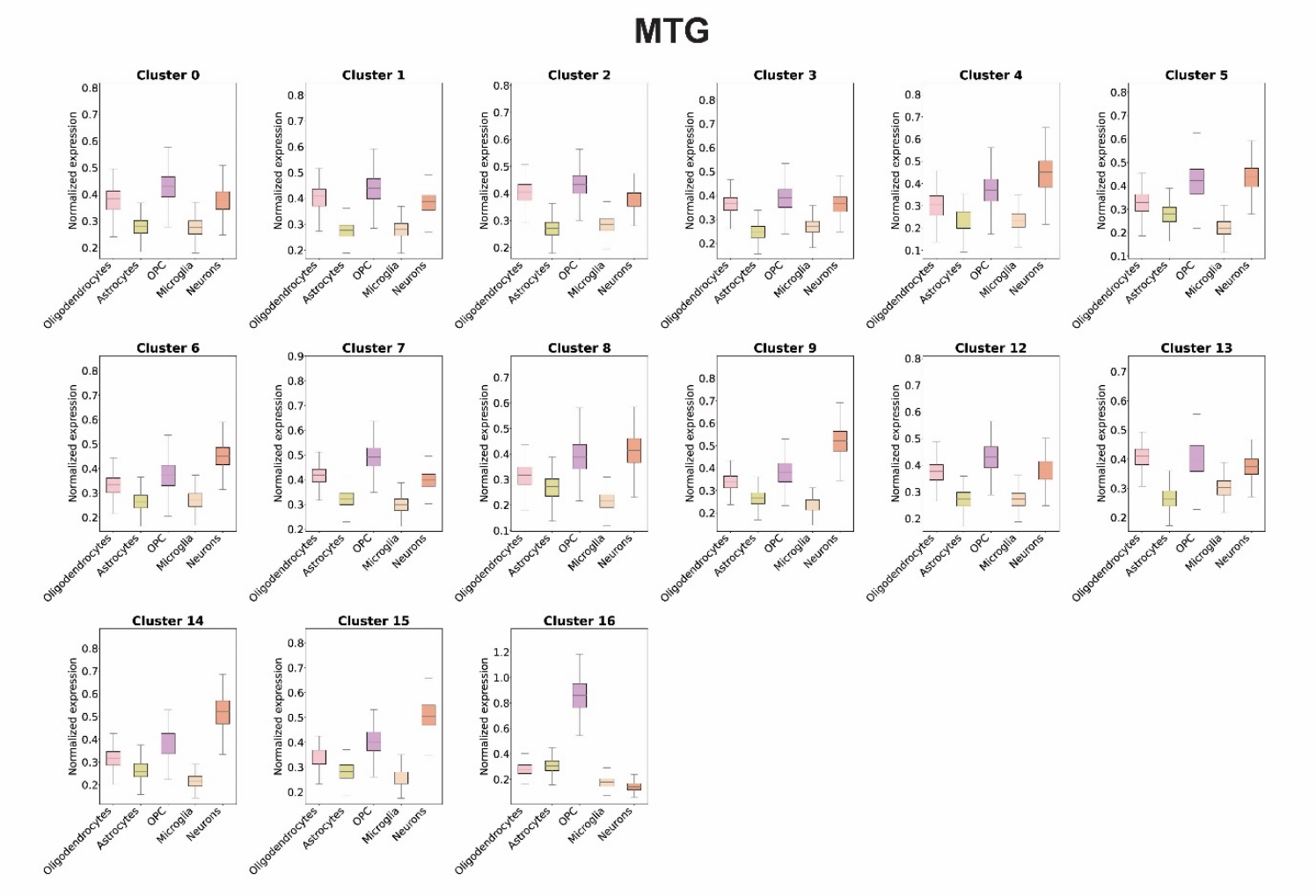


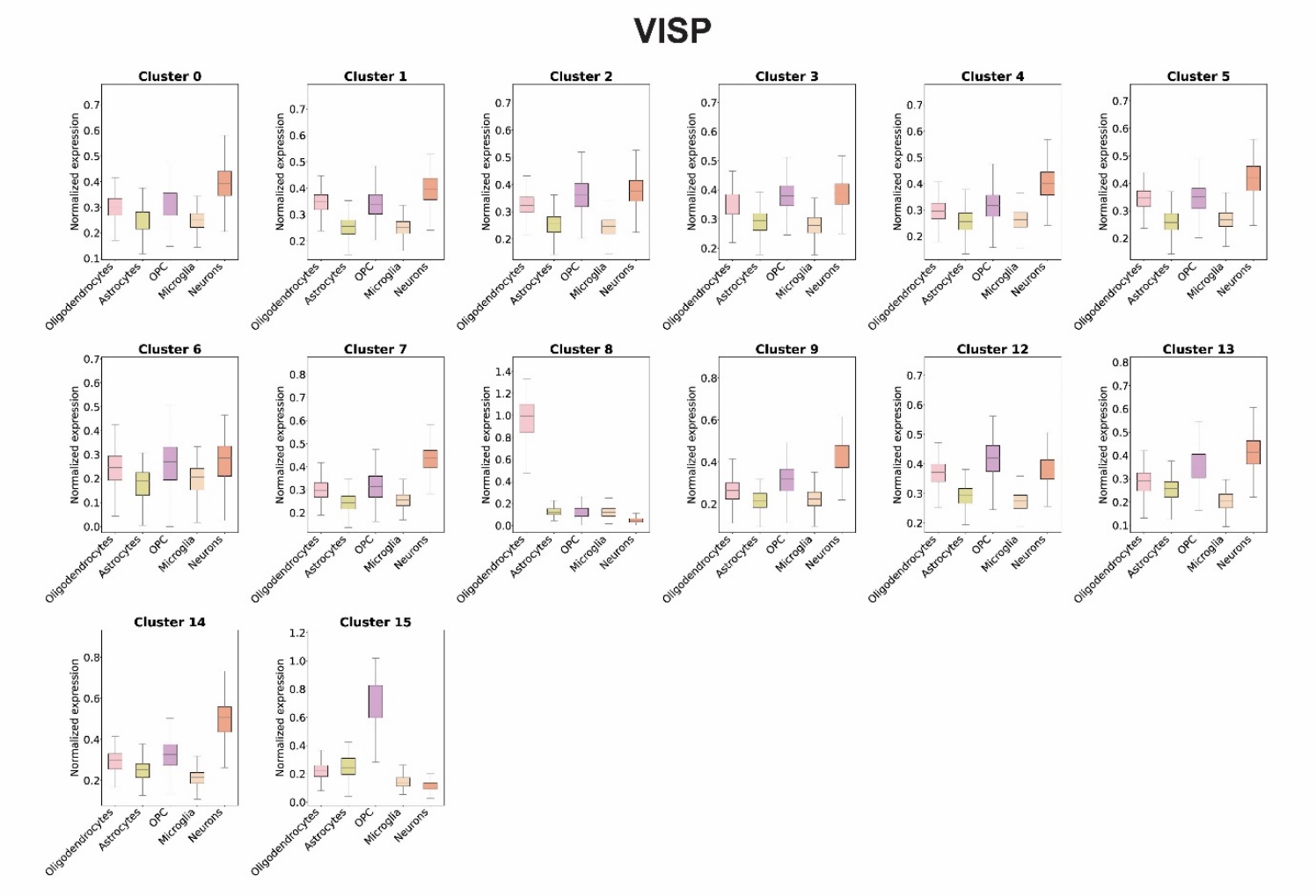


**Supplementary Figure. 9| Boxplot of expression of marker genes for all clusters.** Normalized expression of marker genes in cluster genes across oligodendrocytes, astrocytes, OPC, microglia, neurons in ACC, MTG and VISp regions.

**ACC**
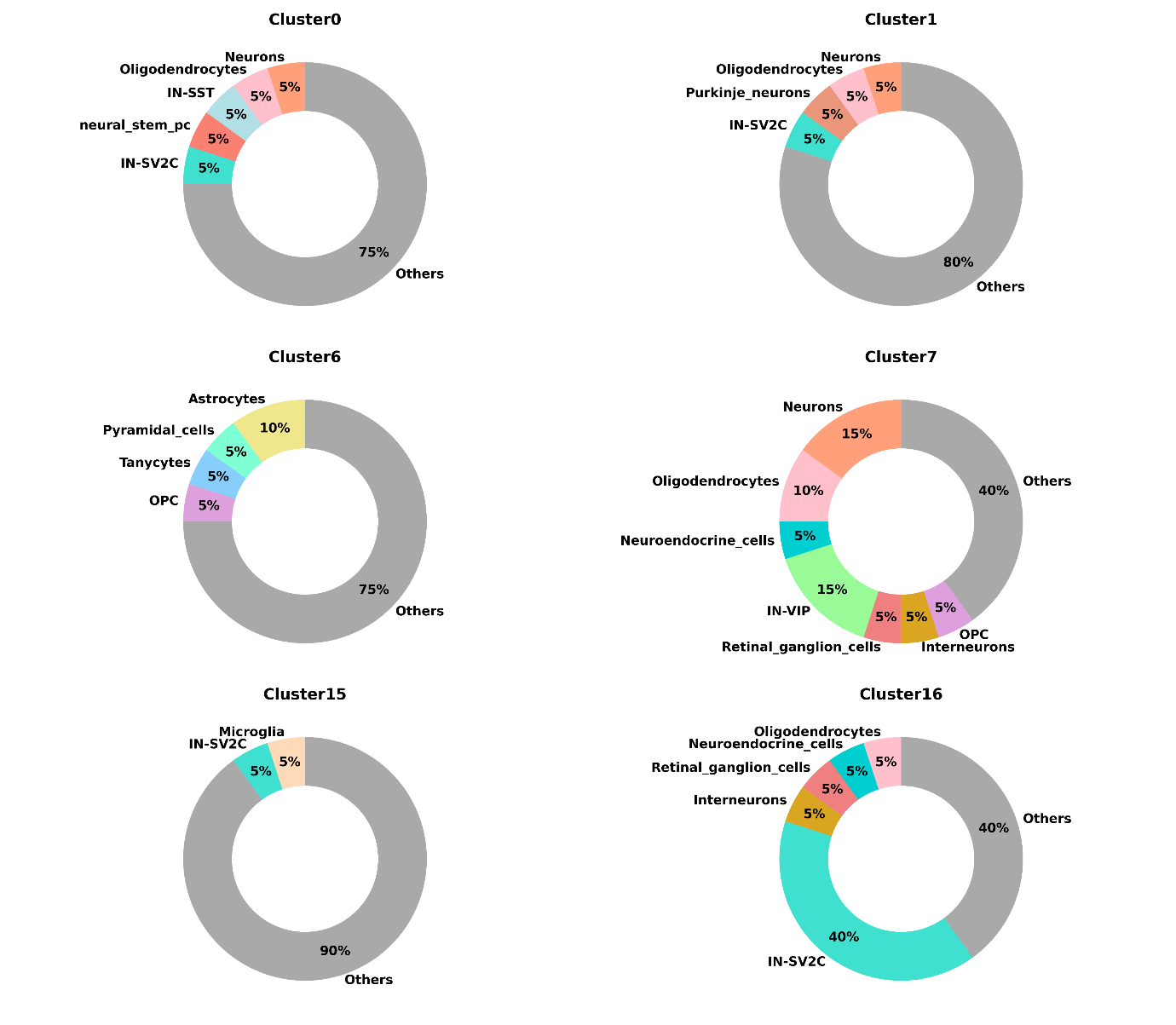

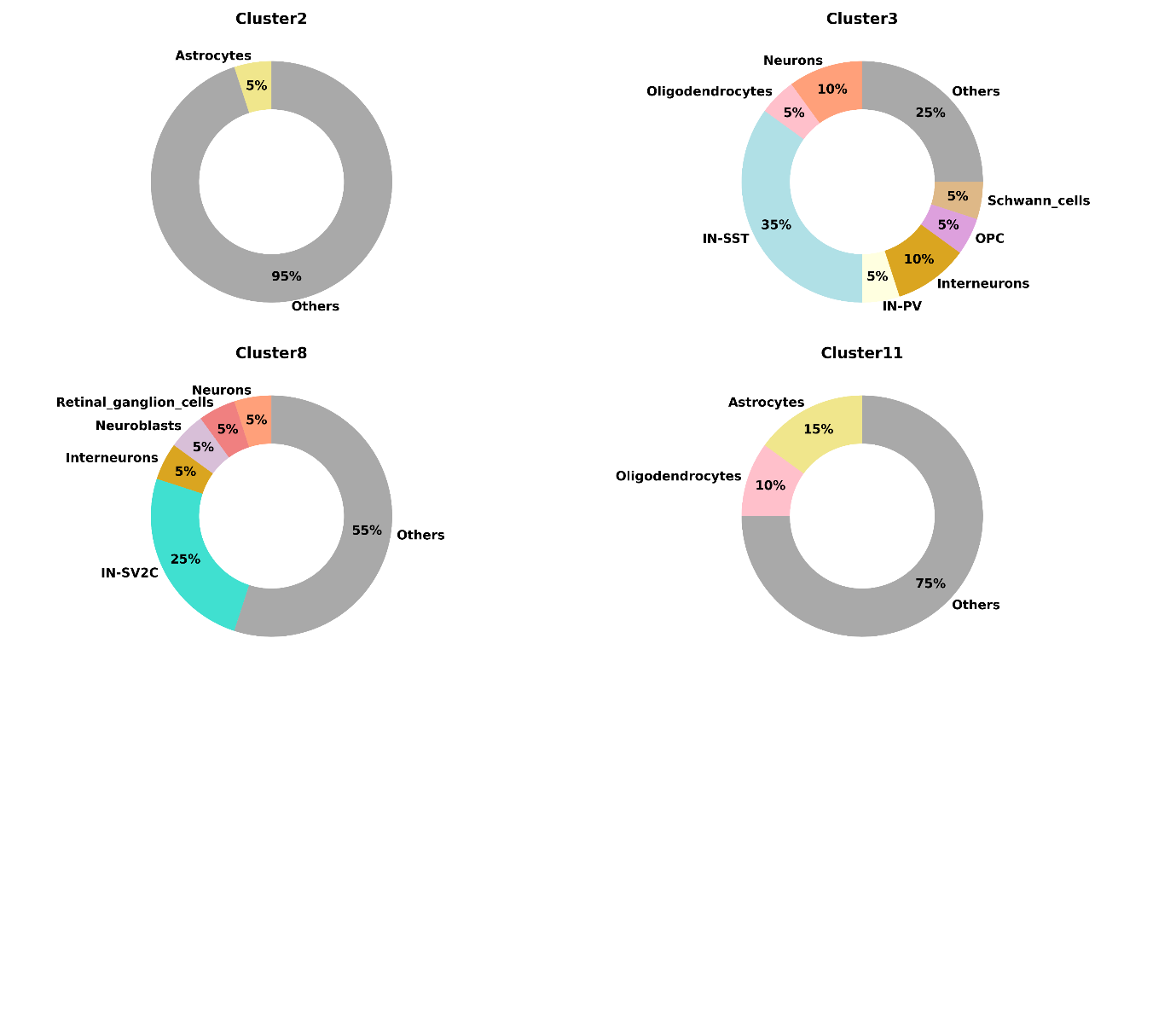

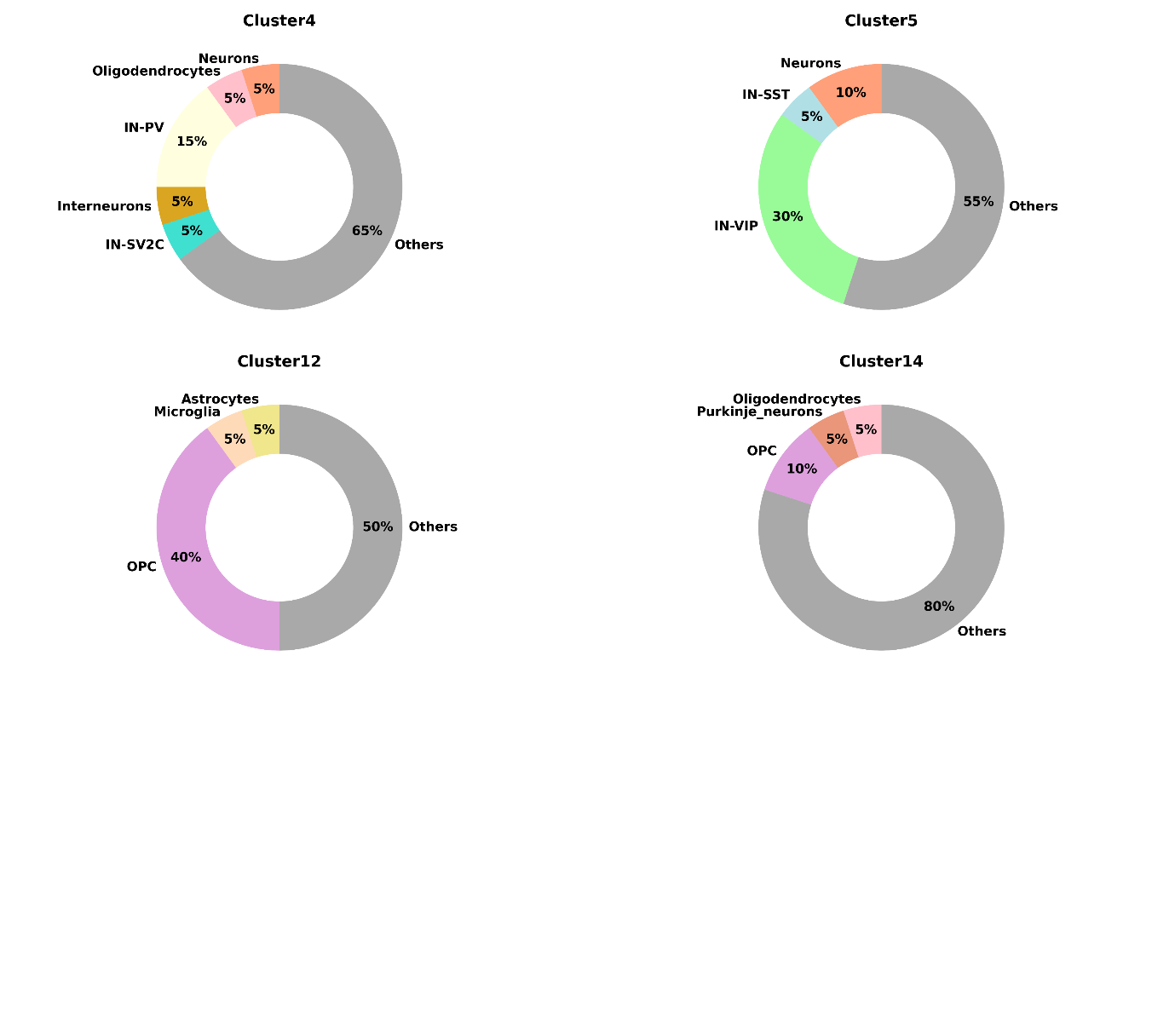


**MTG**
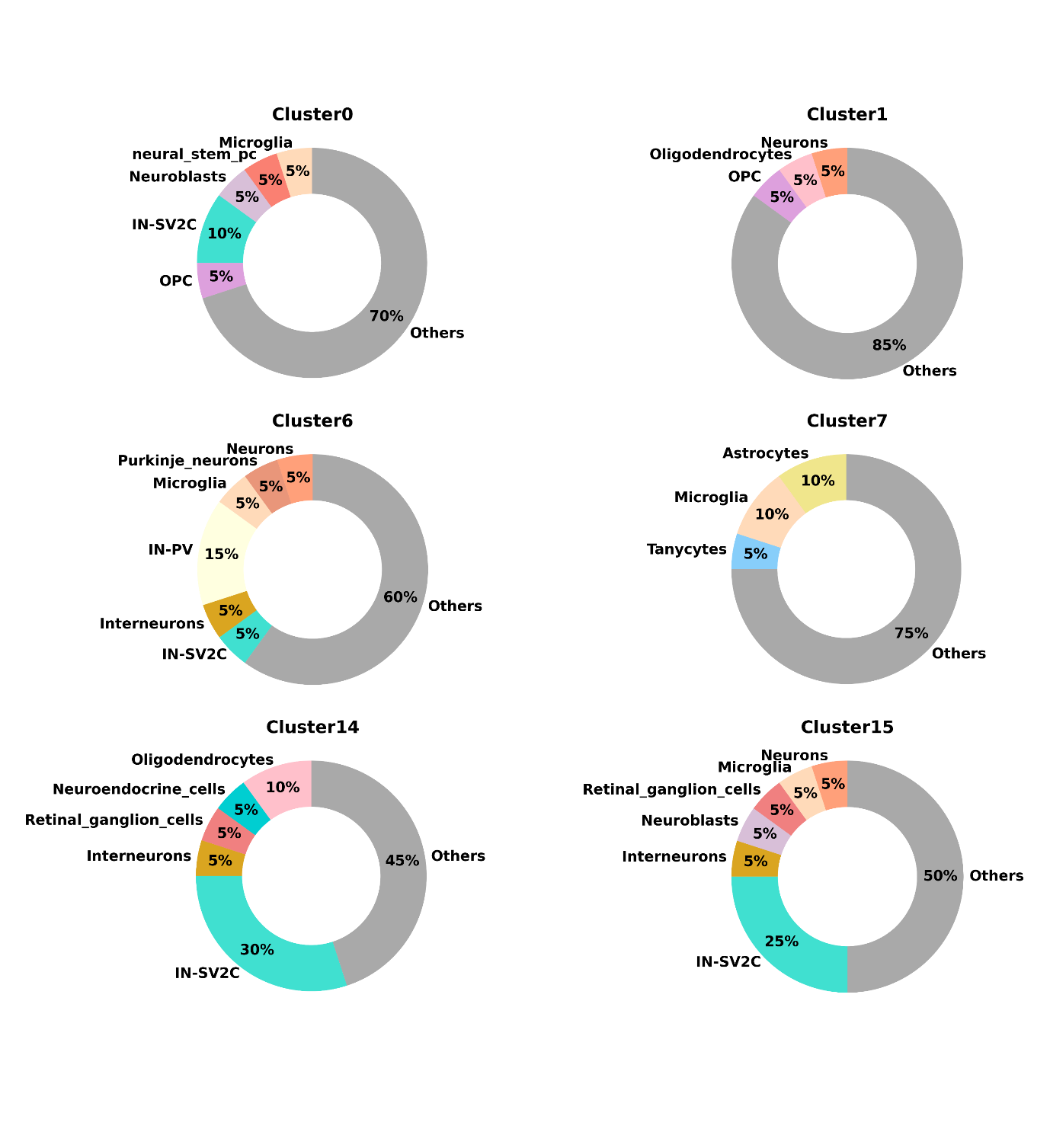

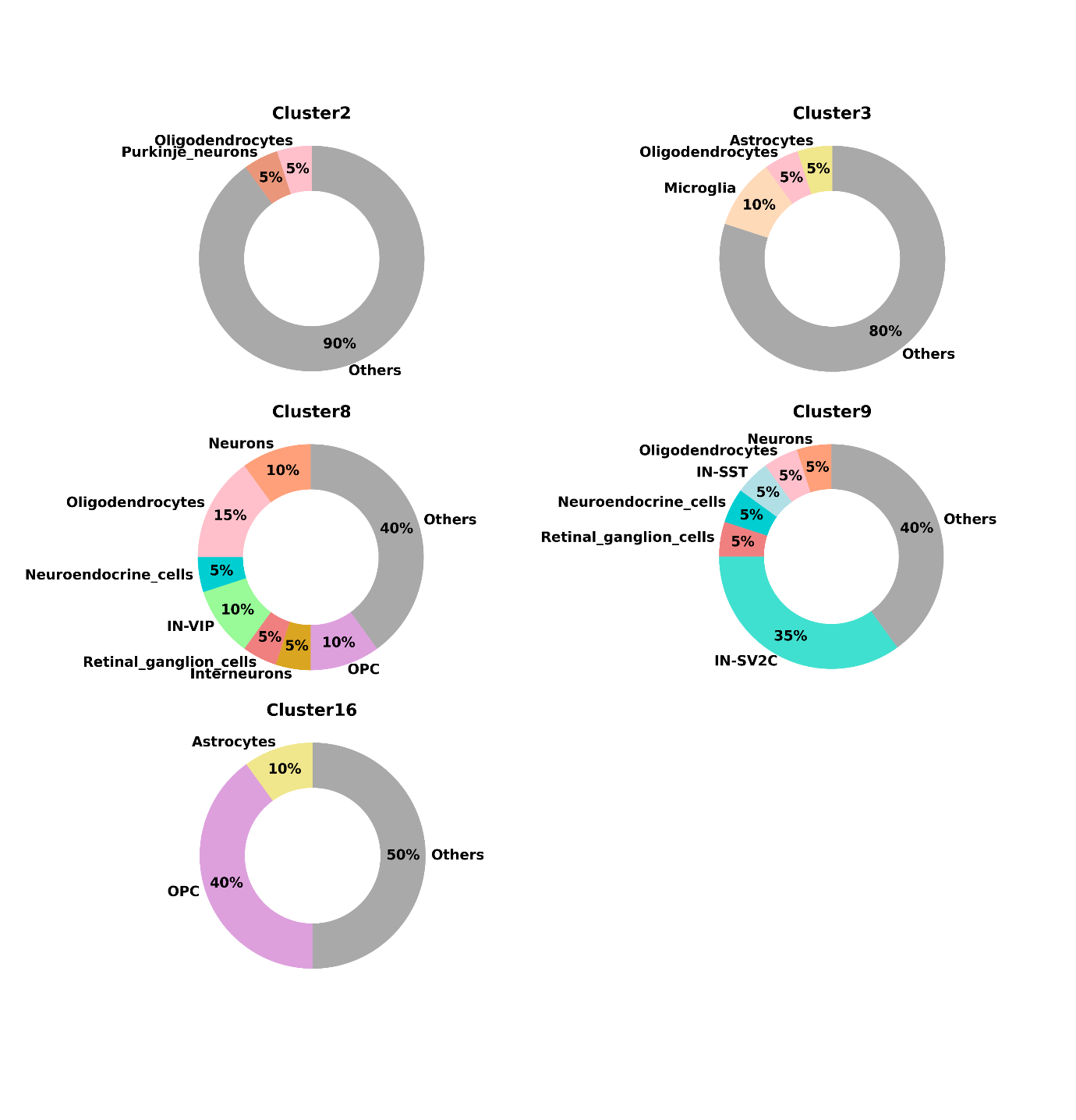

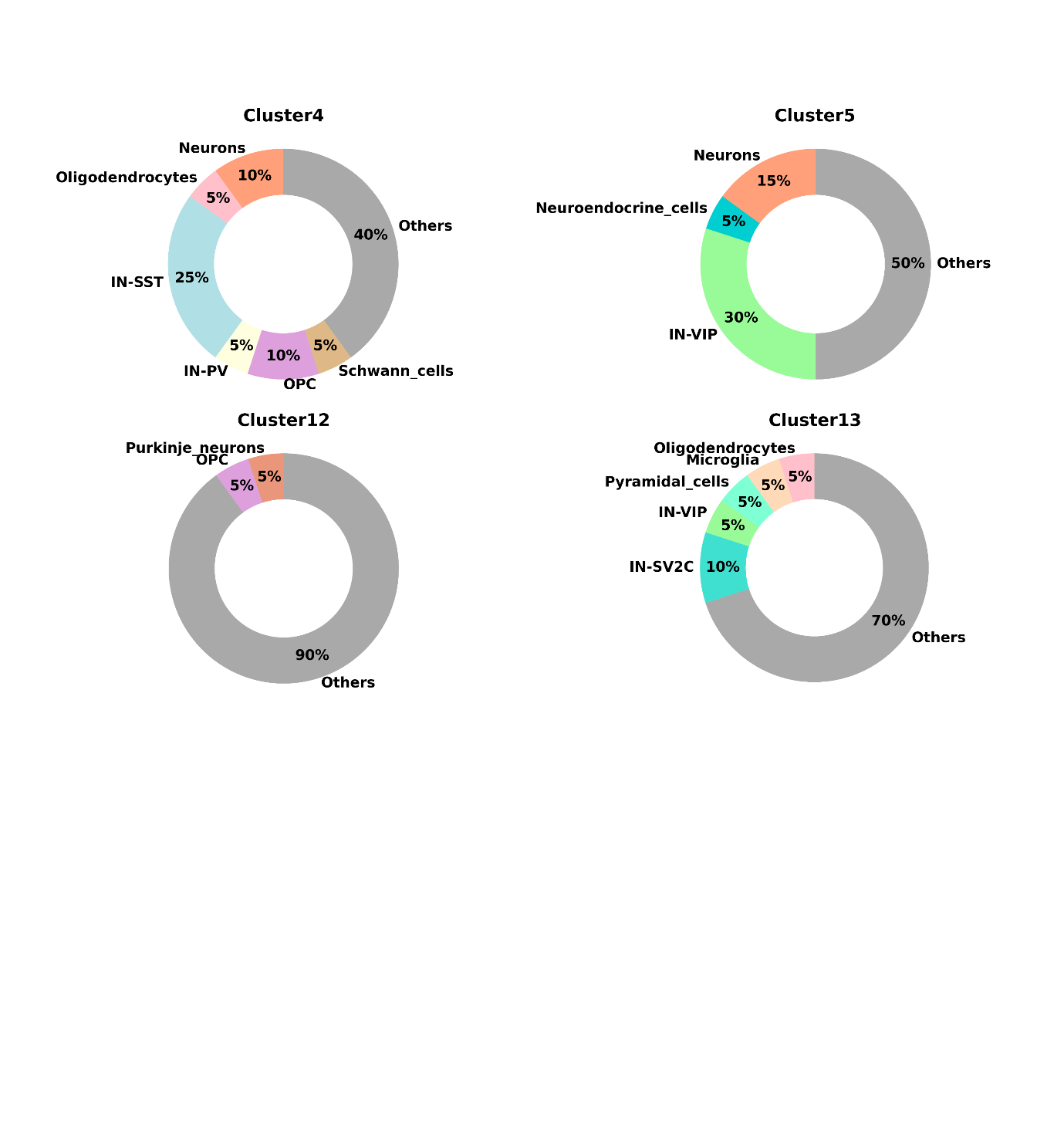


**VISP**
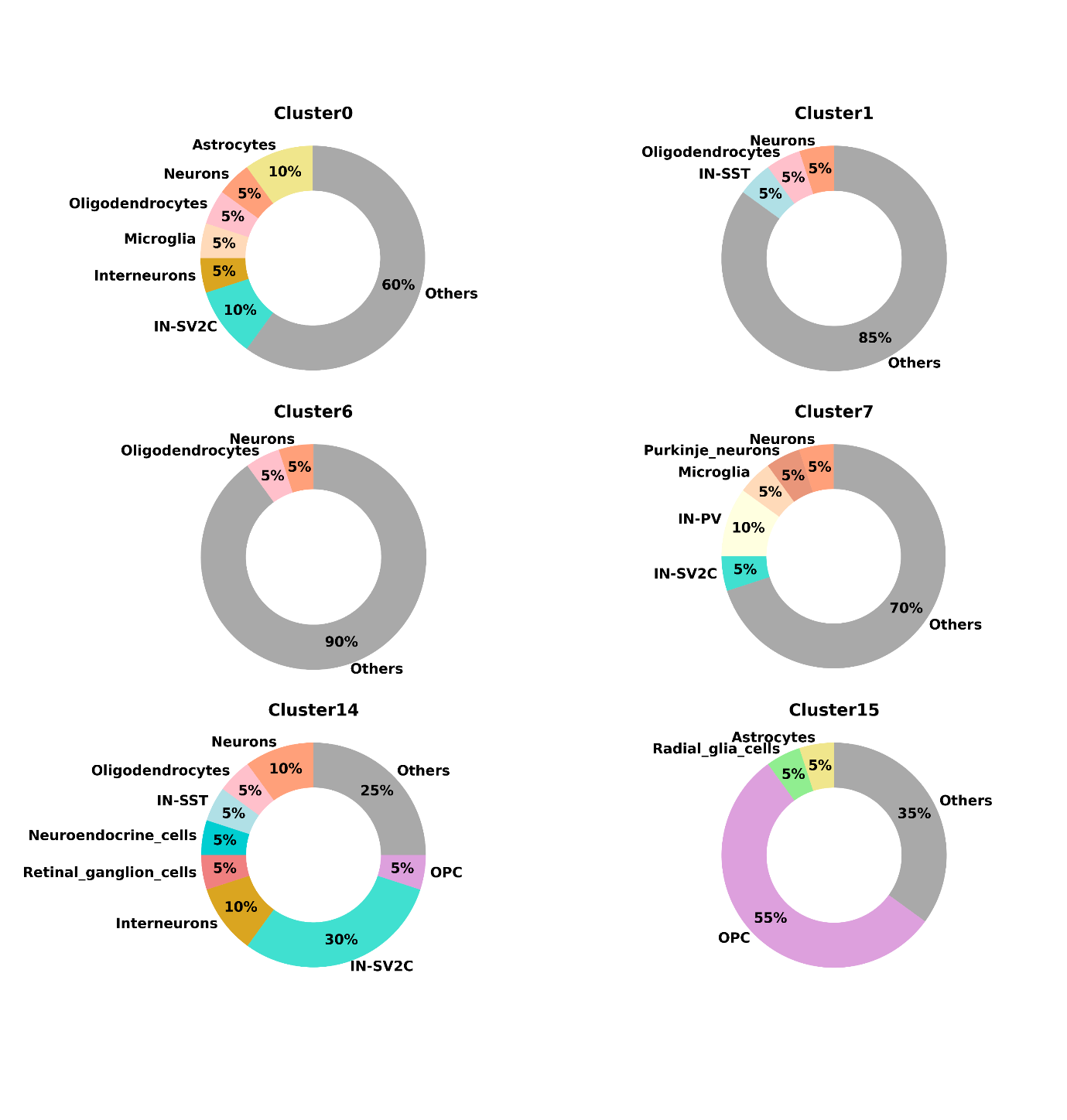

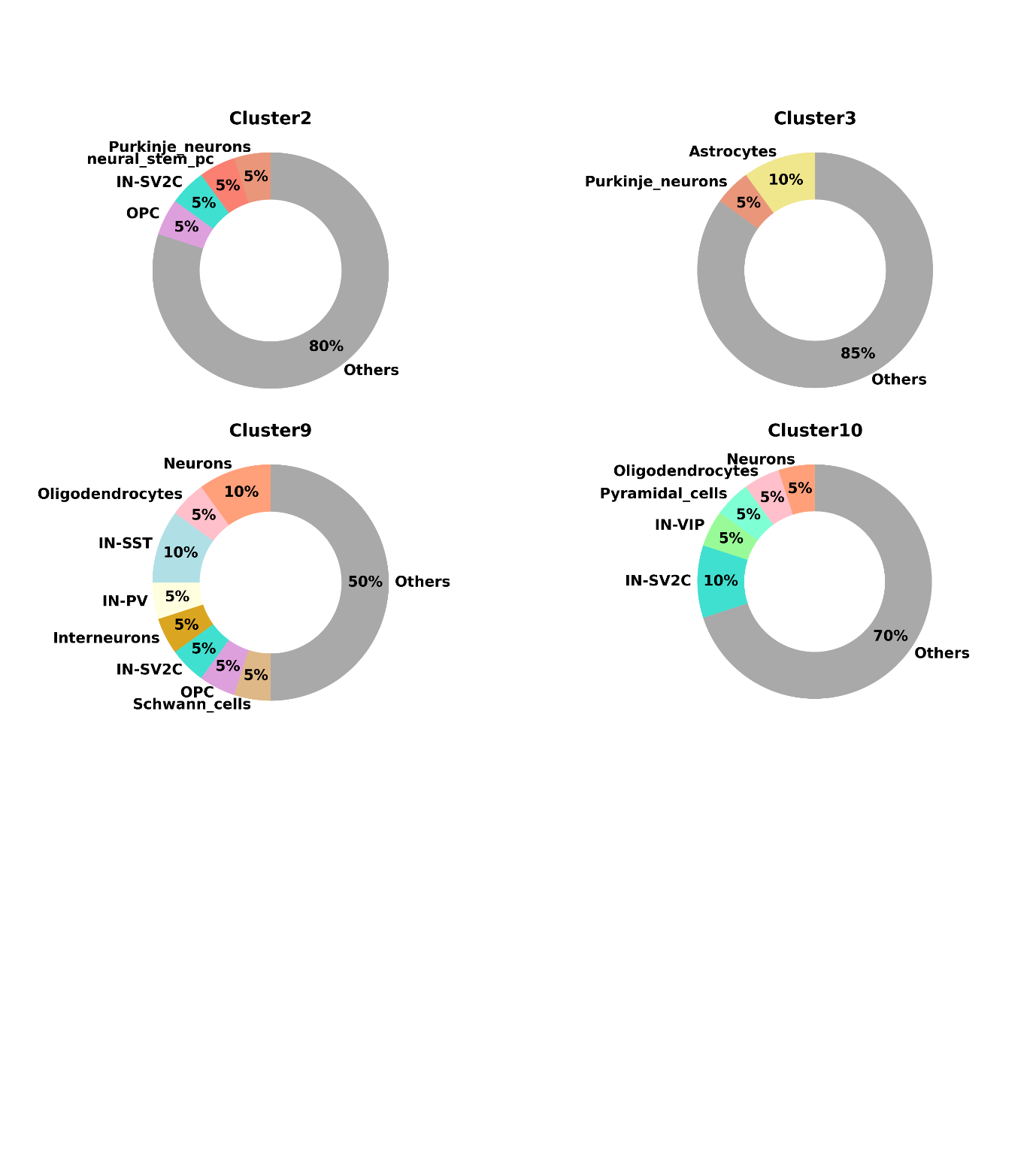

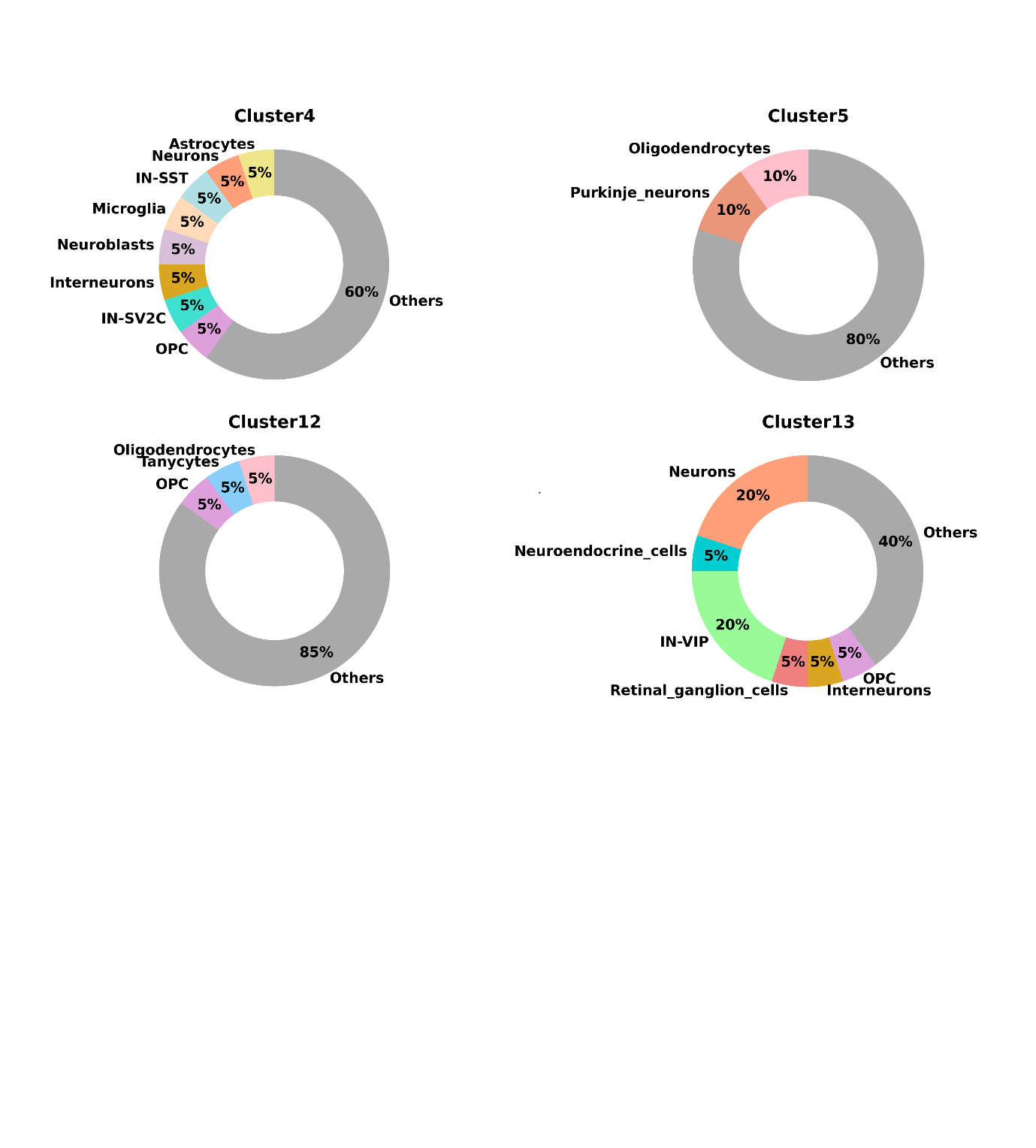


**Supplementary Figure. 10| Donut plot depicting composition of Top 20 DE in cluster genes.** Donut plot displaying the composition of top 20 DE genes in comparison to known marker gene list (yellow-astrocytes, orange-microglia, pink-oligodendrocytes, grey-others).

**ACC**


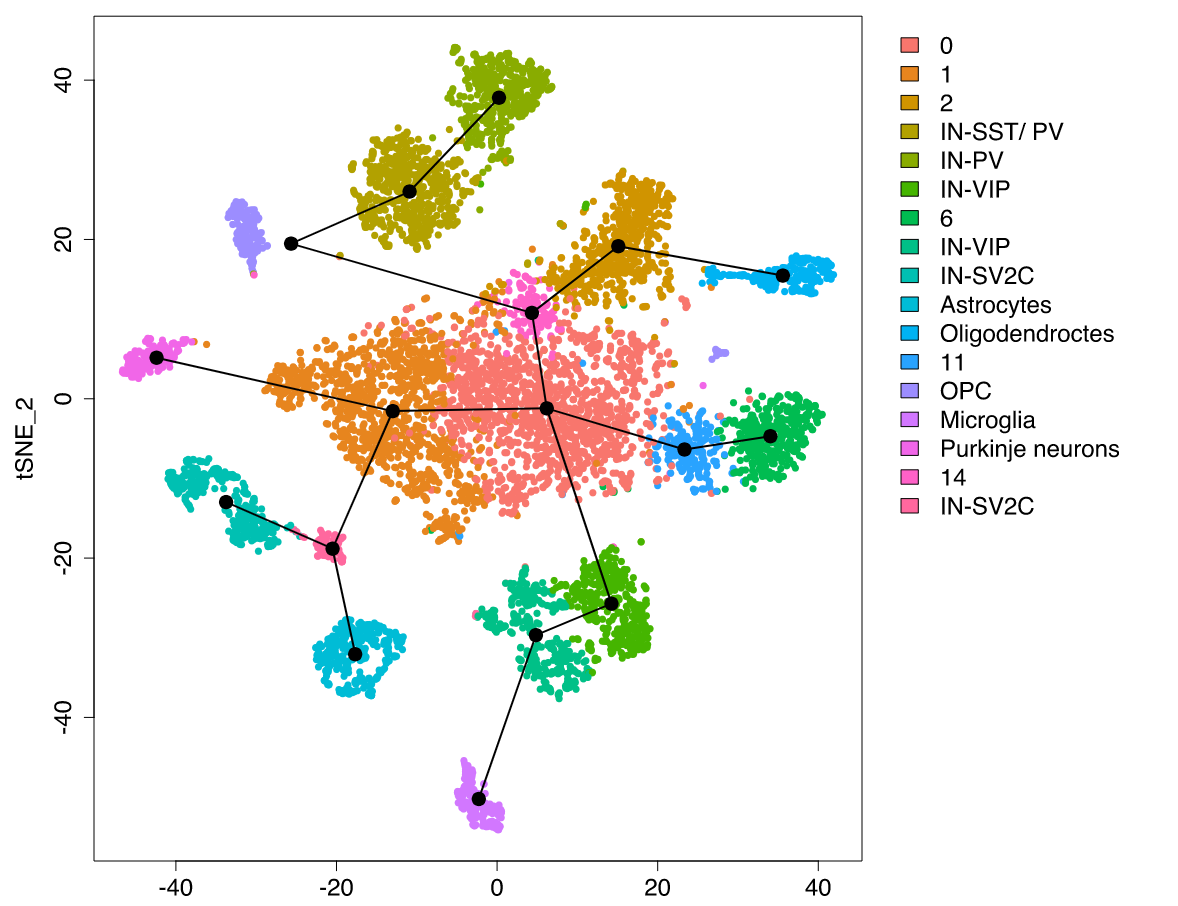


**MTG**


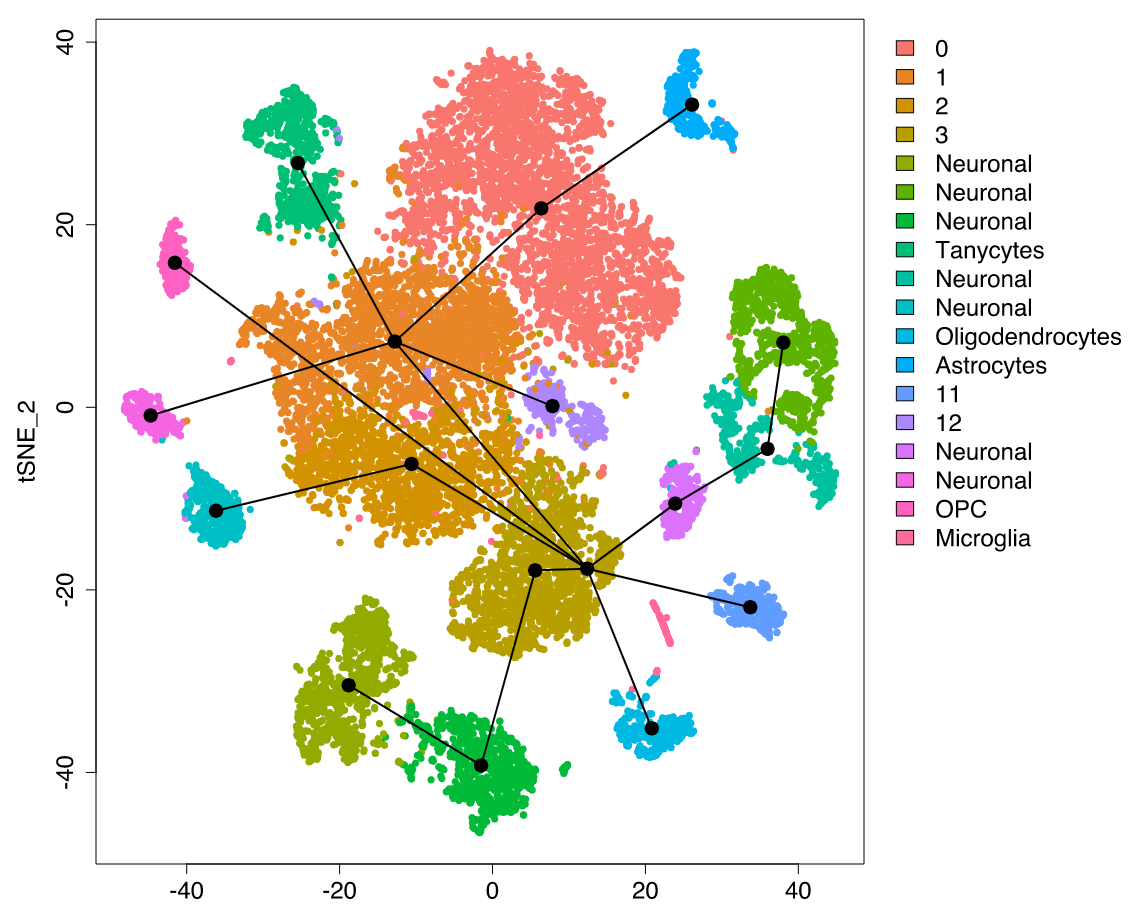


**VISP**


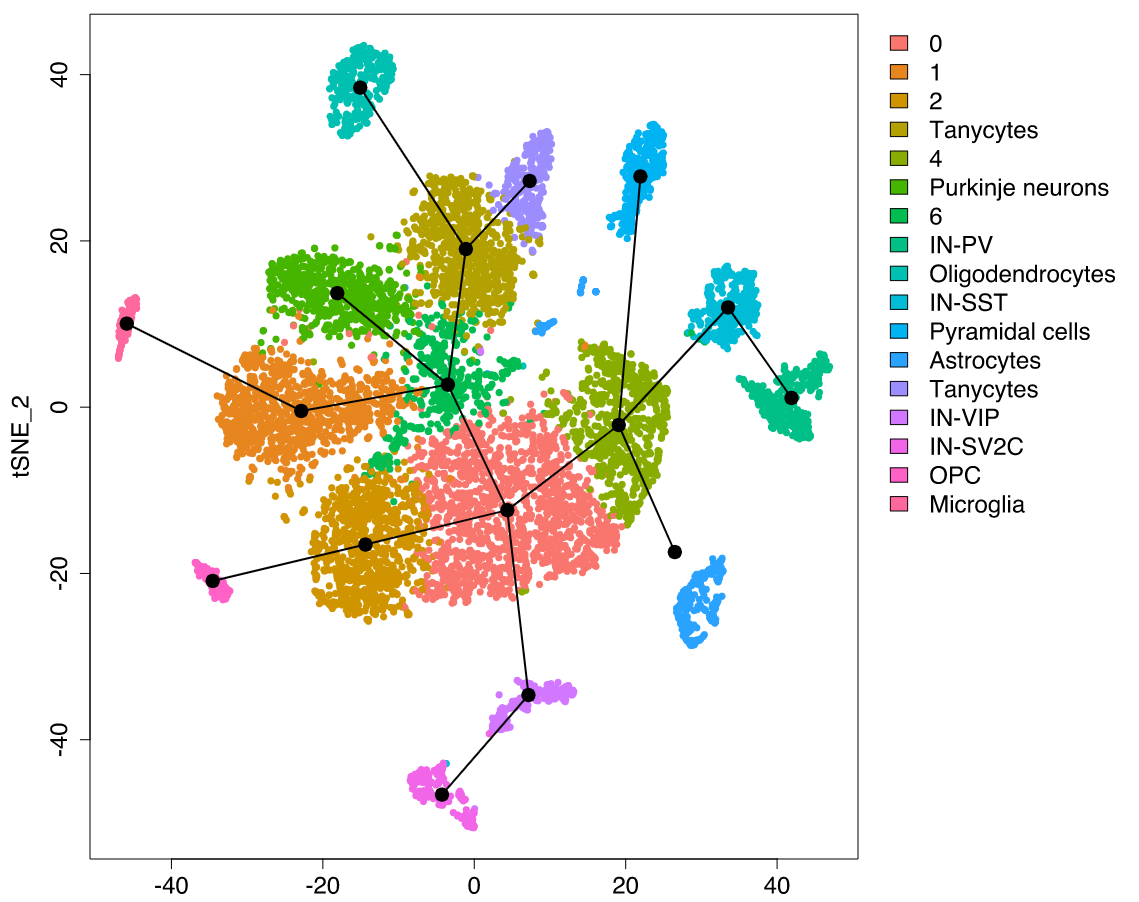


**Supplementary Figure. 11| Lineage analysis of clusters in ACC, MTG and VISp region.** Trajectory analysis performed to find the lineage association of clusters done using Slingshot.


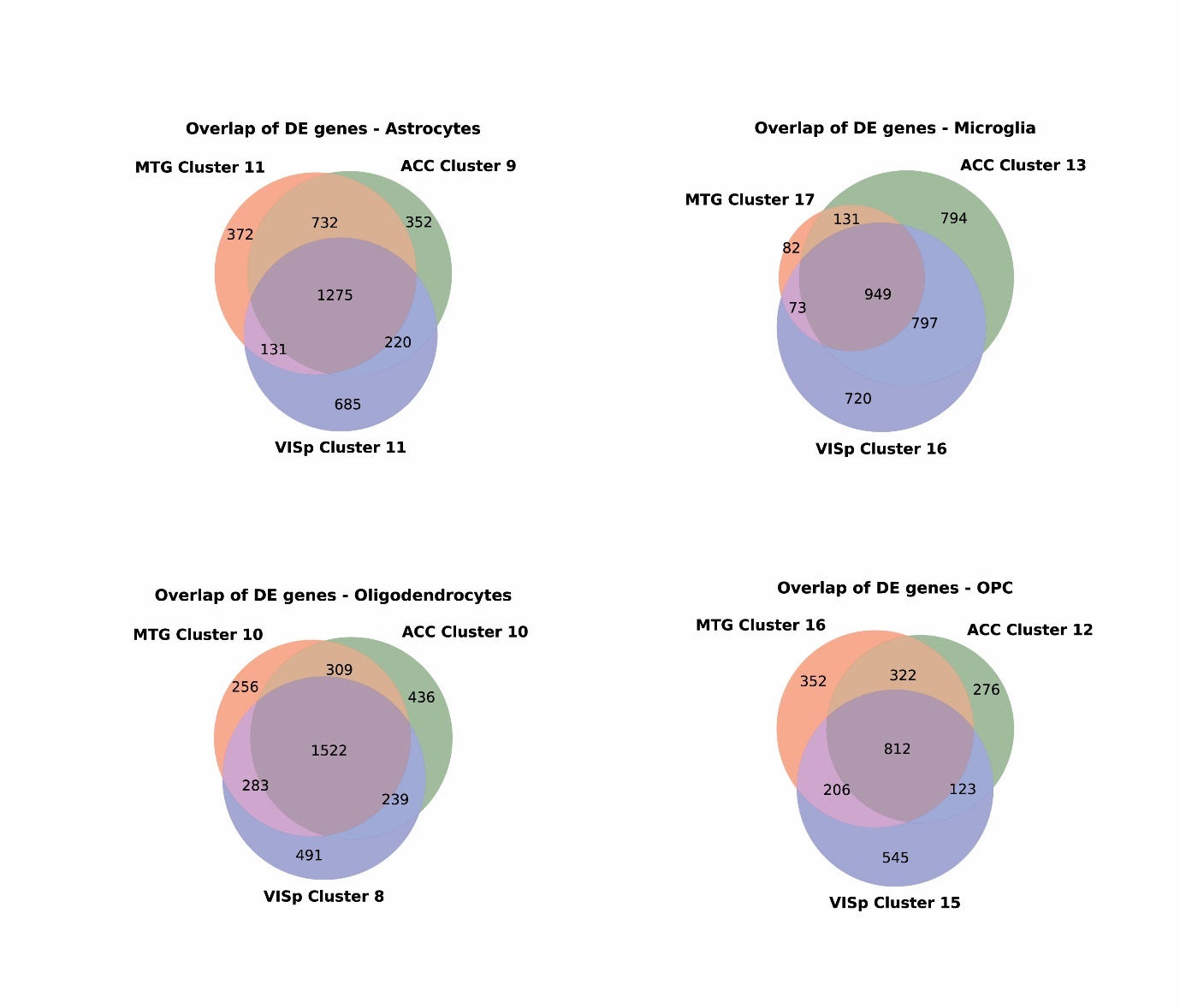


**Supplementary Figure. 12| Venn diagram depicting the overlap between genes.** Overlap of DE genes in astrocytes, microglia, oligodendrocytes and OPC among clusters across ACC, MTG and VISp brain regions.


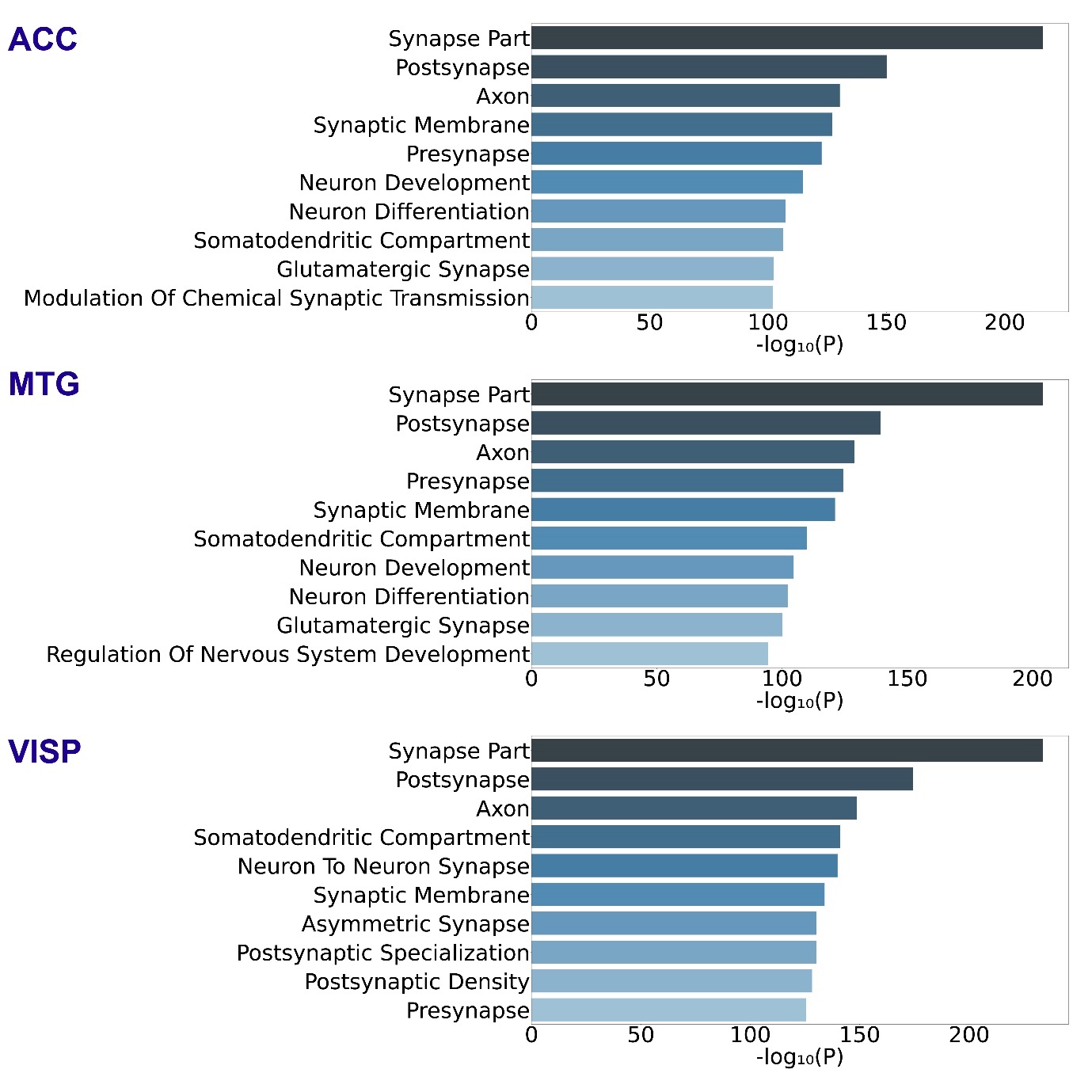


**Supplementary Figure. 13| Top biological pathways enriched for cluster genes.** x axis shows biological pathways, and the y axis shows statistical significance as −log10 (P) of z statistics.


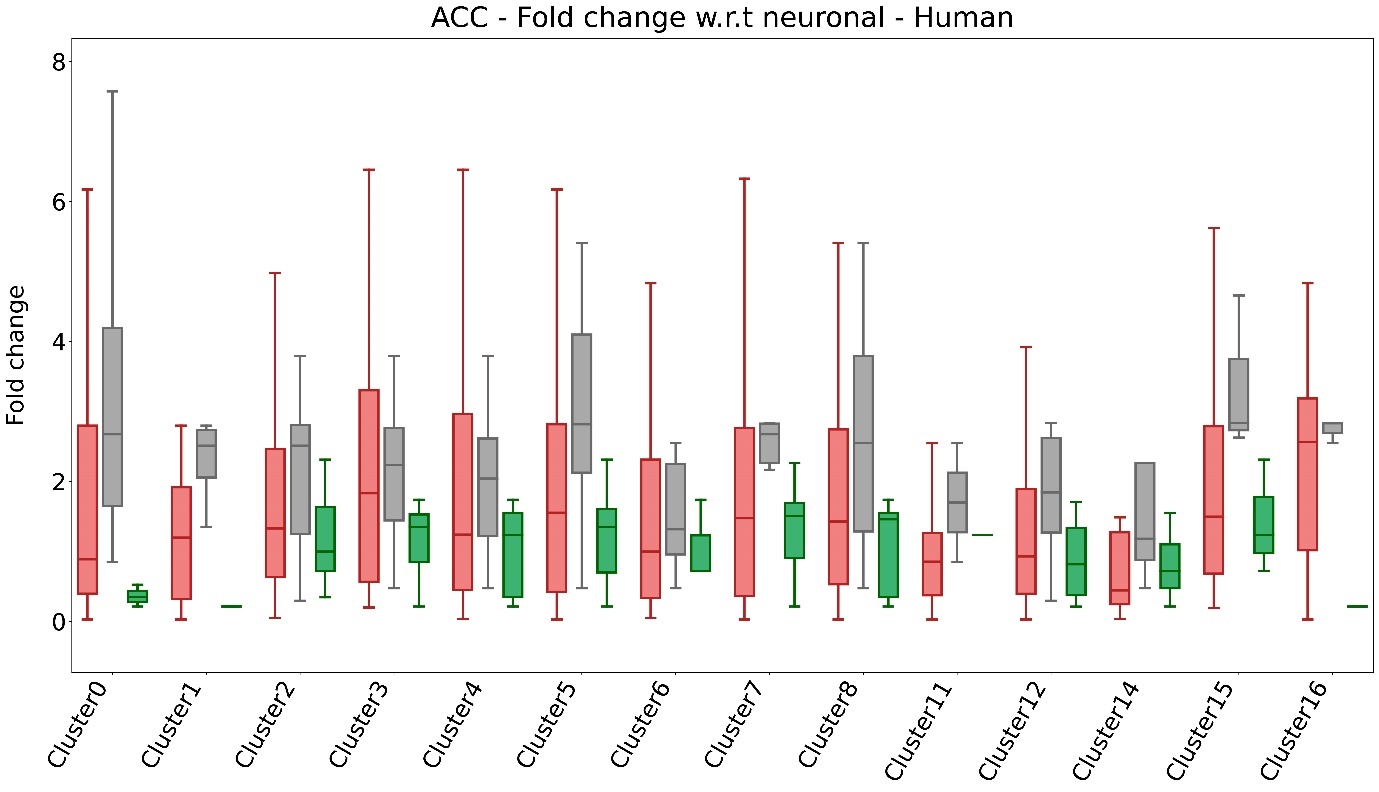


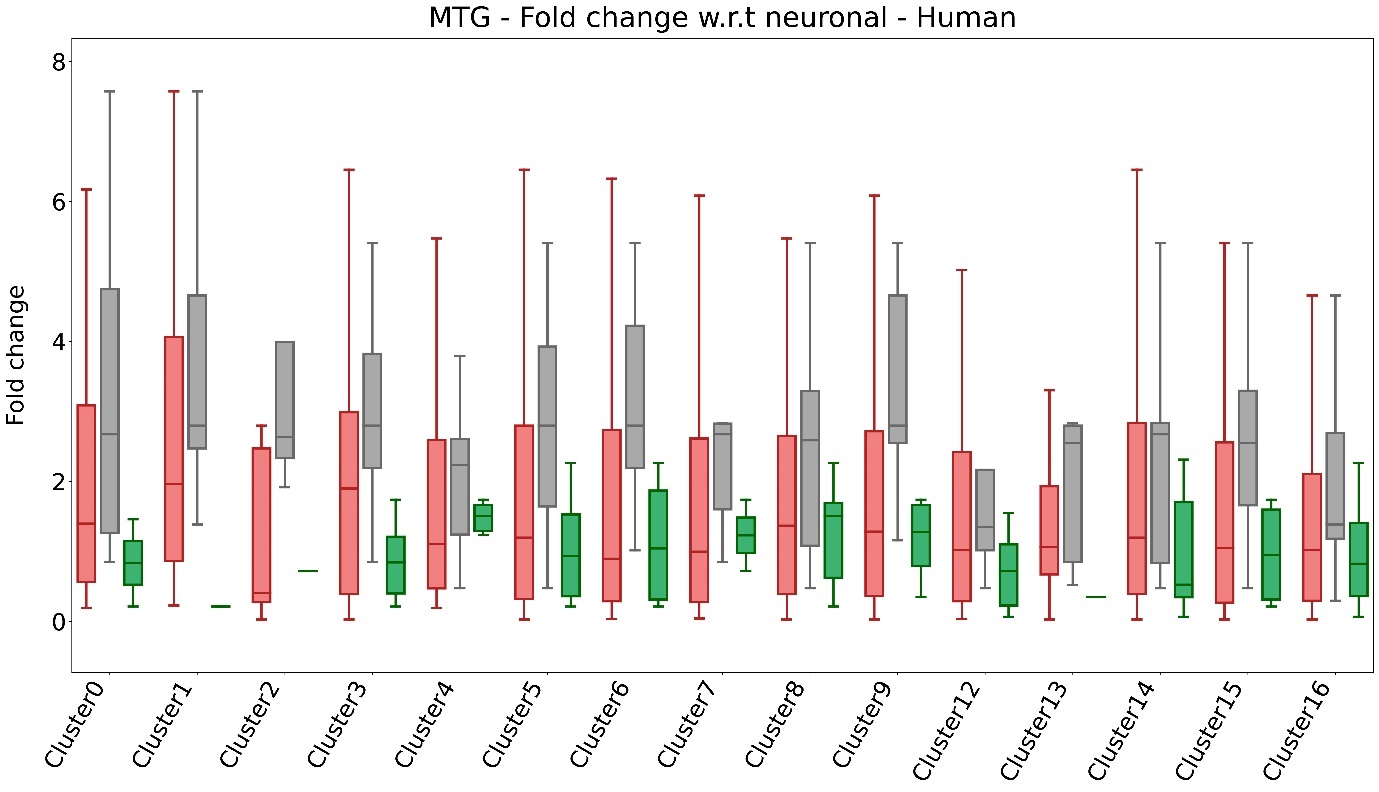


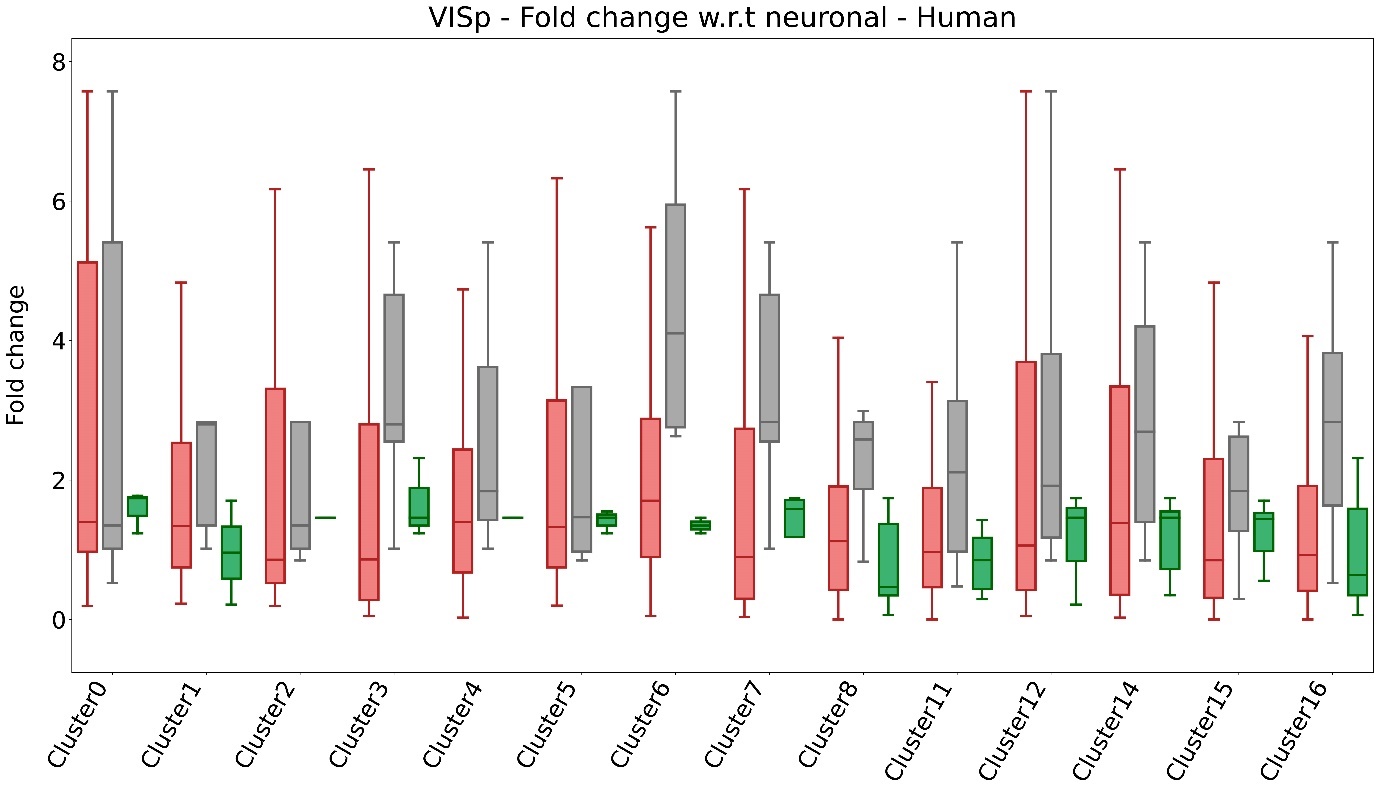


**Supplementary Figure. 14| Fold change of cluster genes intersected with LOF genes.** Fold change of cluster genes wrt neurons - non neuronal ᴒ cluster genes ᴒ LOF (red), non-neuronal ᴒ cluster genes ᴒ LOF ᴒ known markers (grey), non-neuronal ᴒ cluster genes ᴒ housekeeping genes (green).


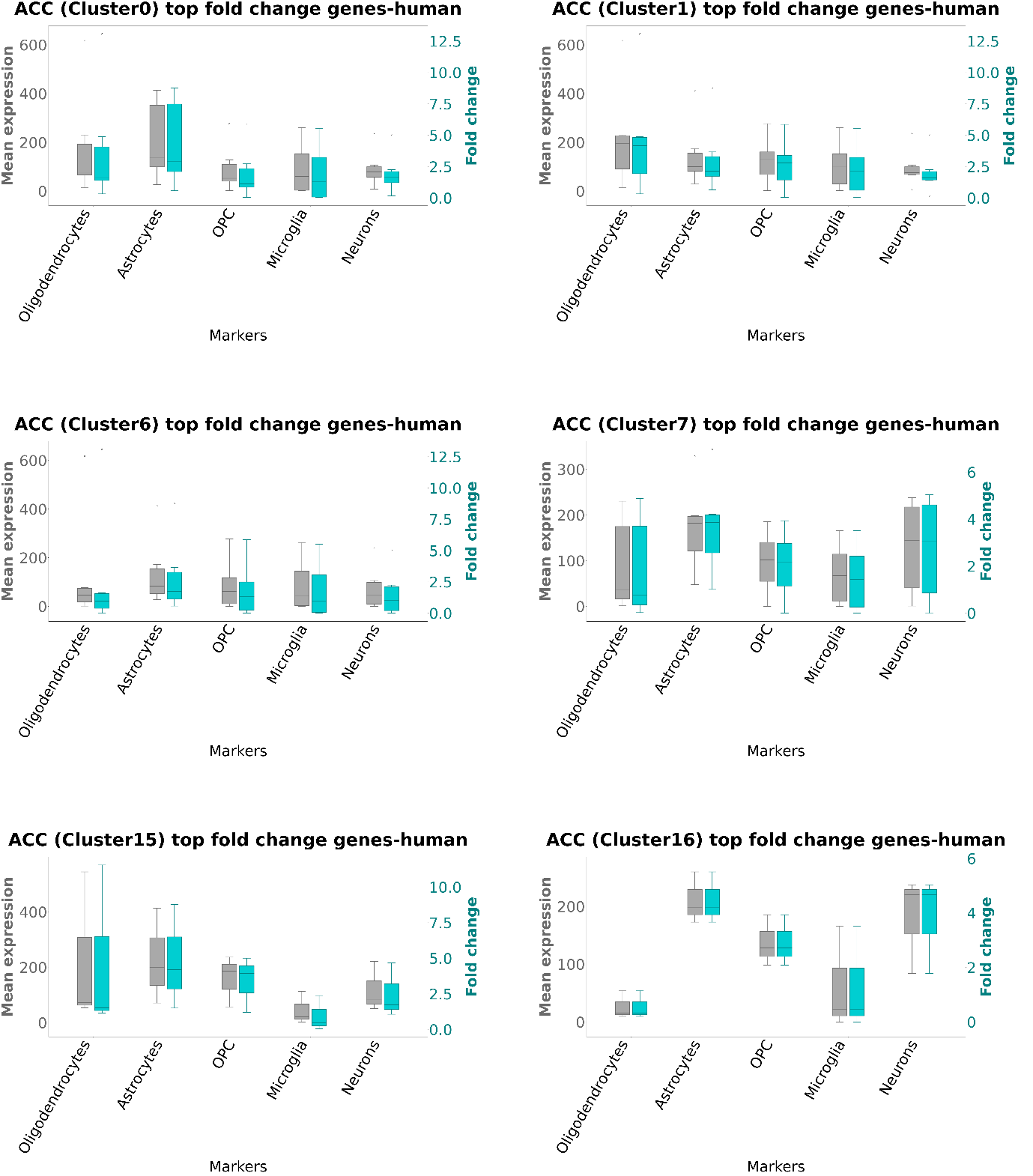

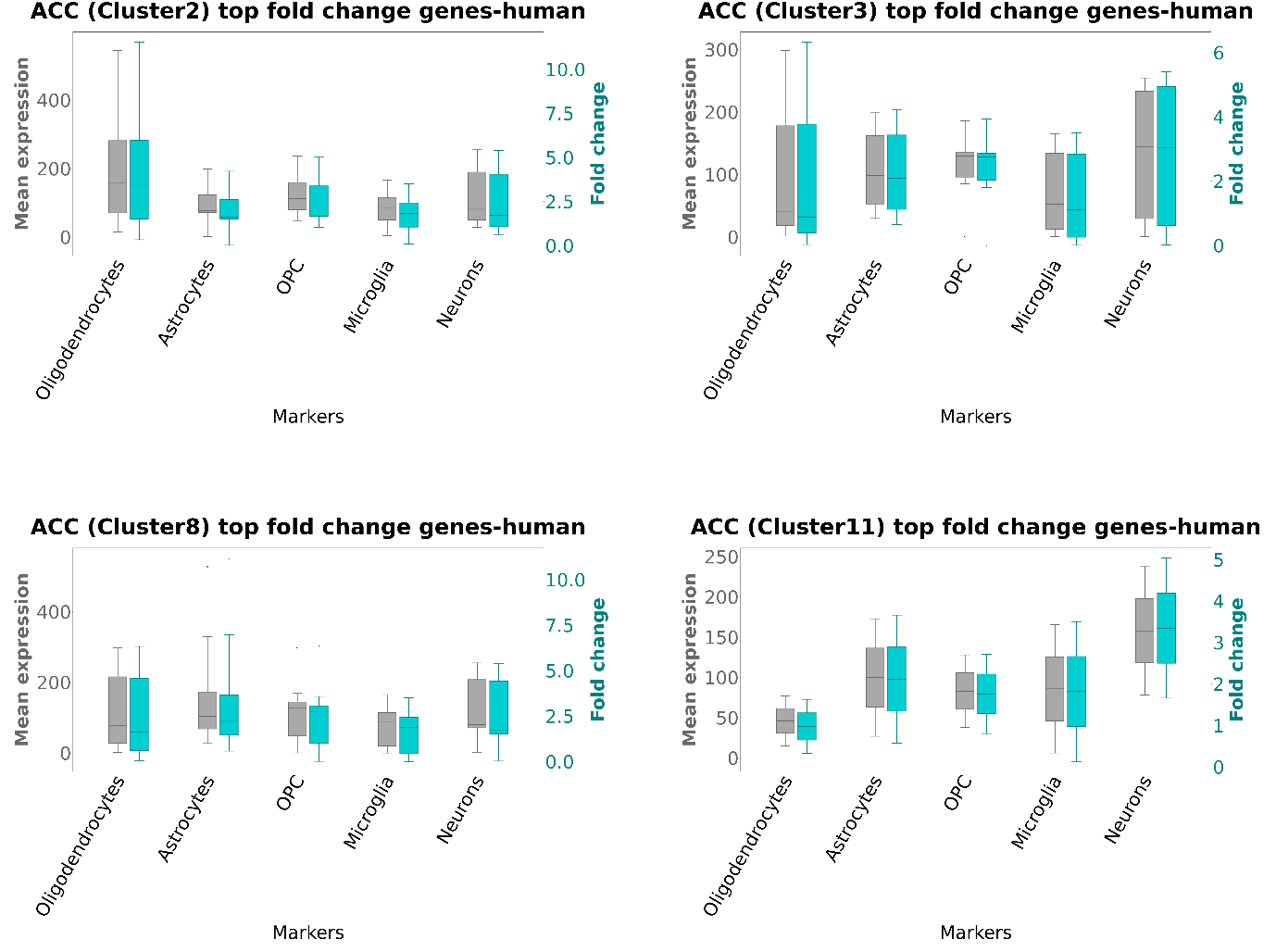

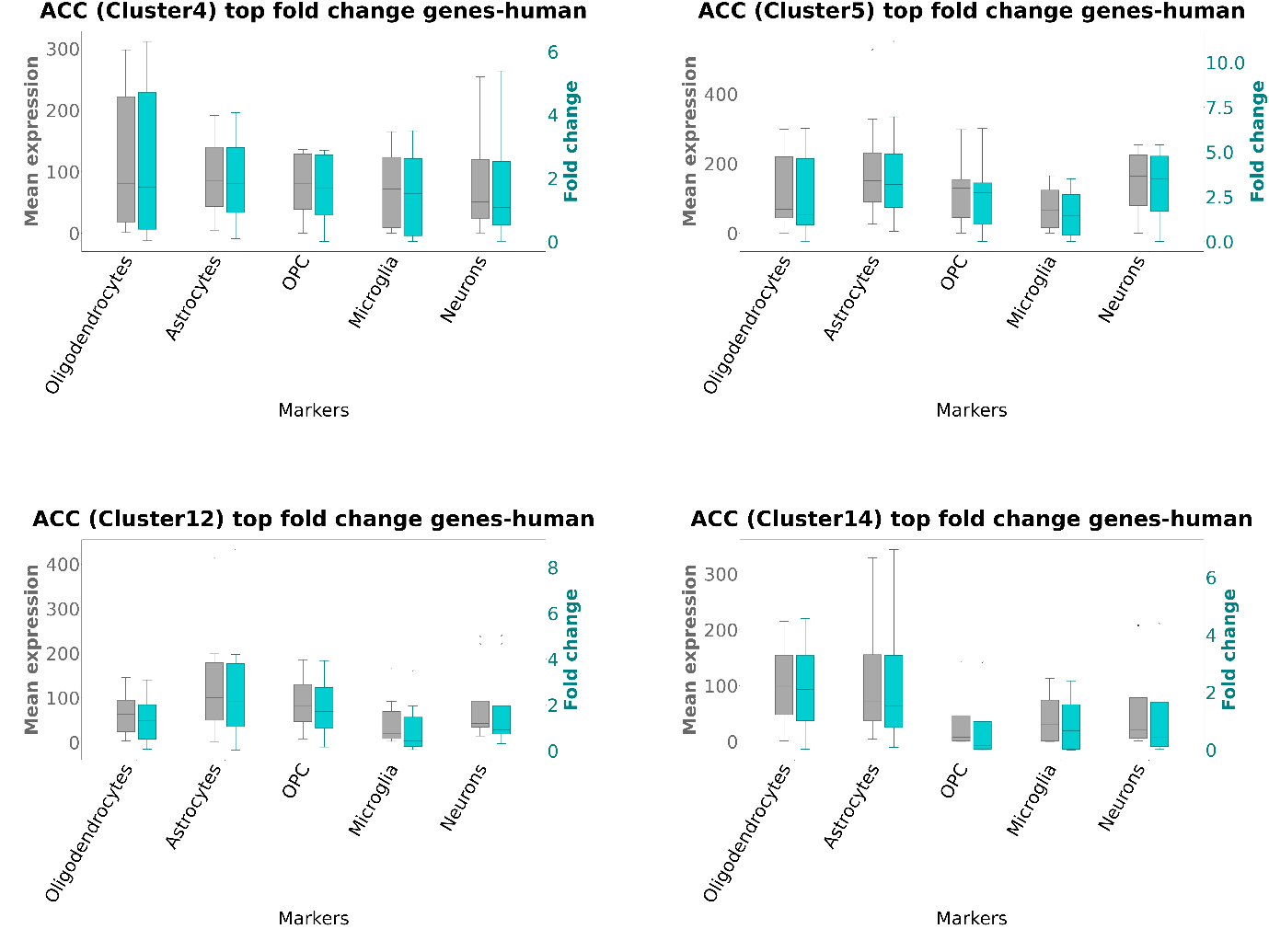

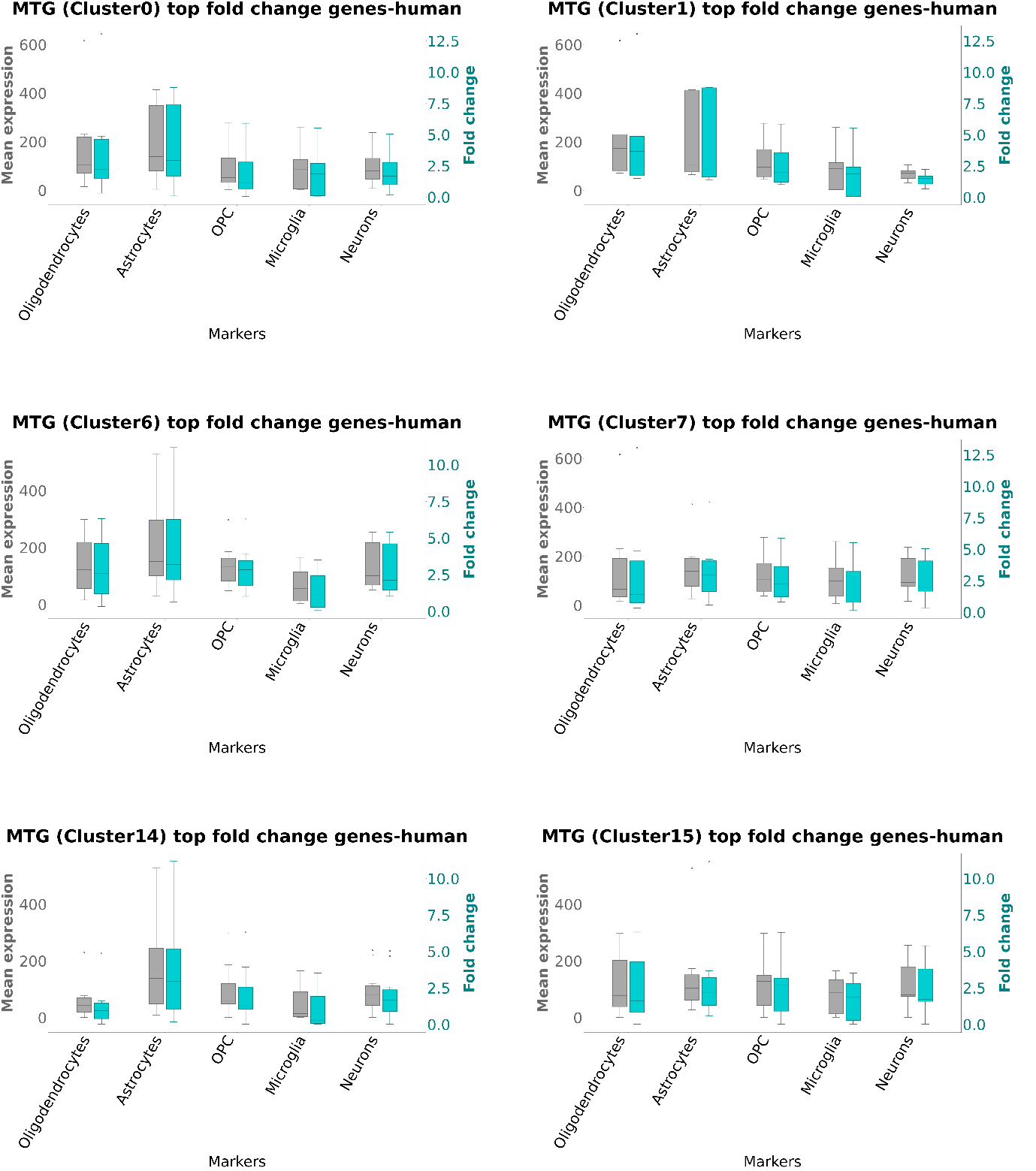

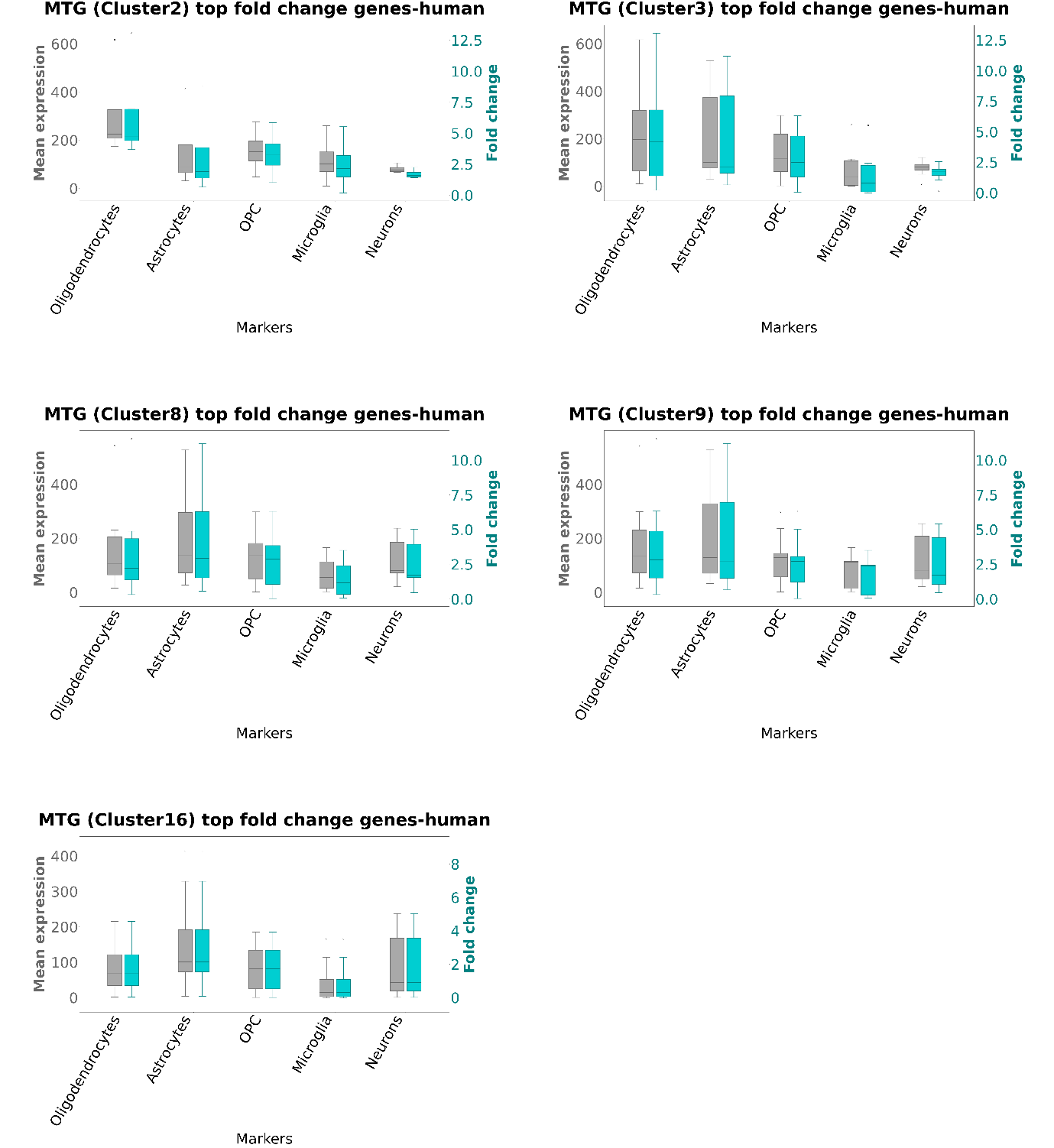

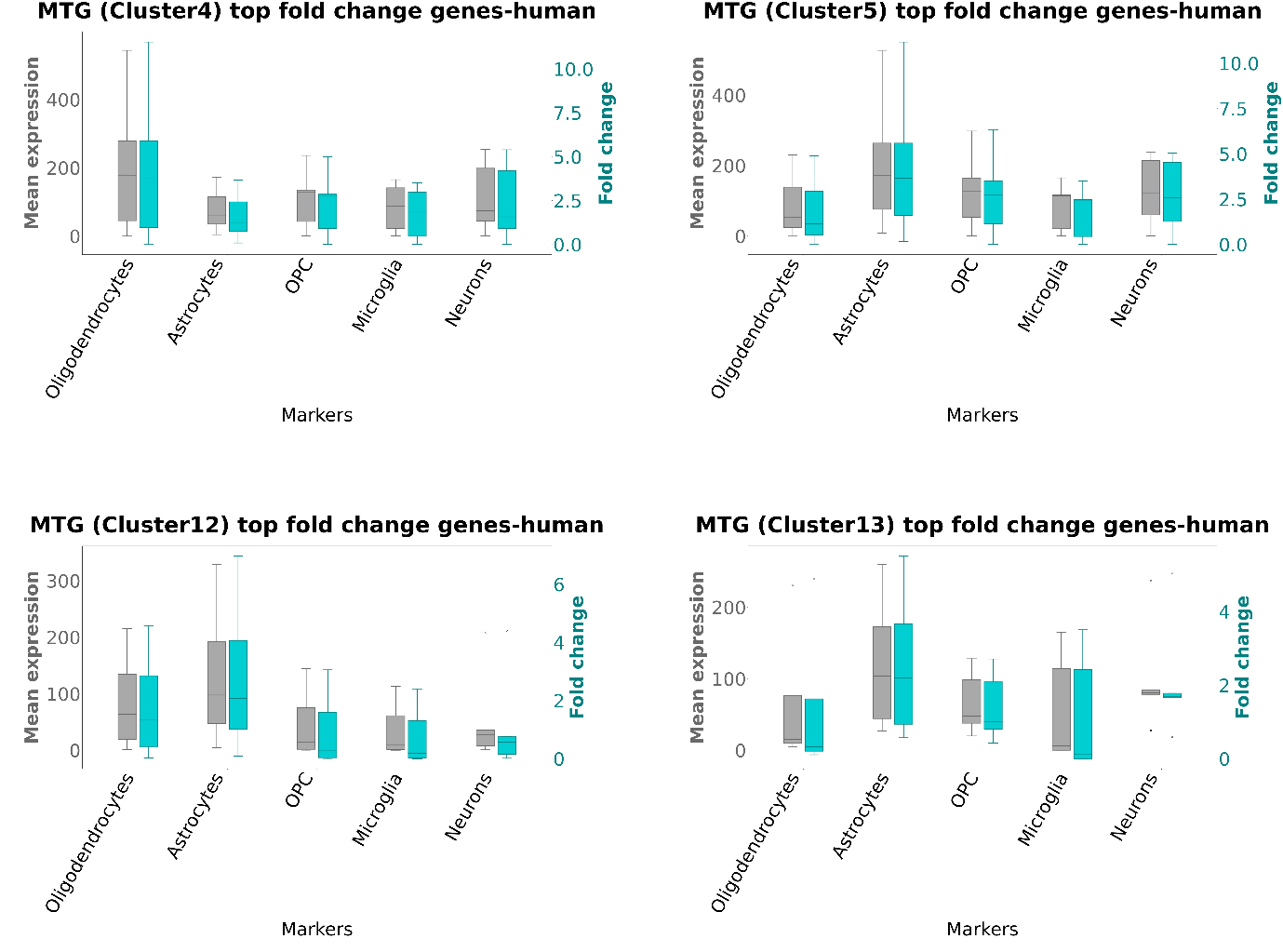

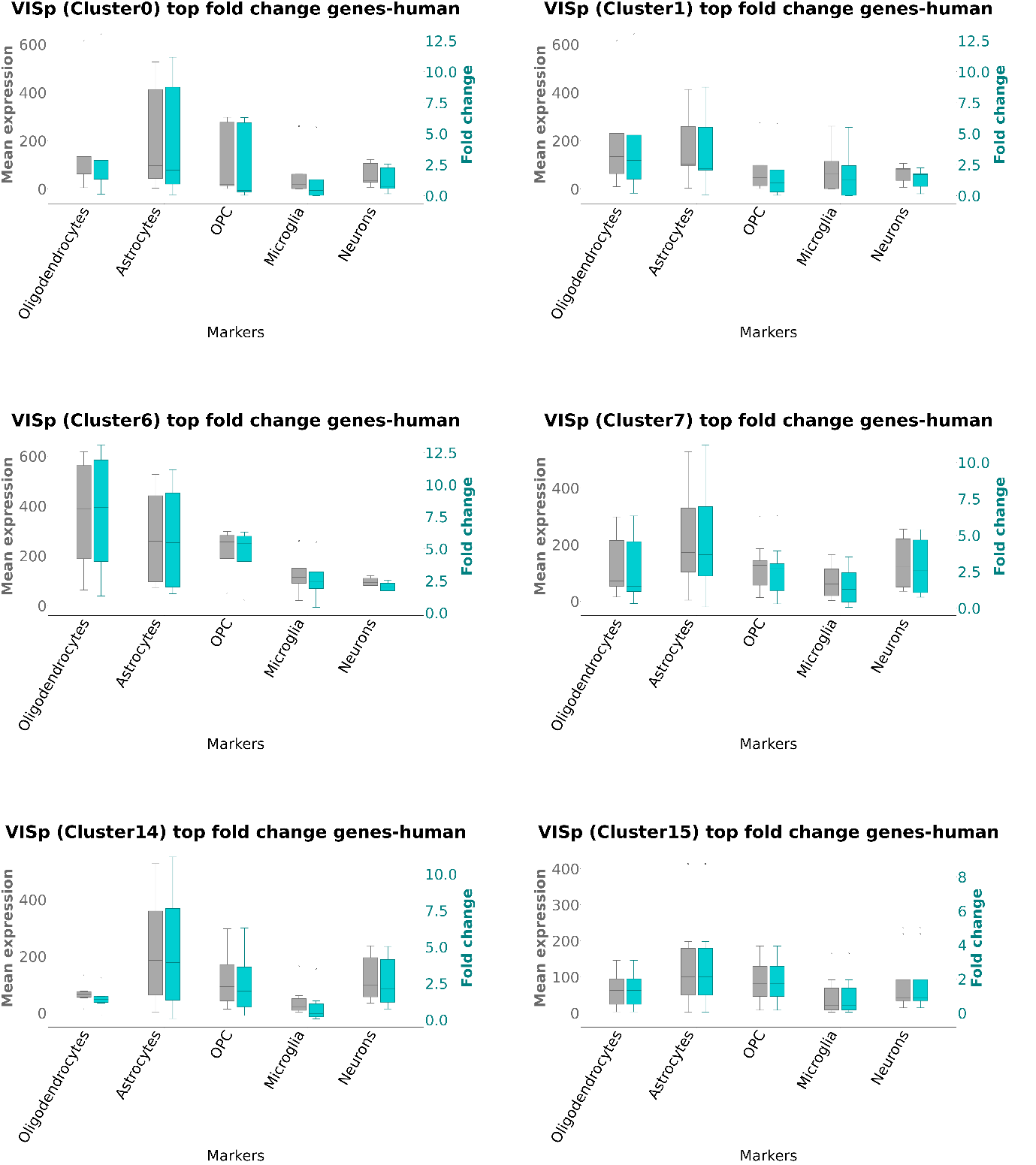

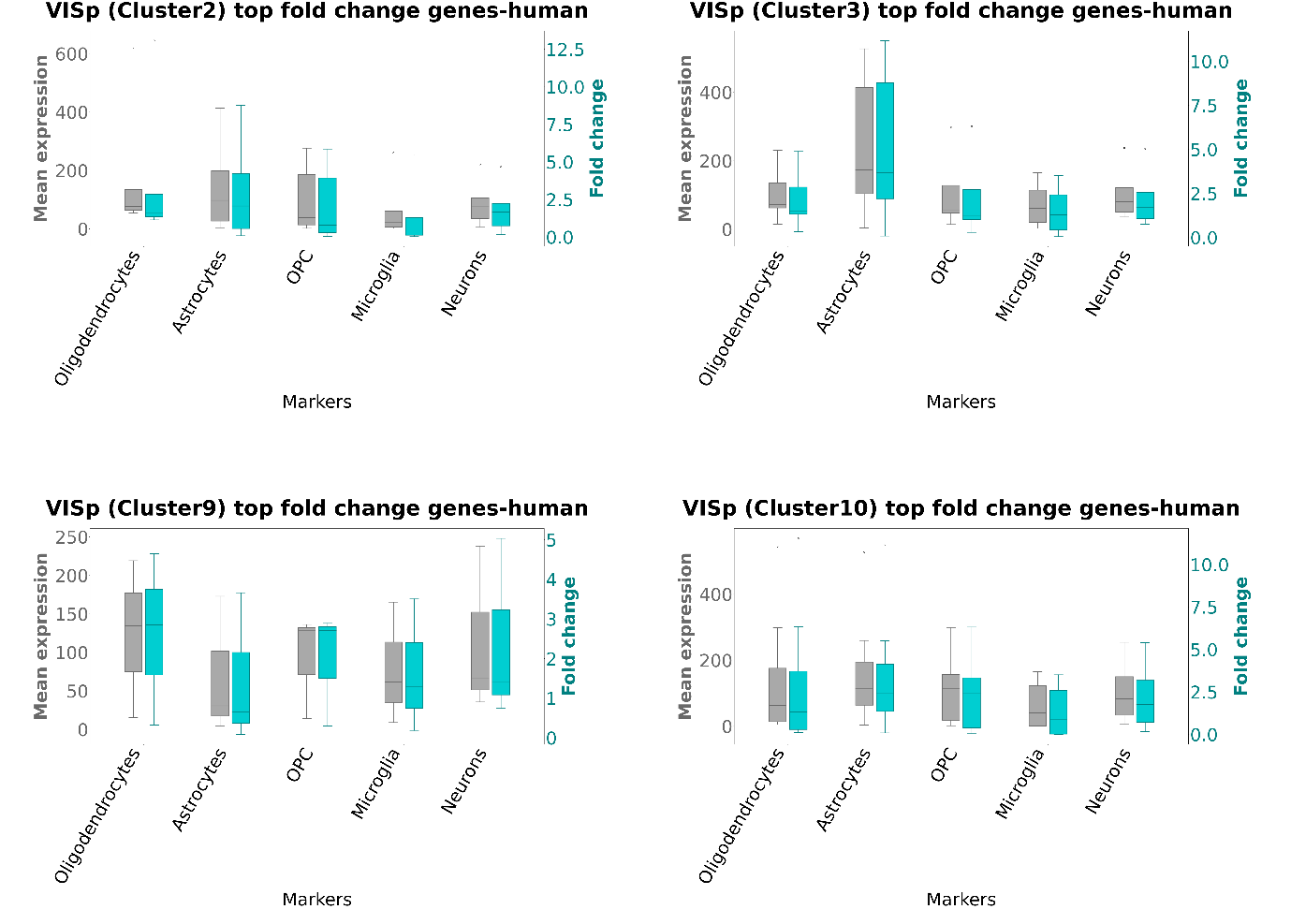

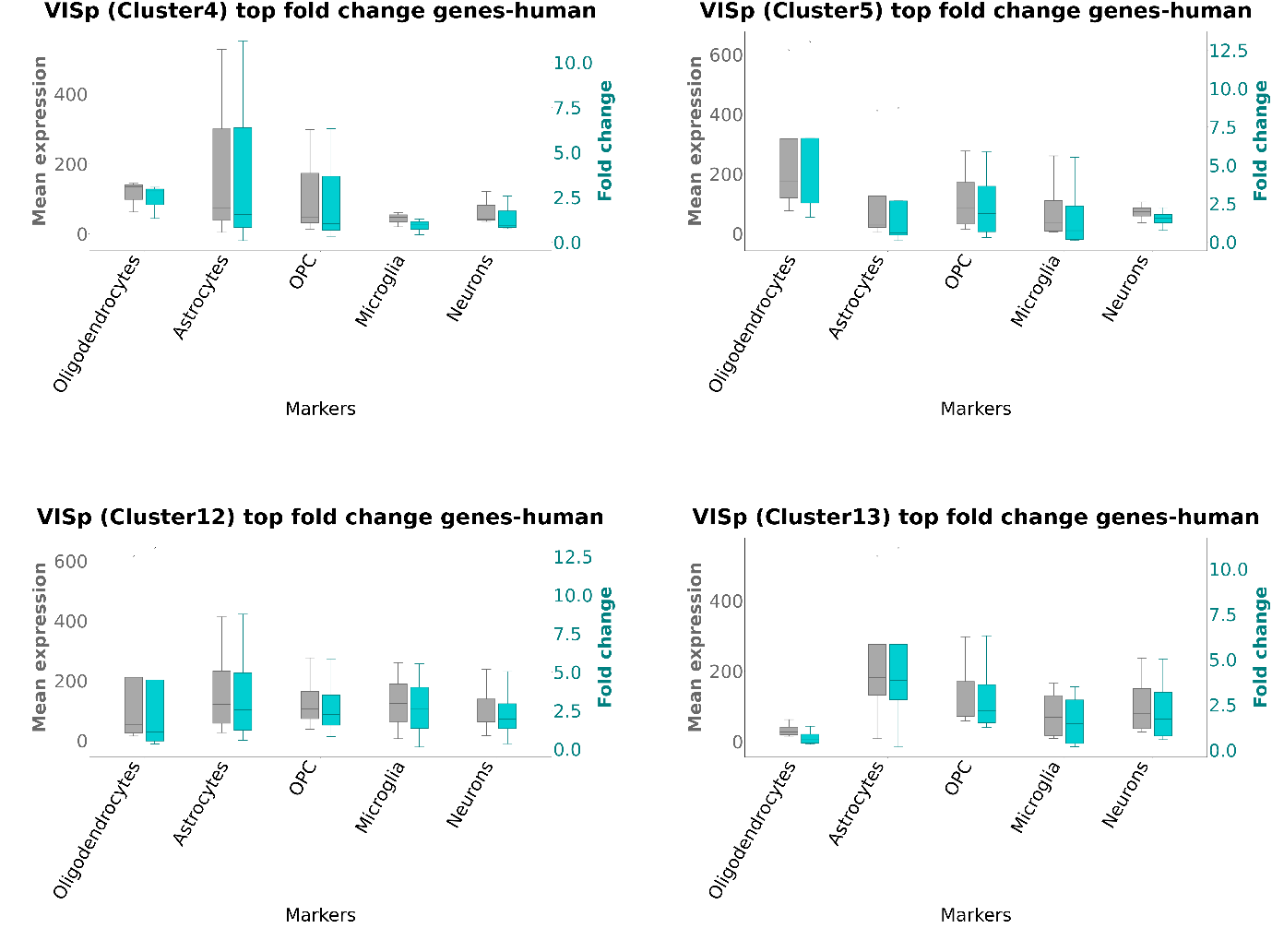


**Supplementary Figure. 15| Mean expression and fold change of top fold change genes in clusters across ACC, MTG, VISP regions in GSE67835 (human).** Fold change (green) calculated wrt neurons across oligodendrocytes, astrocytes, OPC, microglia and neurons.


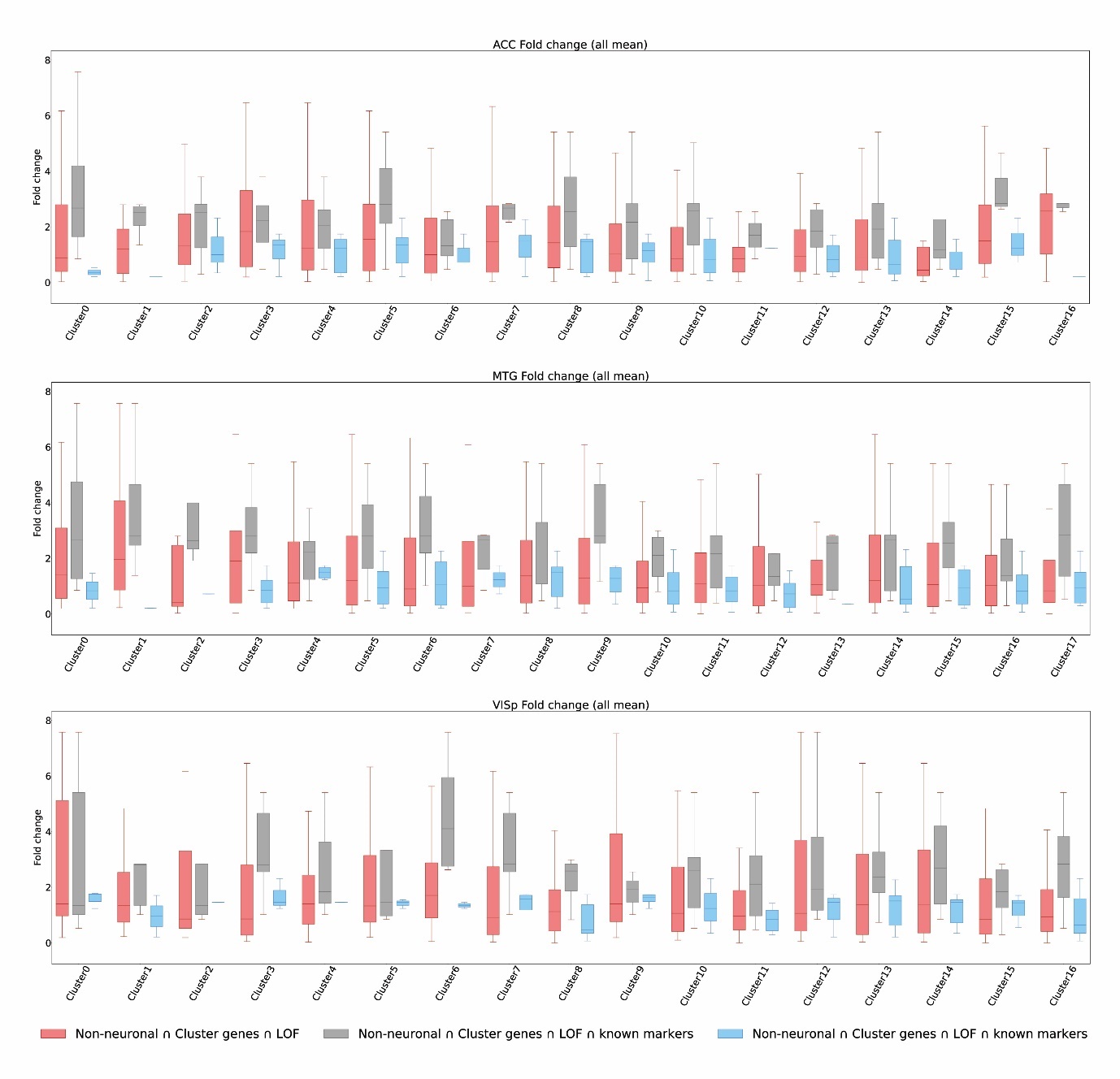


**Supplementary Figure. 16| Fold change of LOF genes in clusters across cell type in ACC, MTG, VISp.** Fold change of cluster genes wrt neurons - non neuronal ᴒ cluster genes ᴒ LOF (red), non-neuronal ᴒ cluster genes ᴒ LOF ᴒ known markers (grey), non-neuronal ᴒ cluster genes ᴒ housekeeping genes (blue).


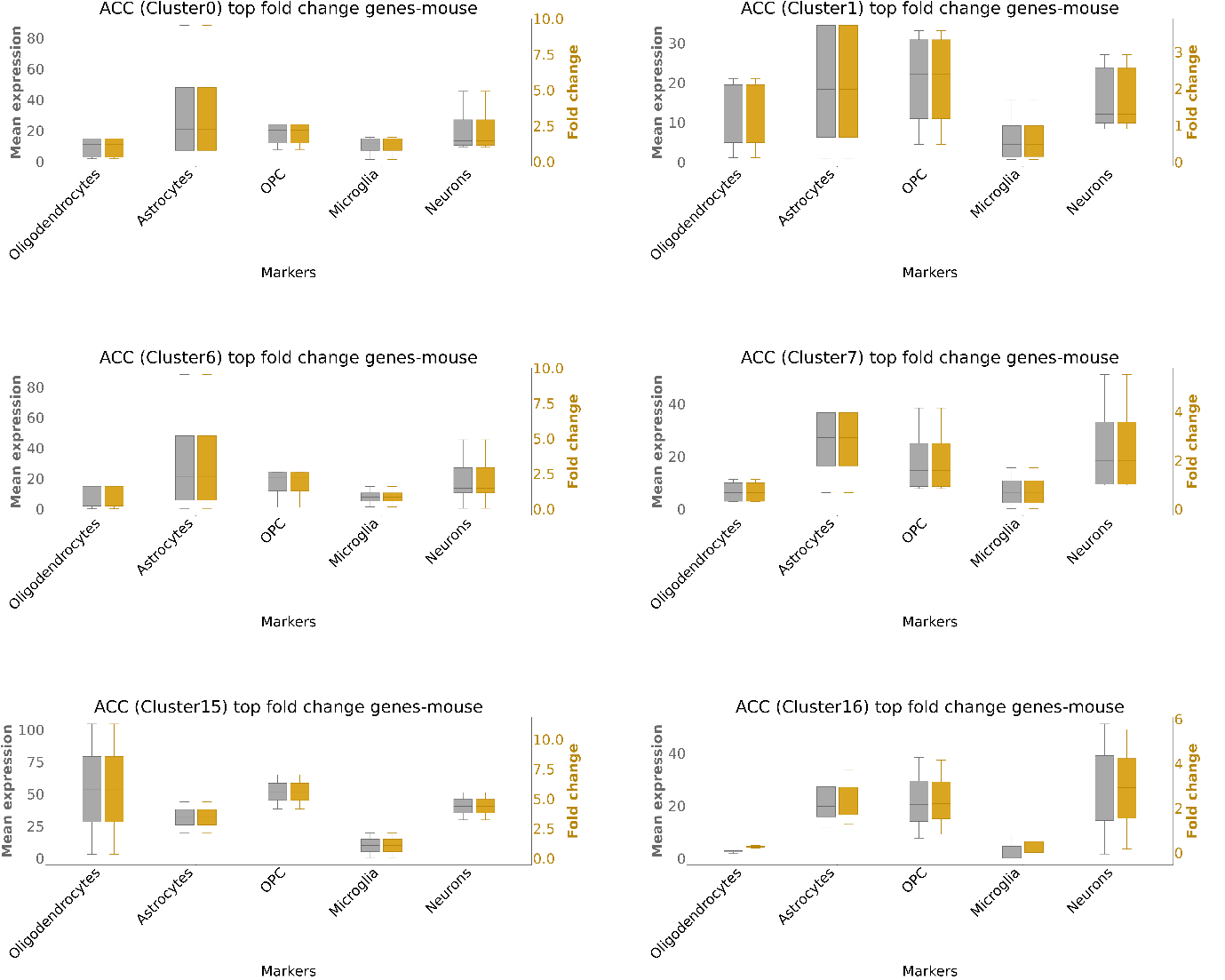

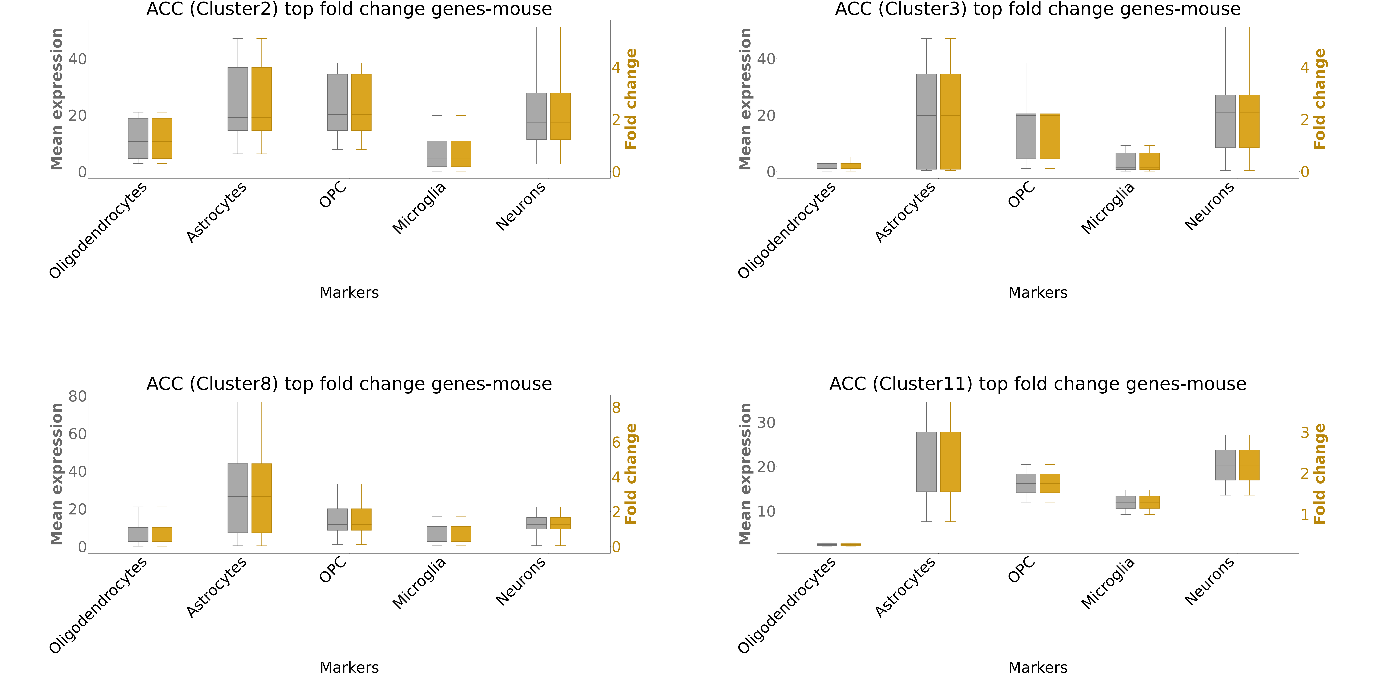

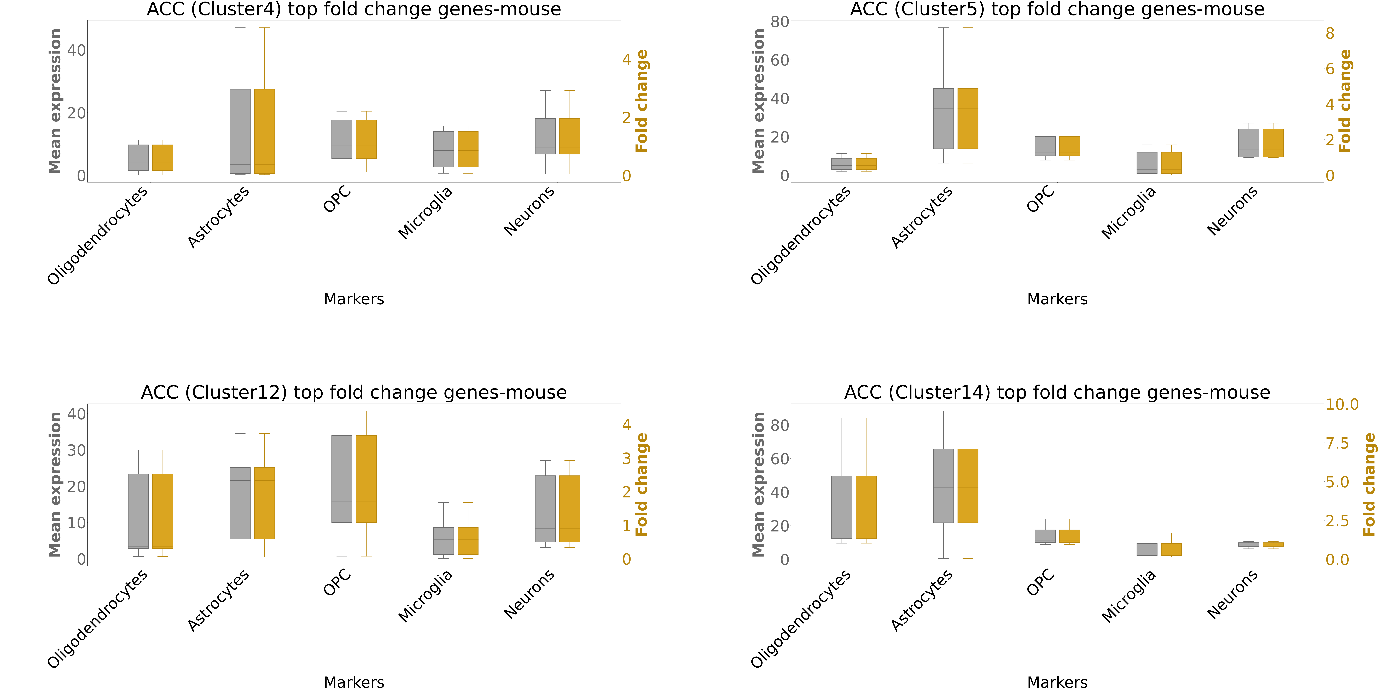

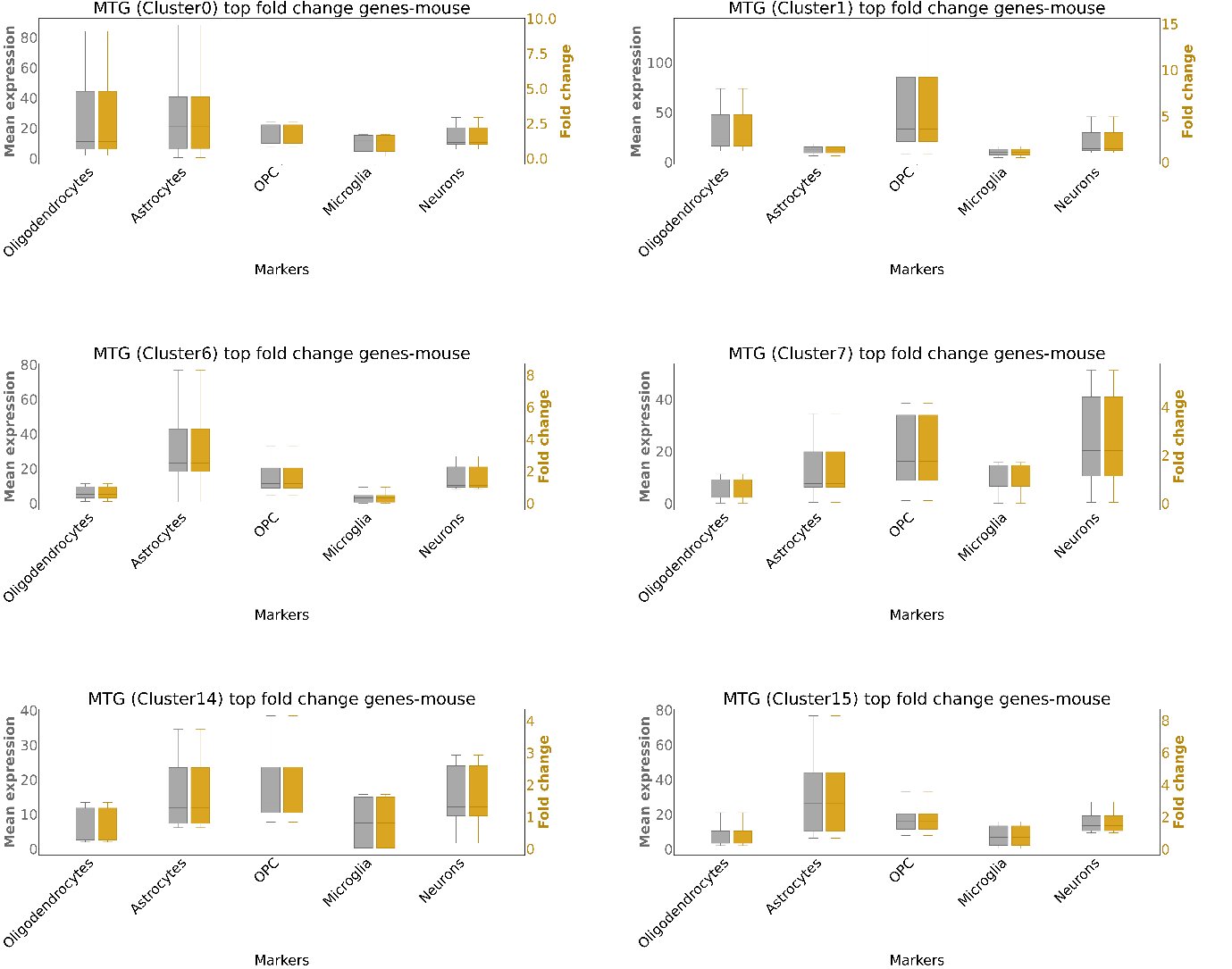

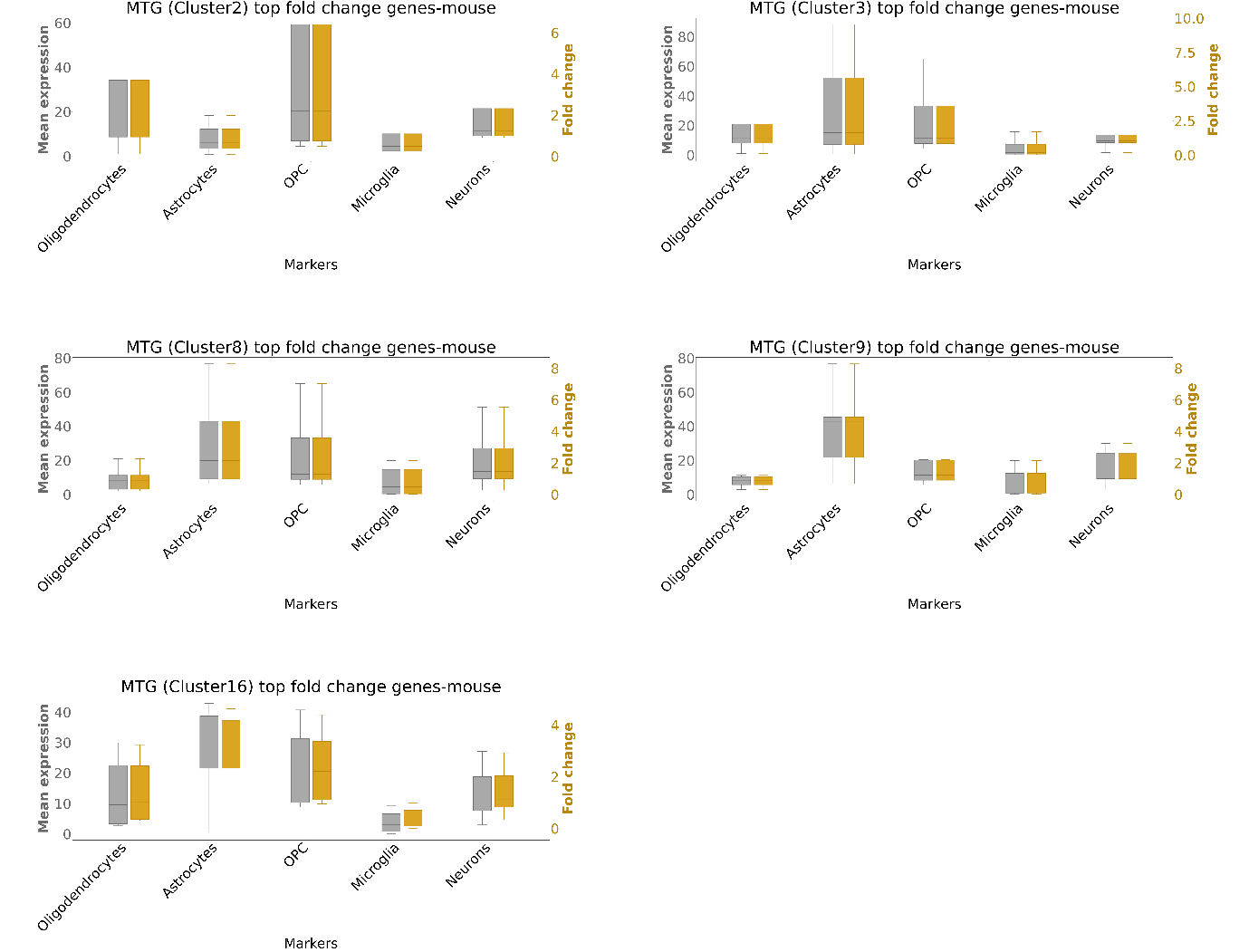

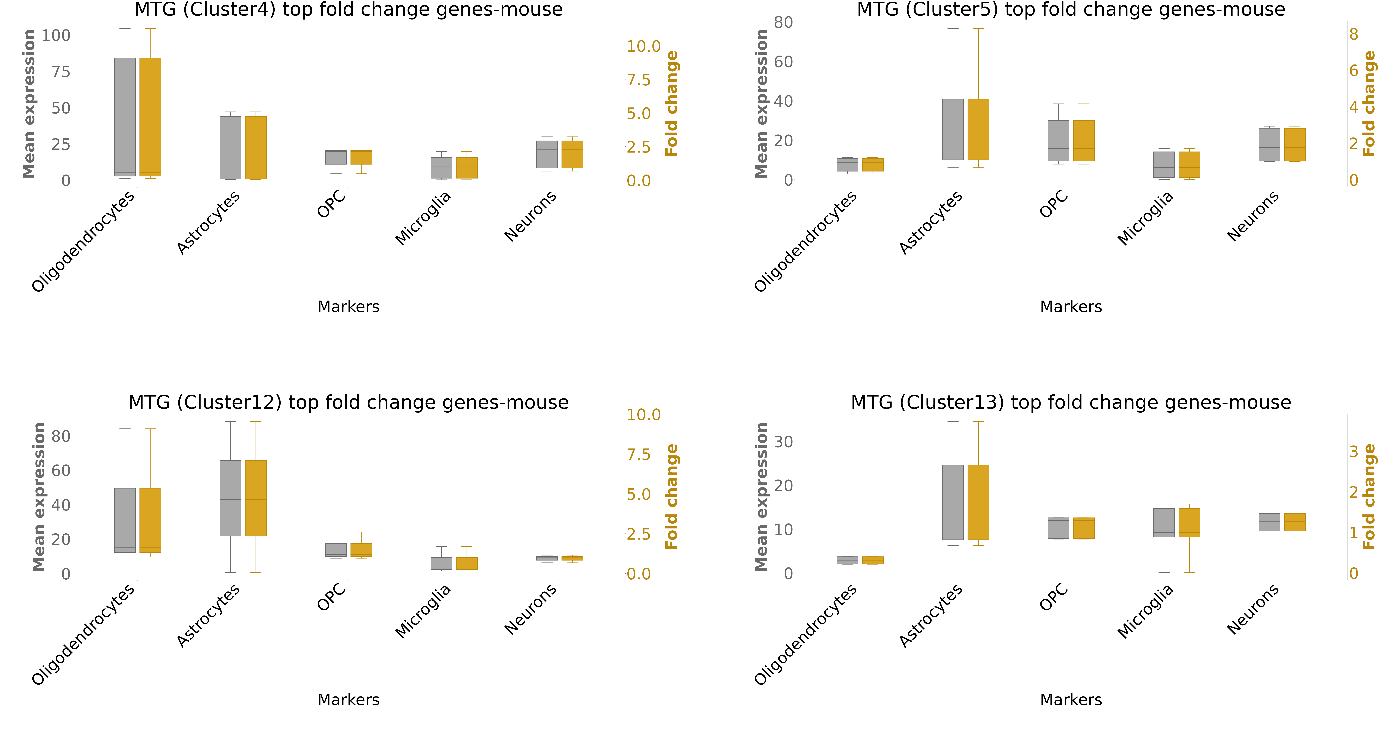

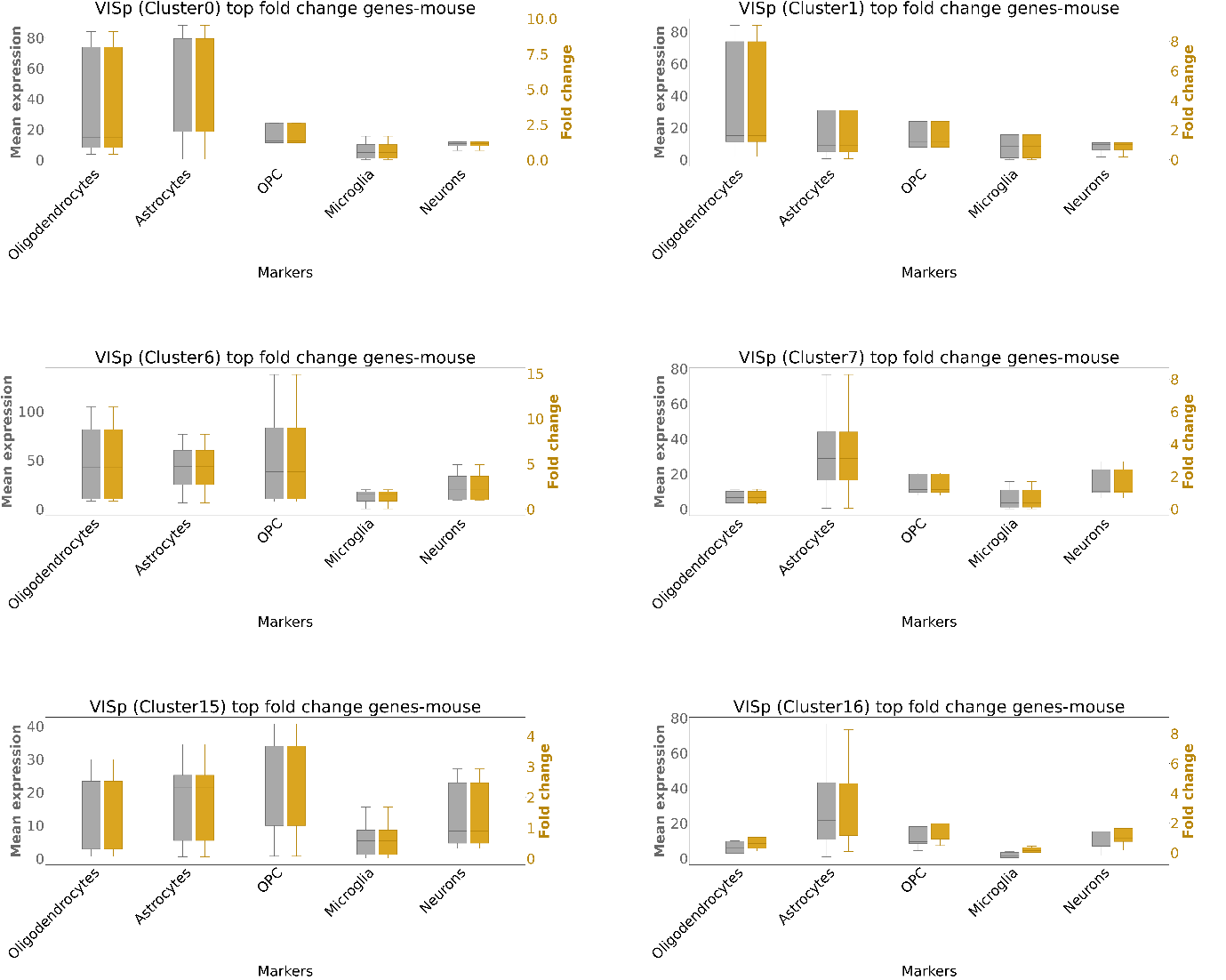

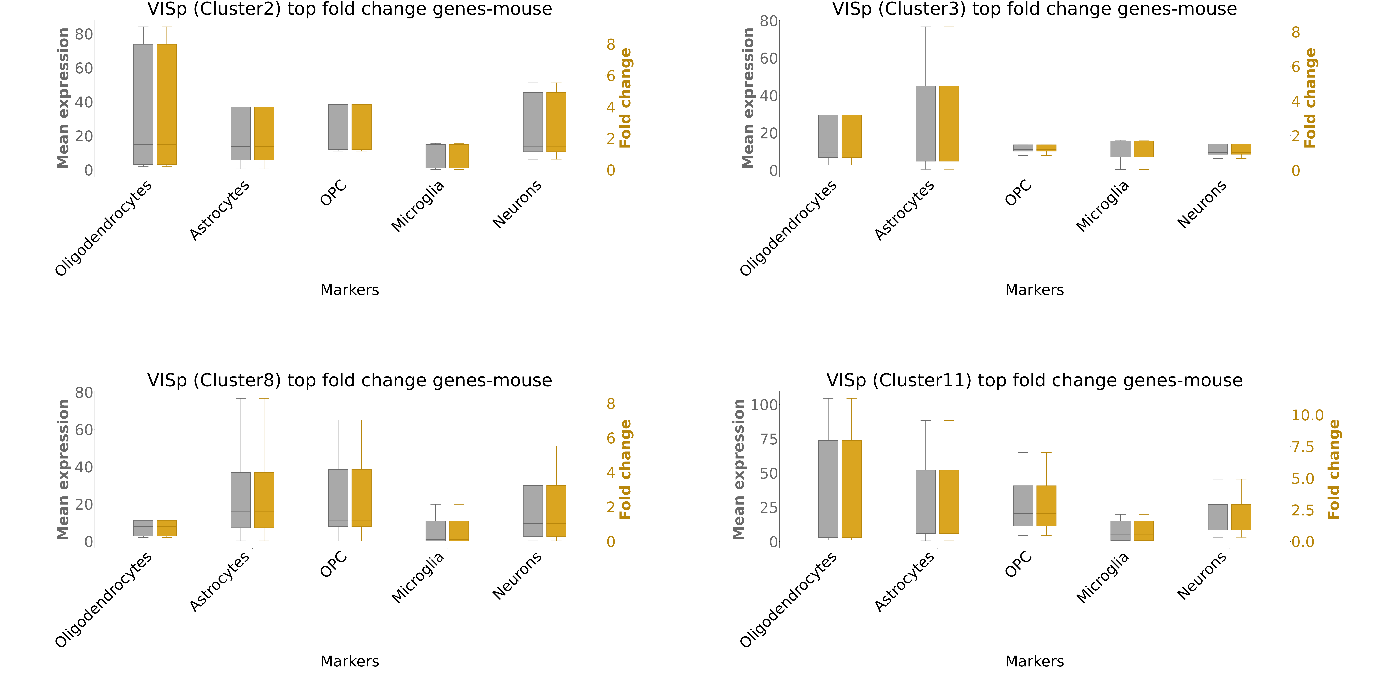

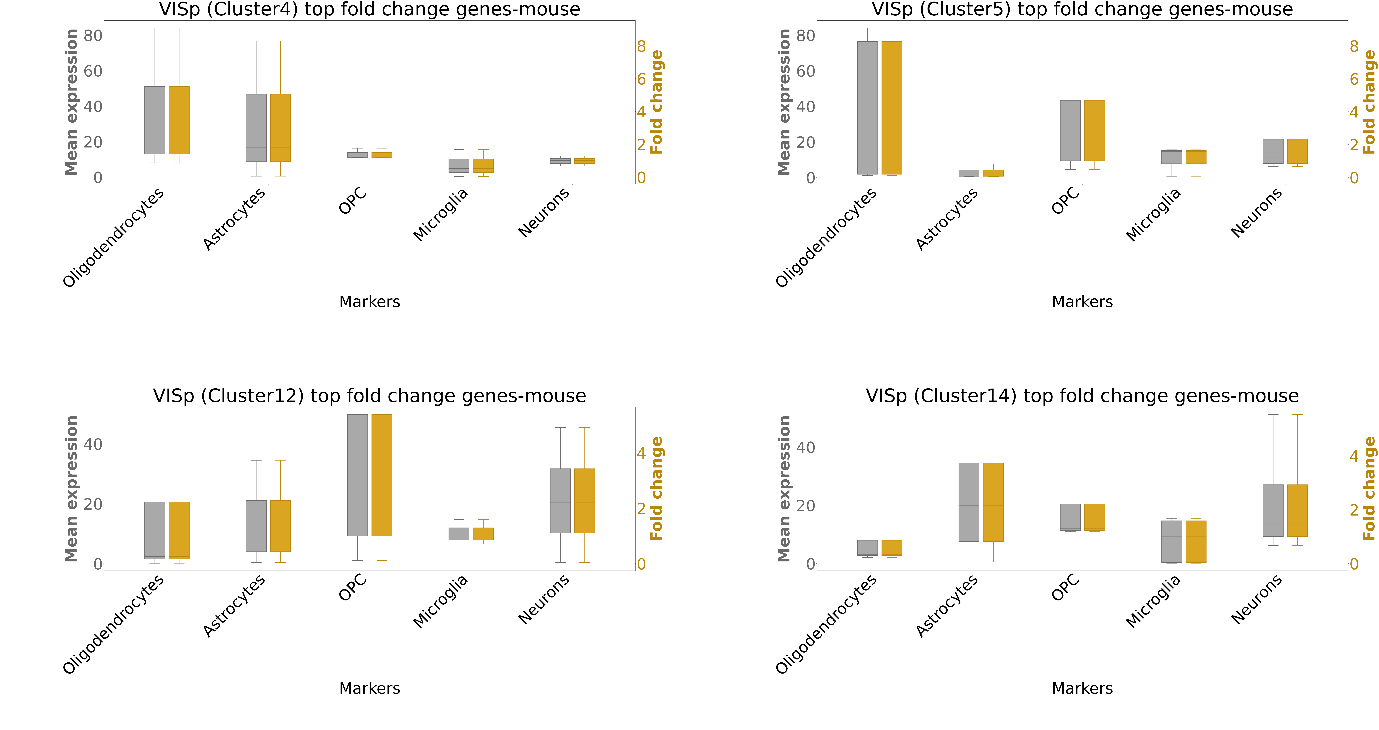


**Supplementary Figure. 17| Replication of associations between cluster genes and cell type in other datasets.** Mean expression of top fold change genes in clusters across ACC, MTG, VISP regions in GSE52564 (mouse). Fold change (yellow) calculated wrt neurons across oligodendrocytes, astrocytes, OPC, microglia and neurons.


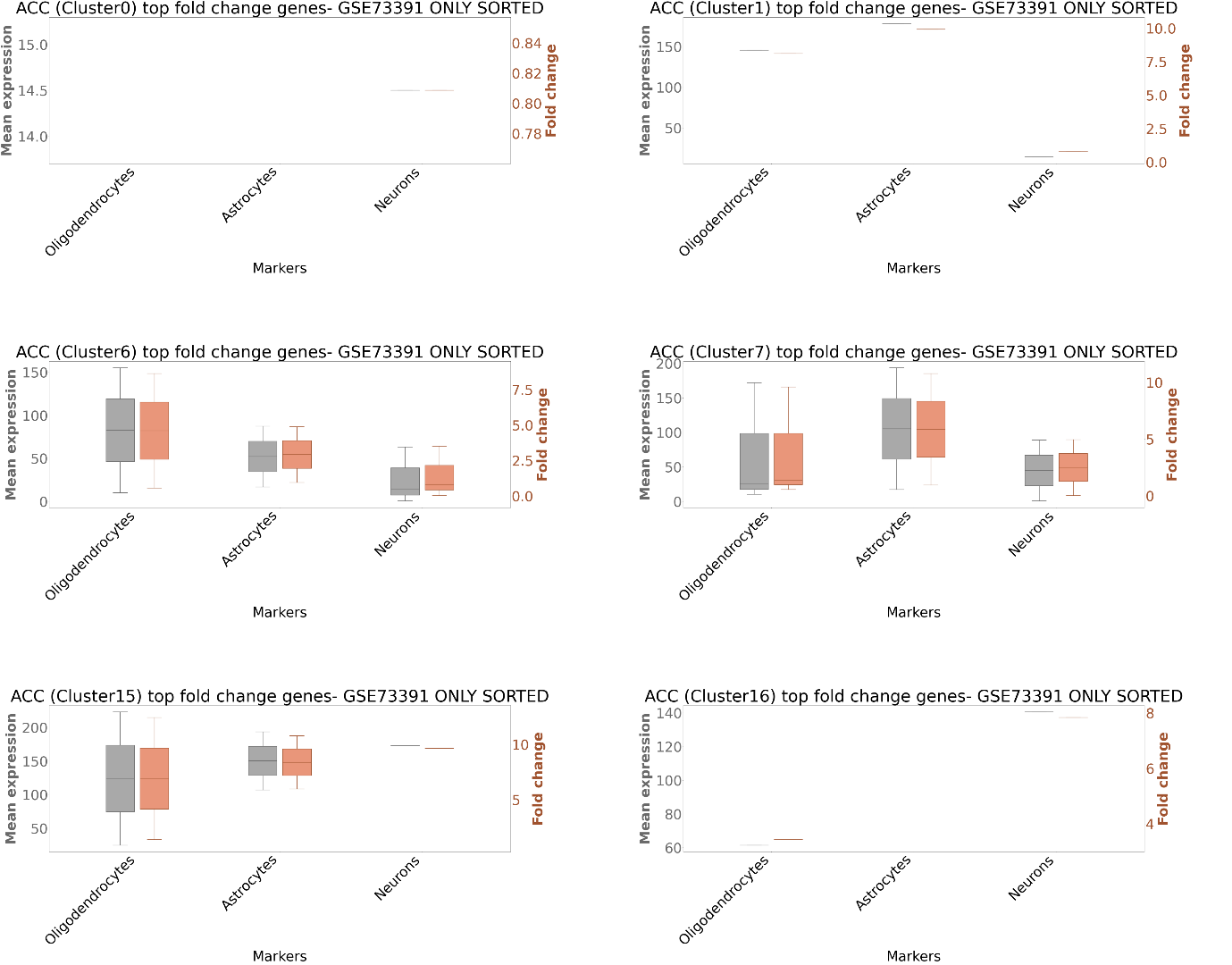

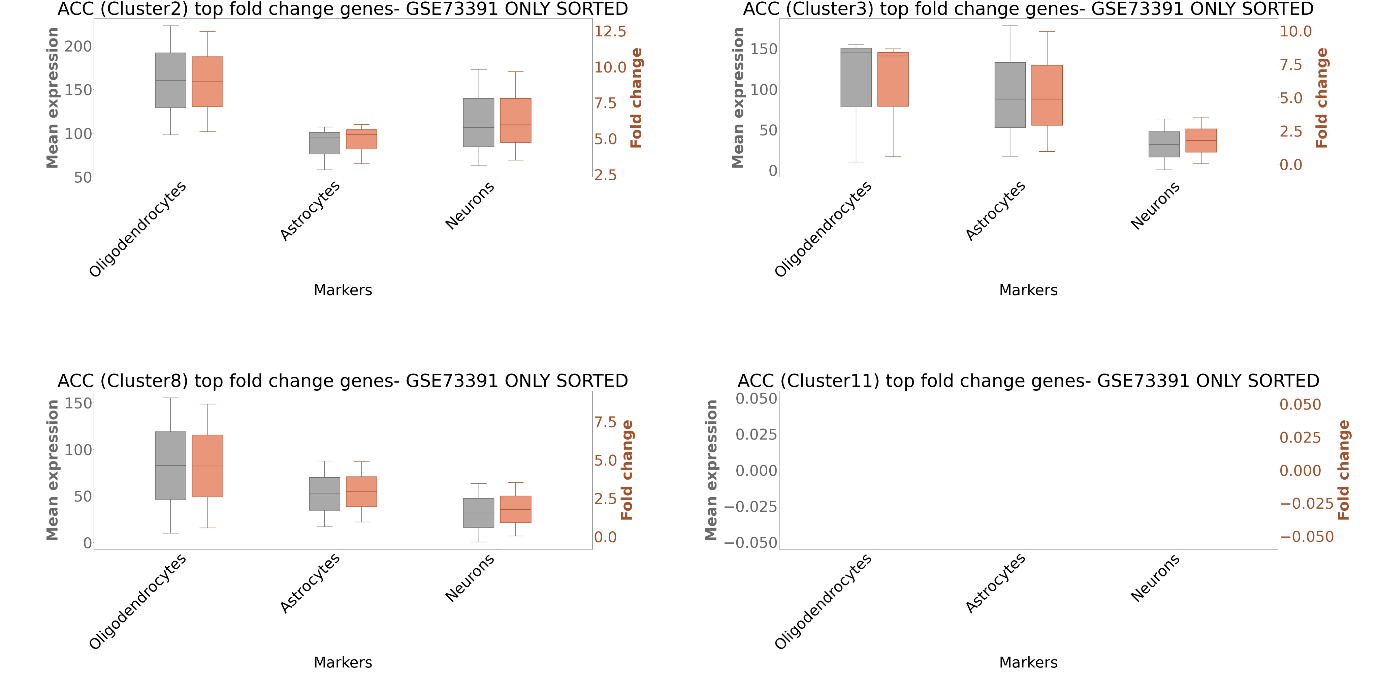

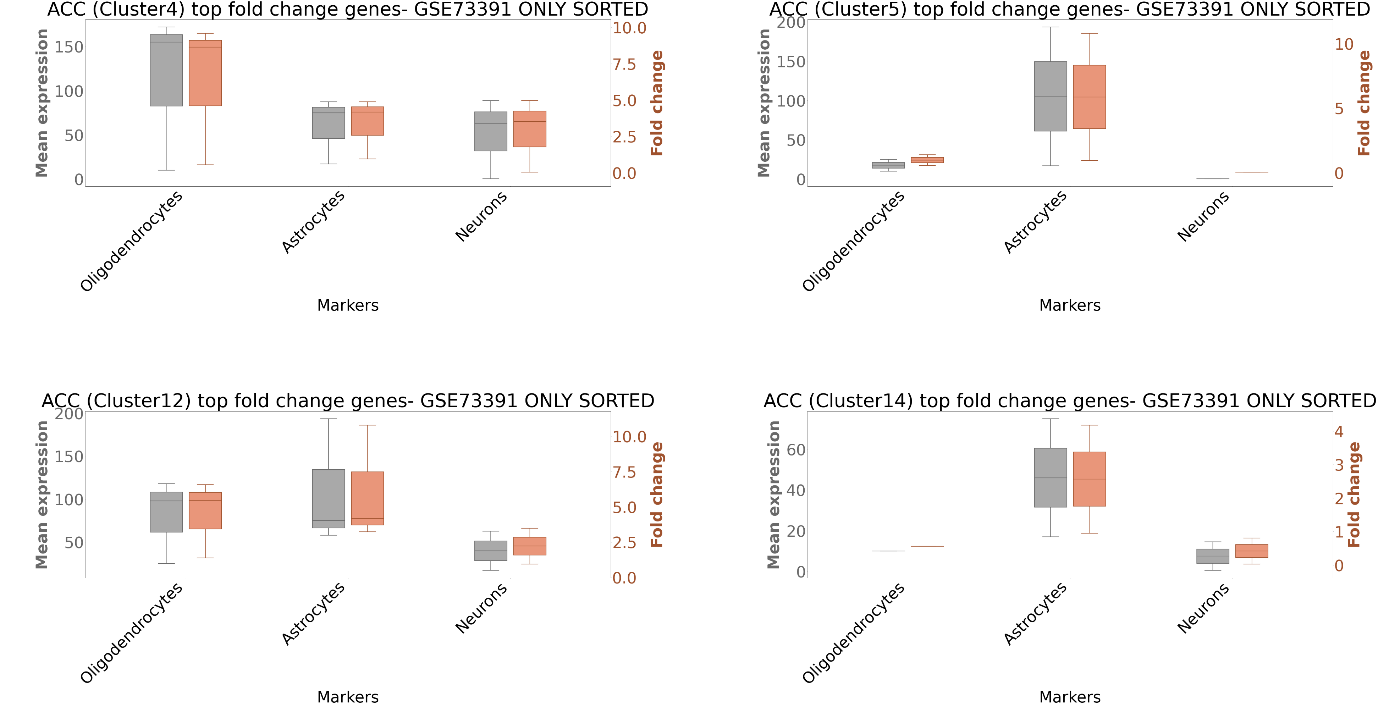

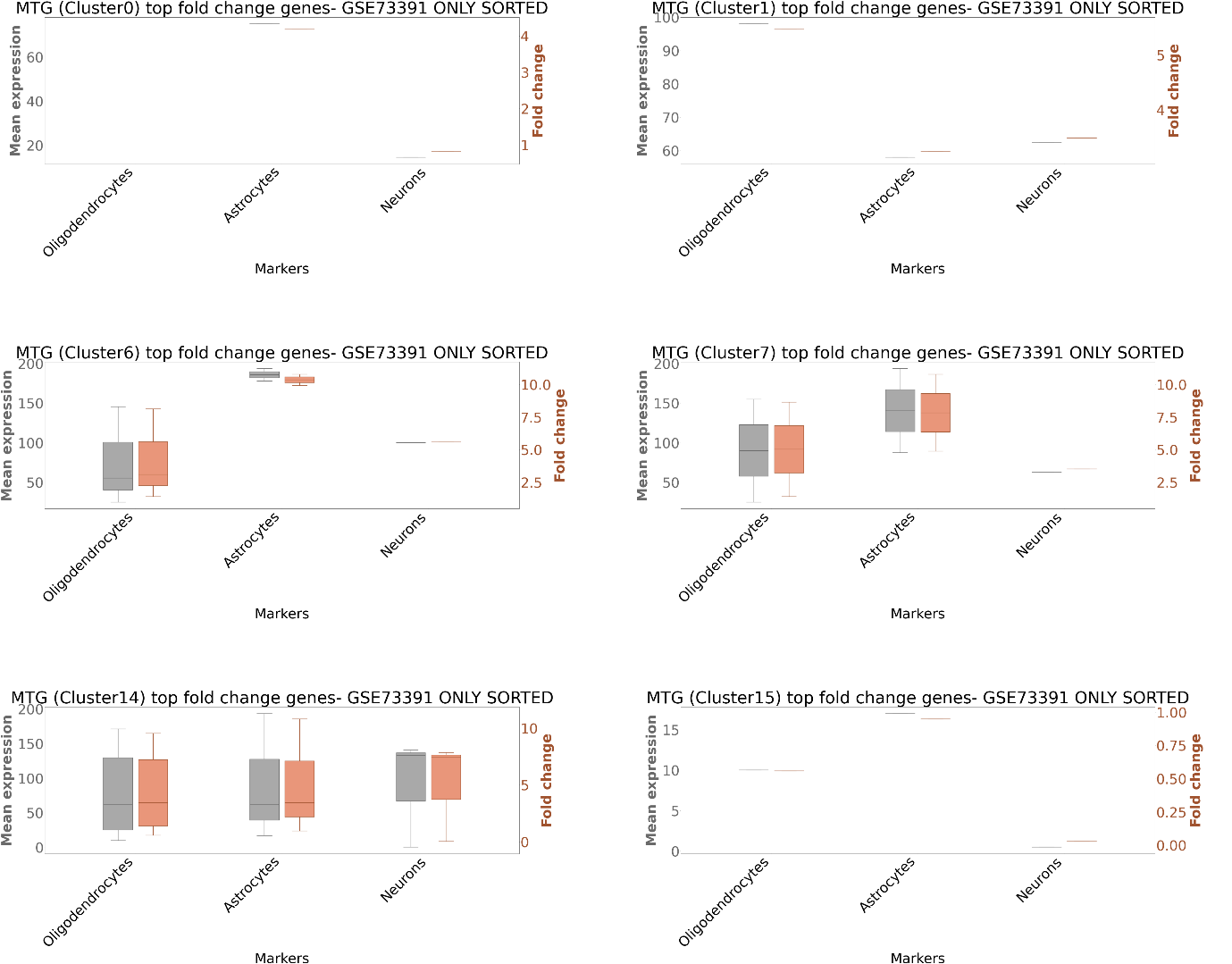

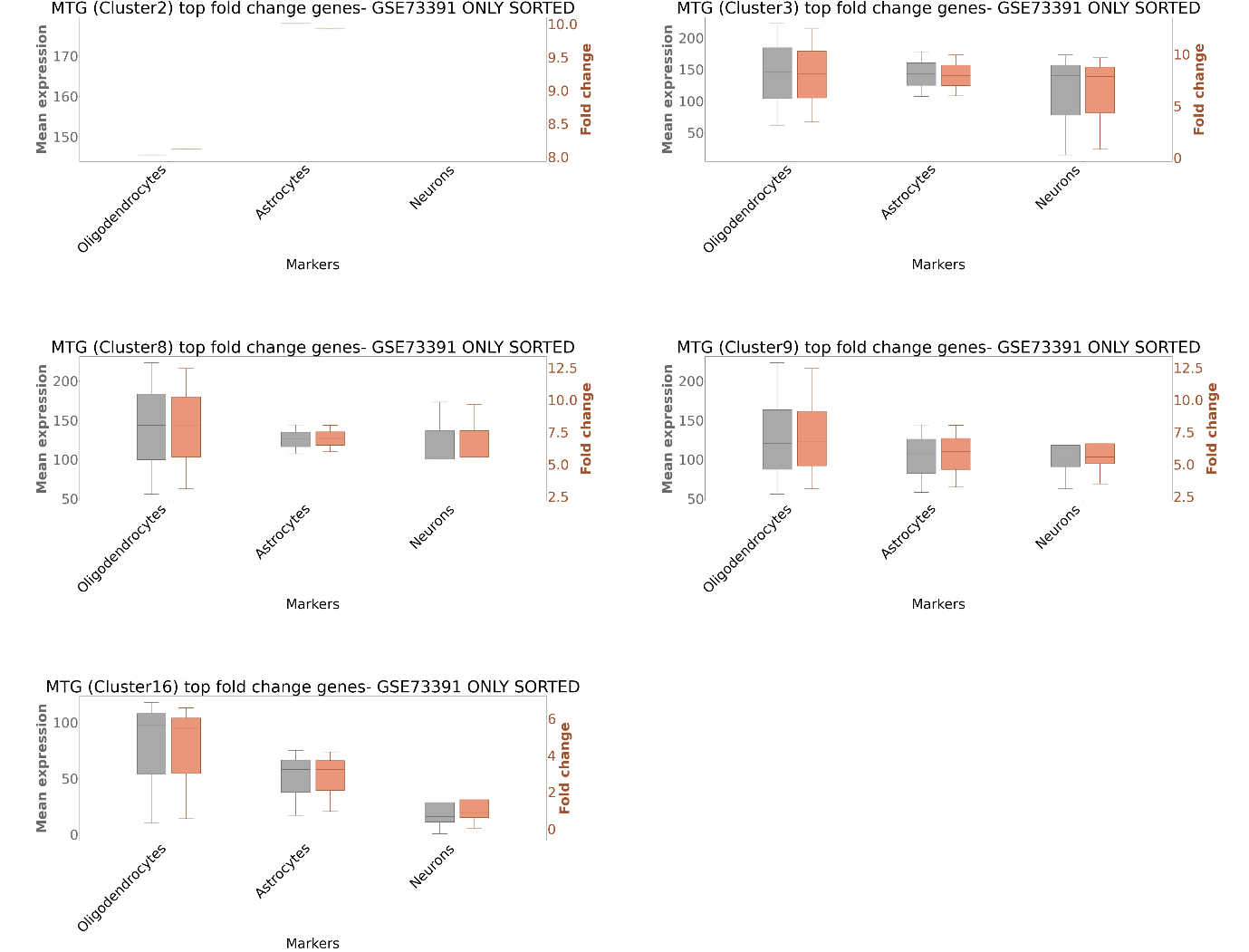

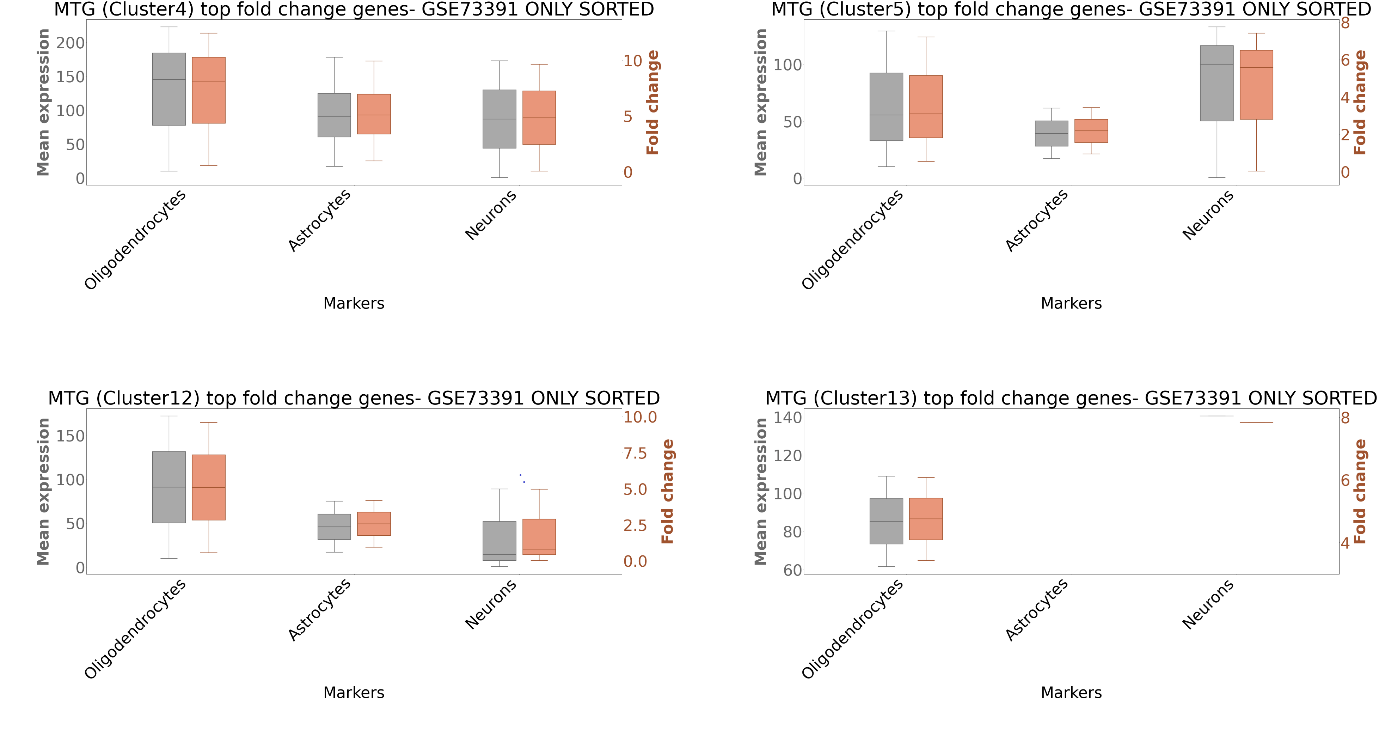

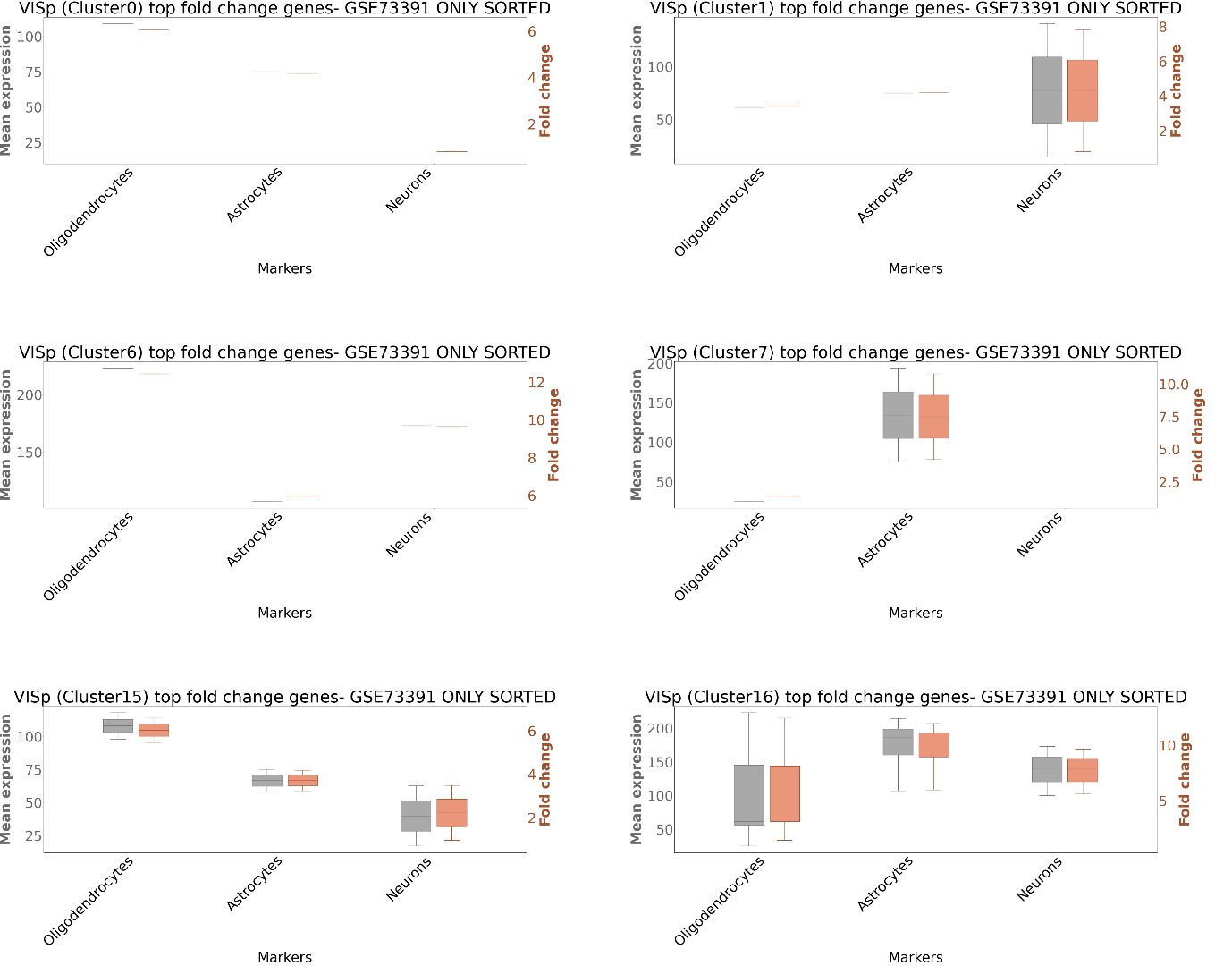

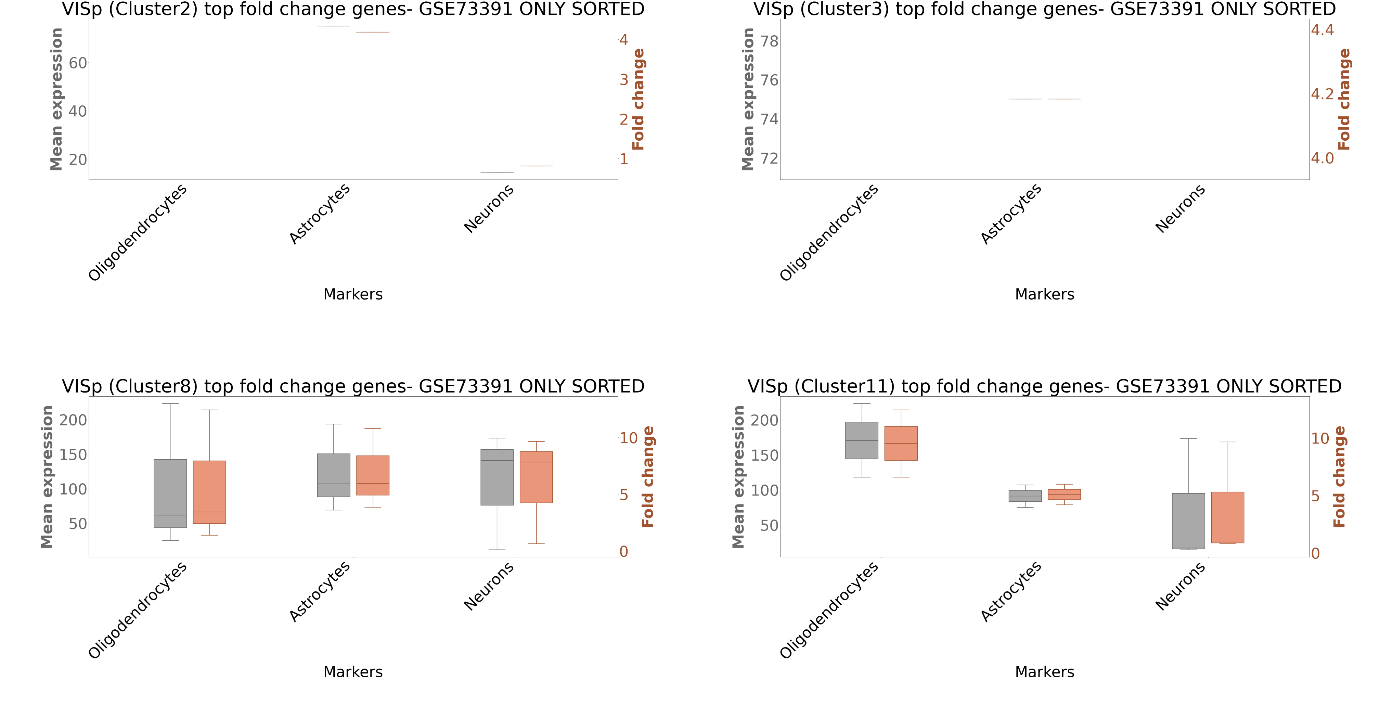

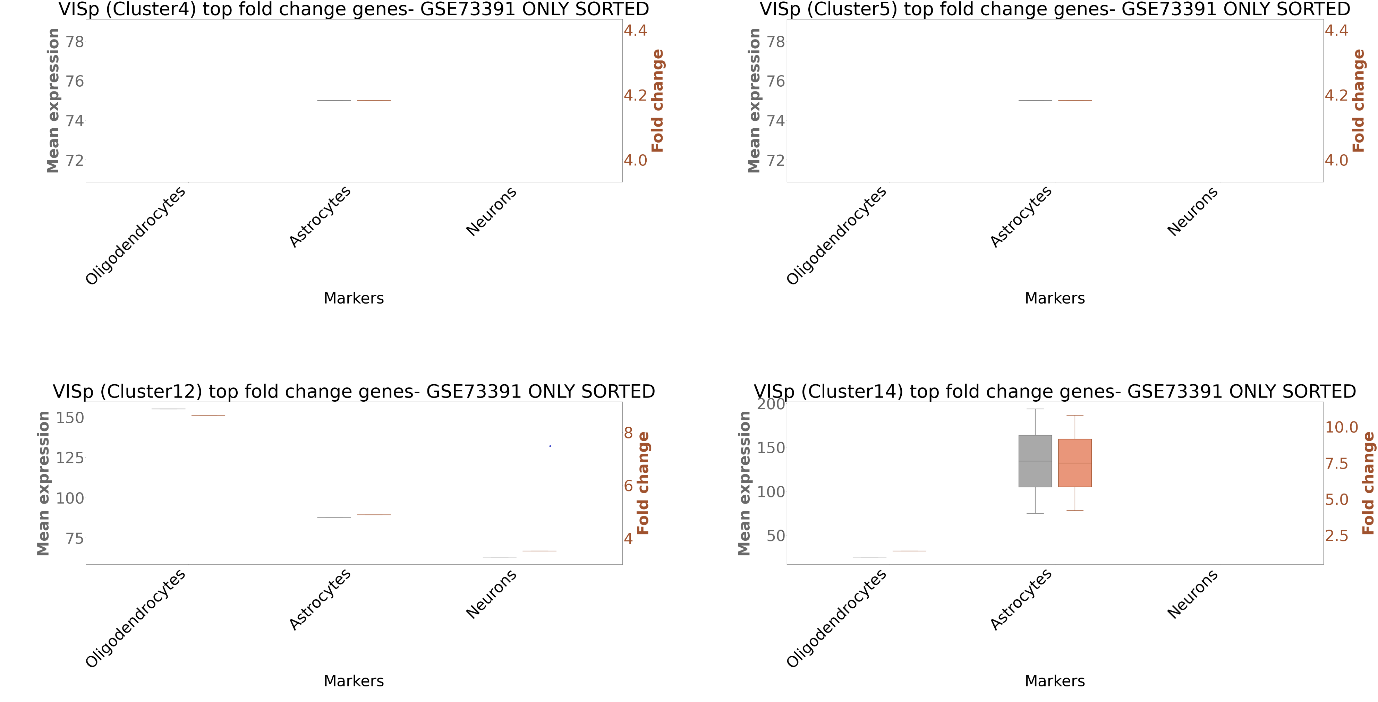


**Supplementary Figure. 18| Mean expression of top fold change genes in clusters across ACC, MTG, VISP regions in GSE73391 (mouse).** Fold change (red) calculated wrt neurons across oligodendrocytes, astrocytes, OPC, microglia and neurons.


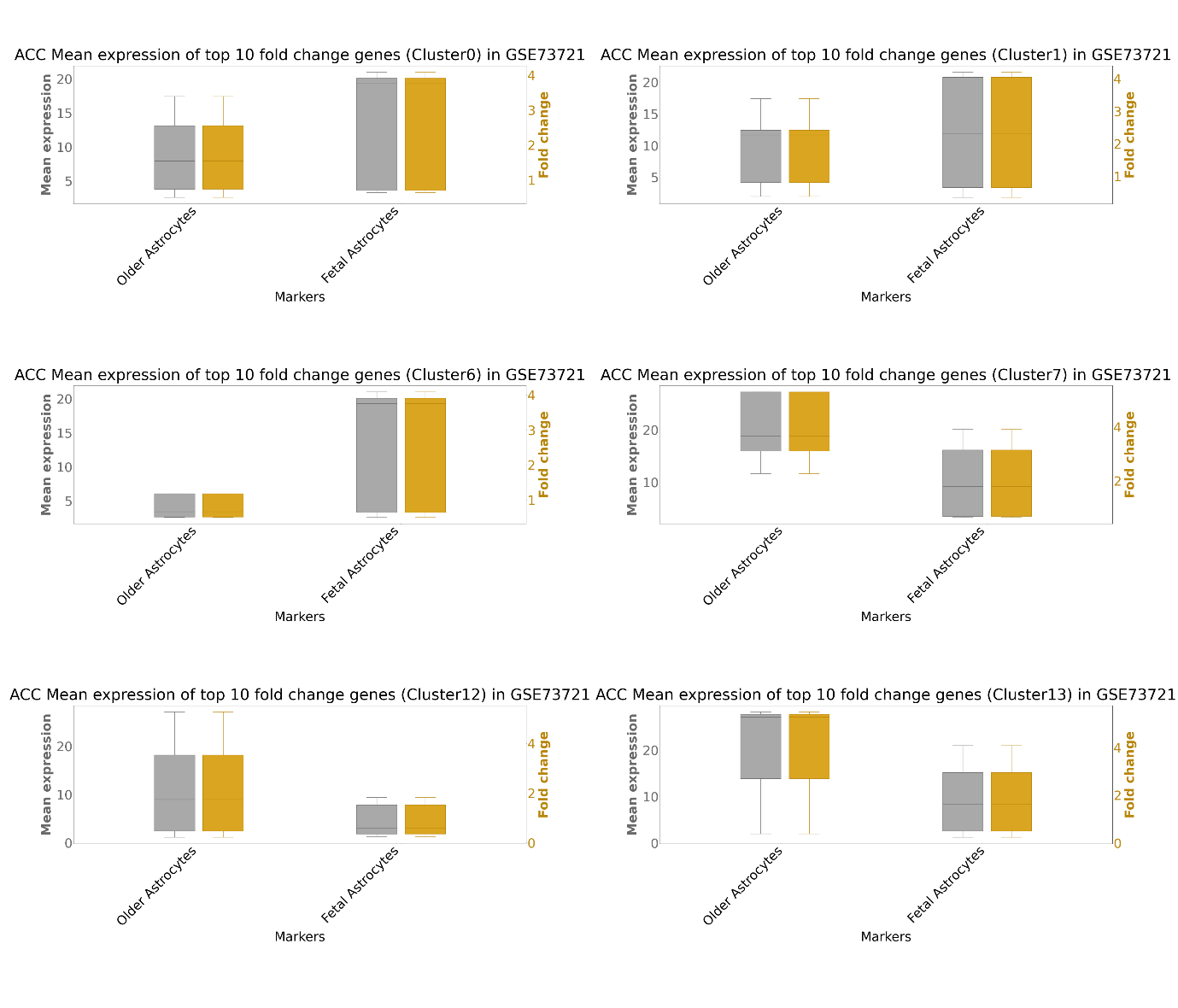


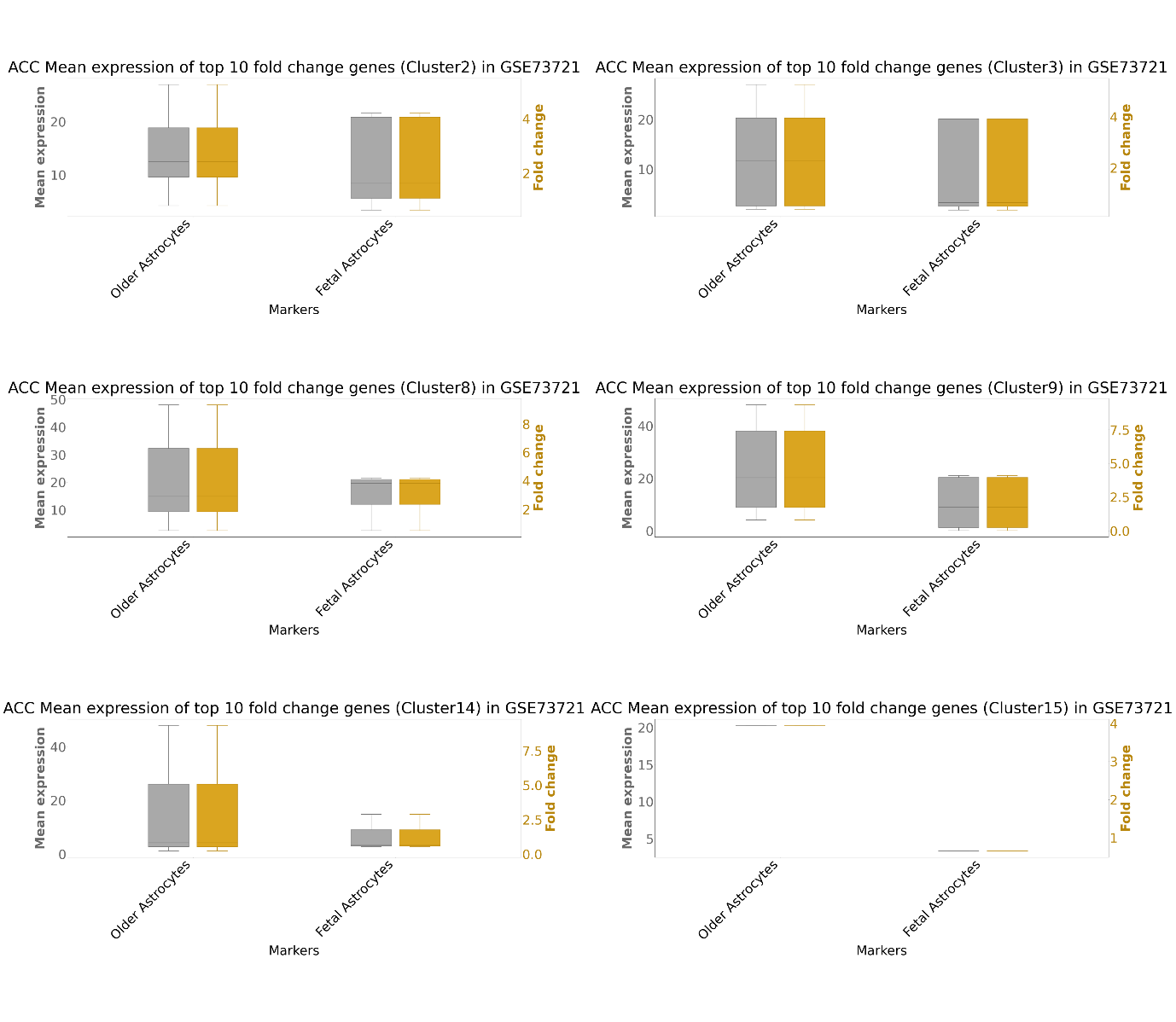

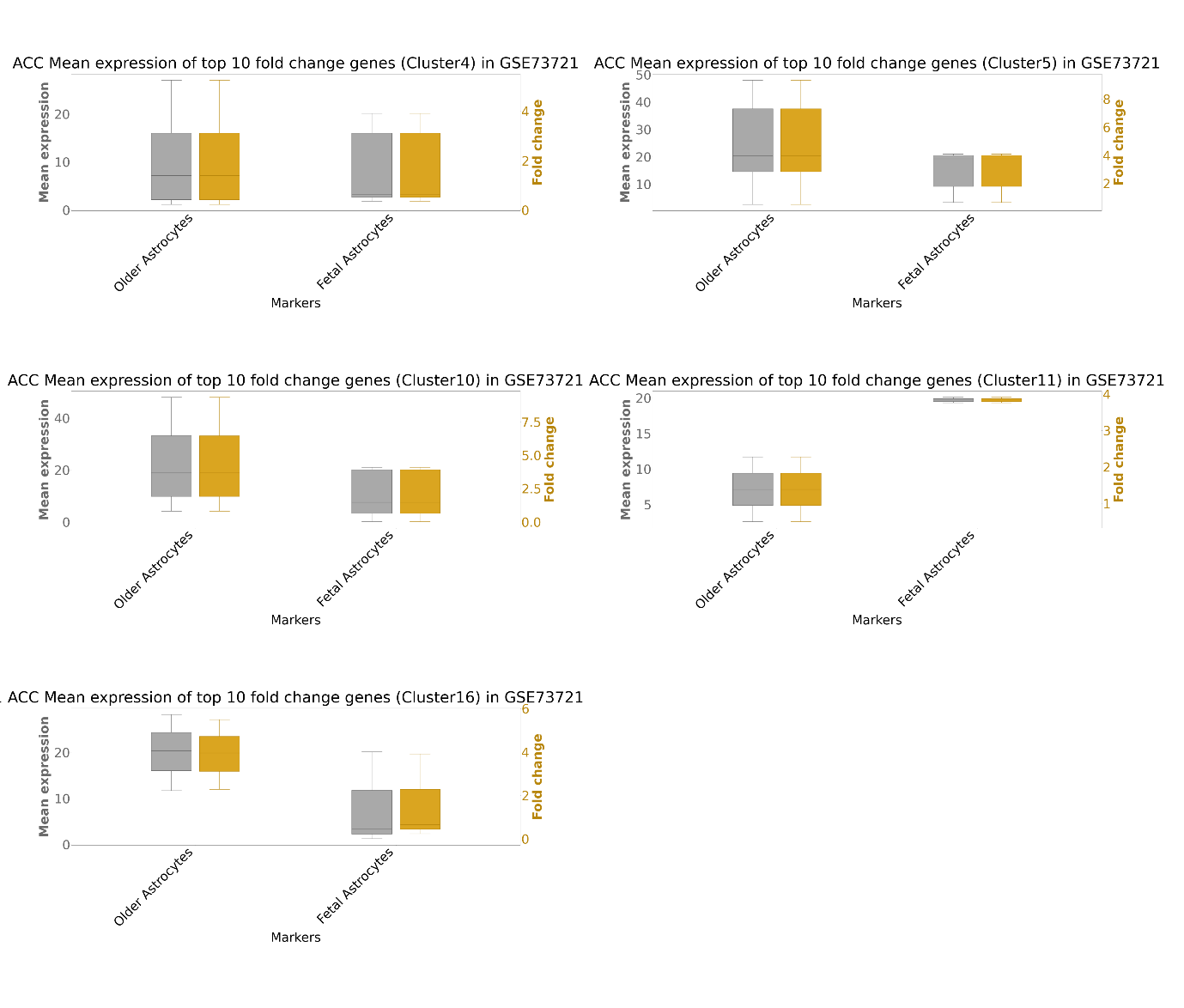

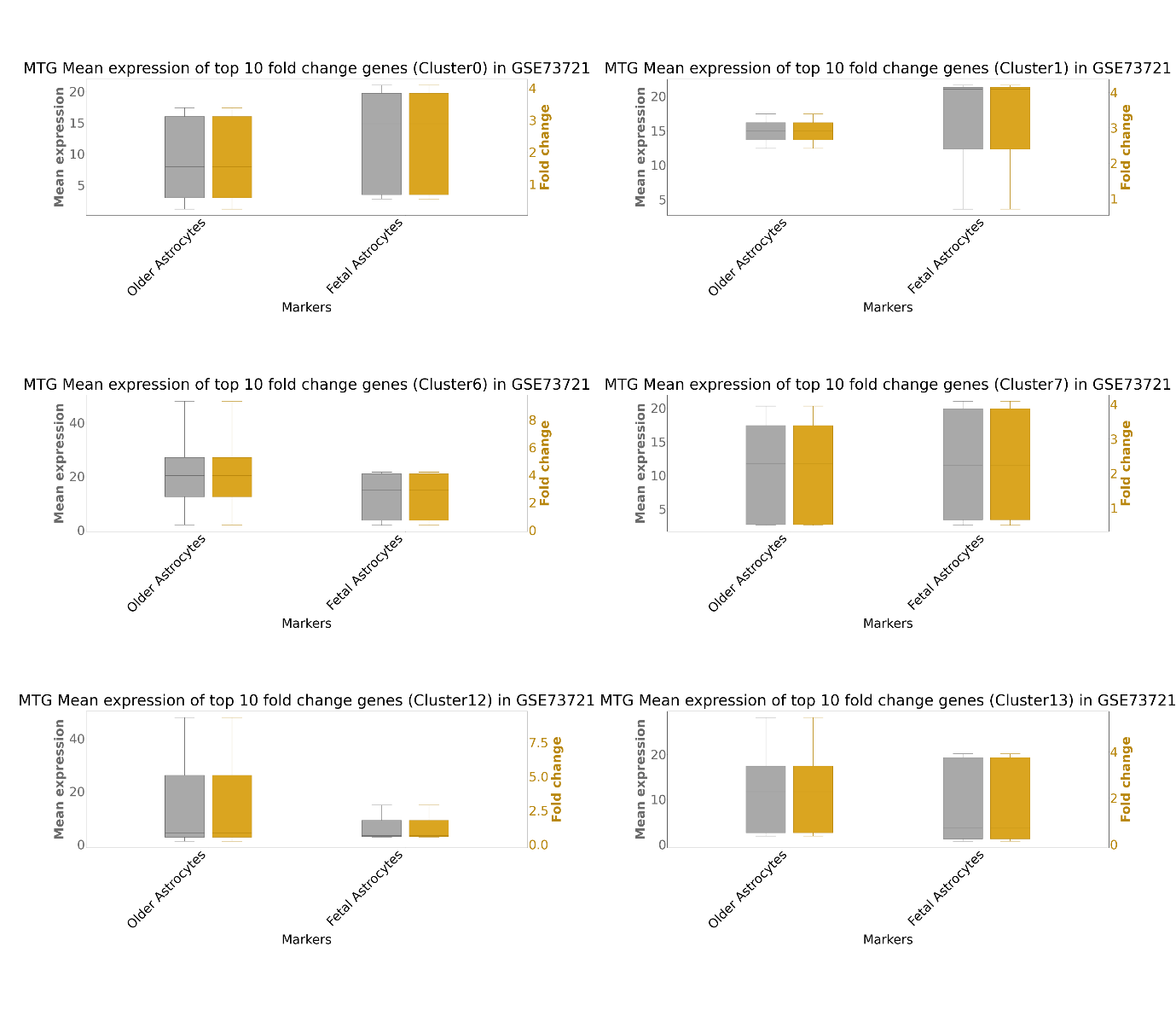

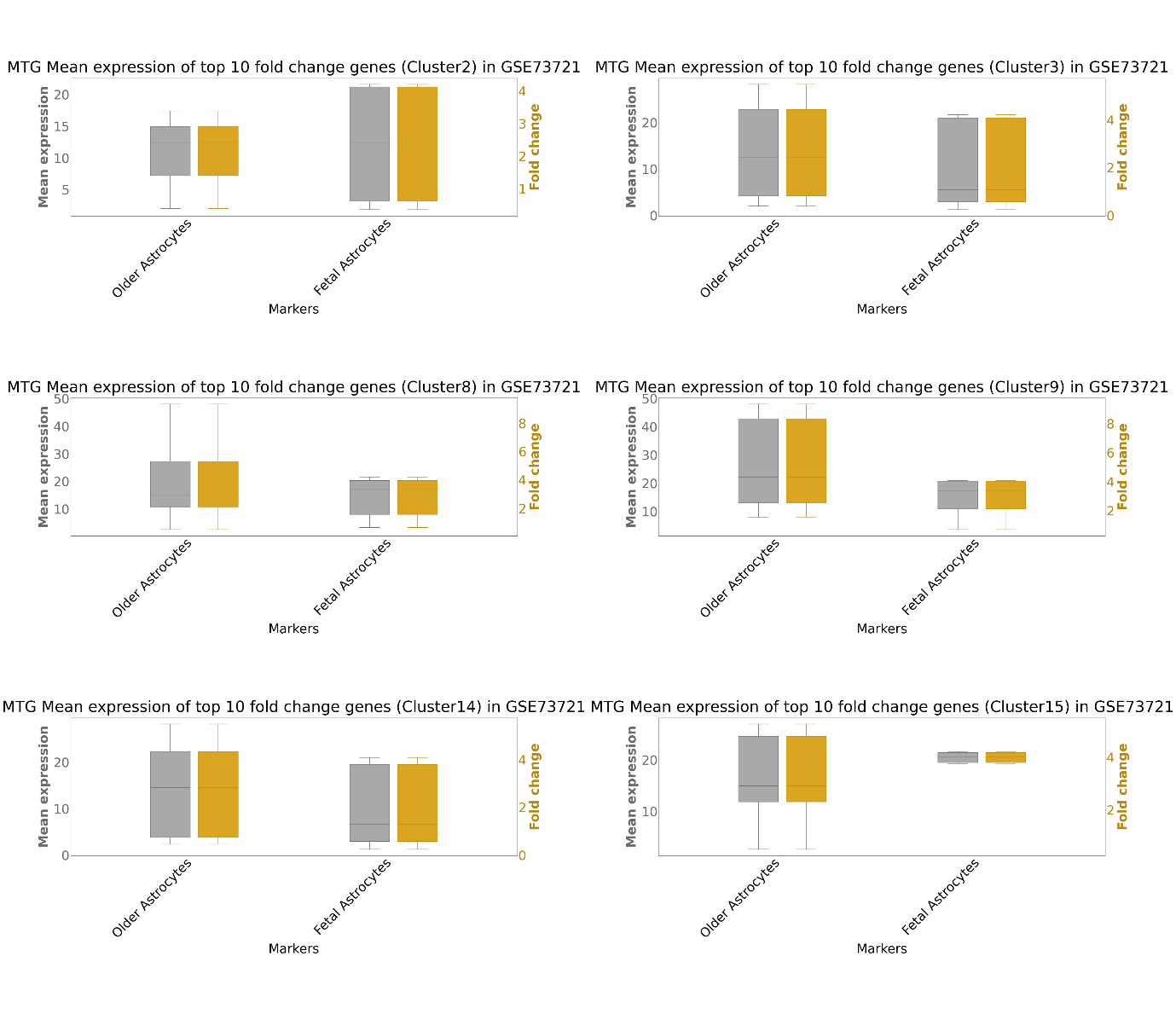

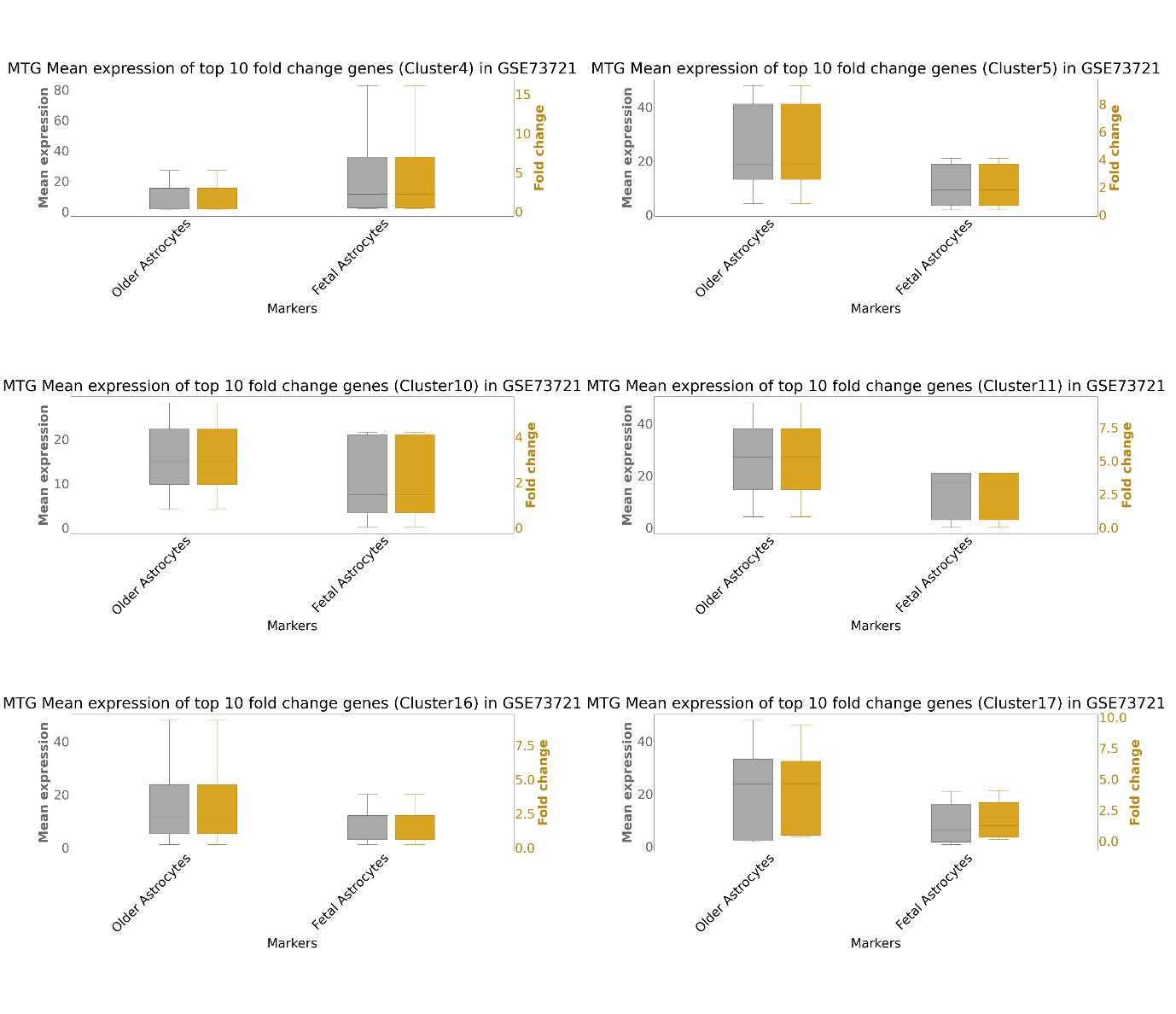

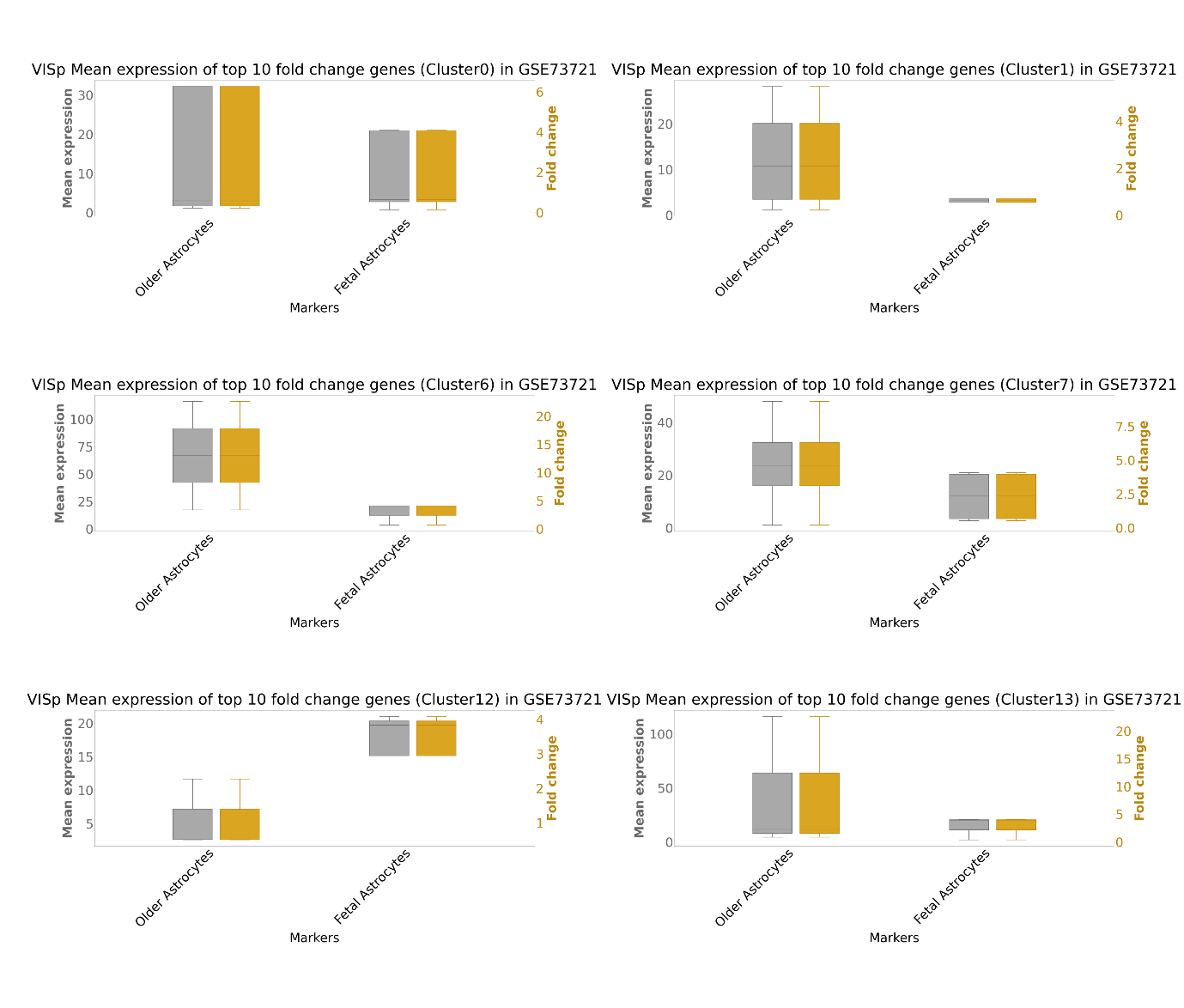

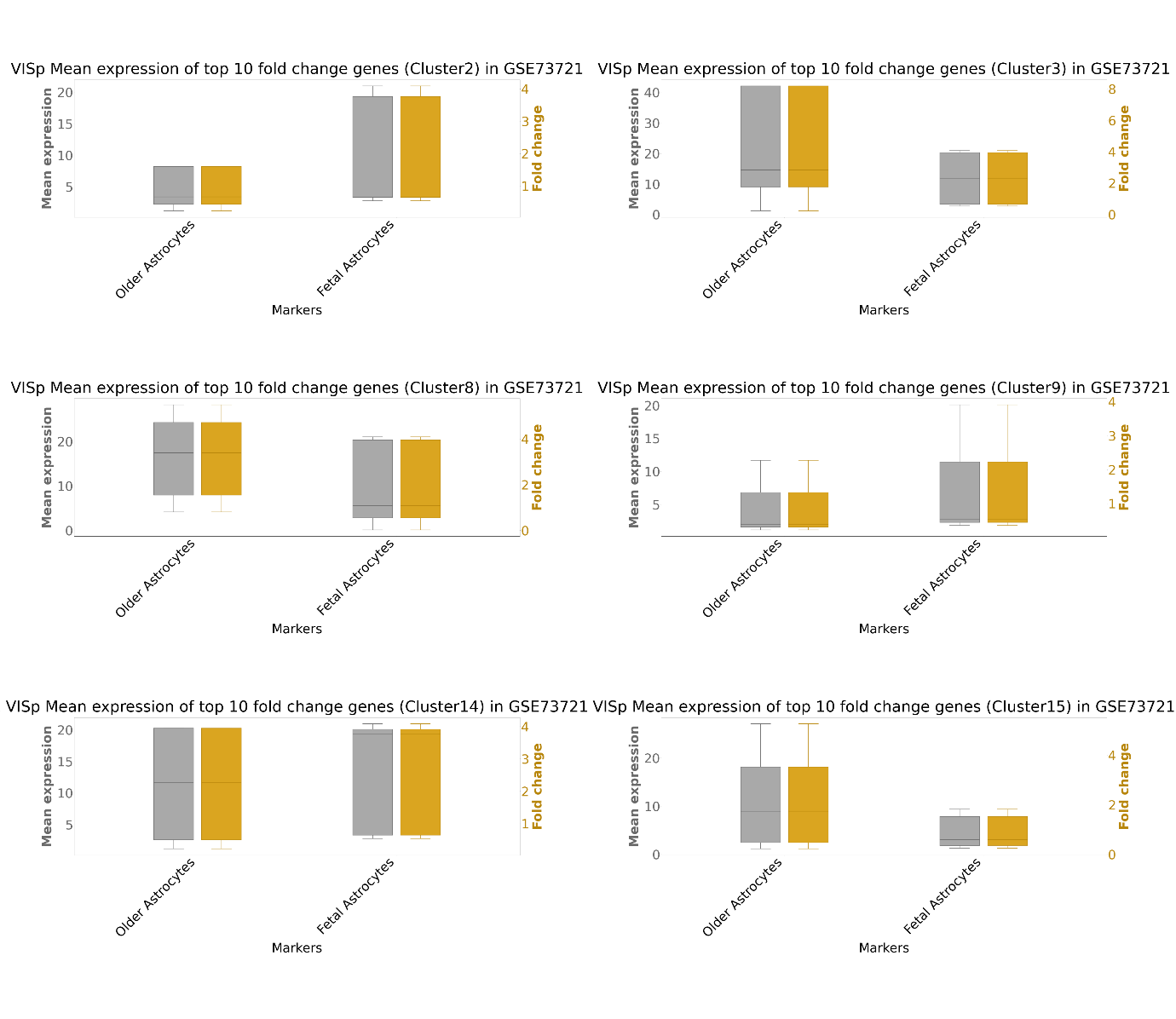

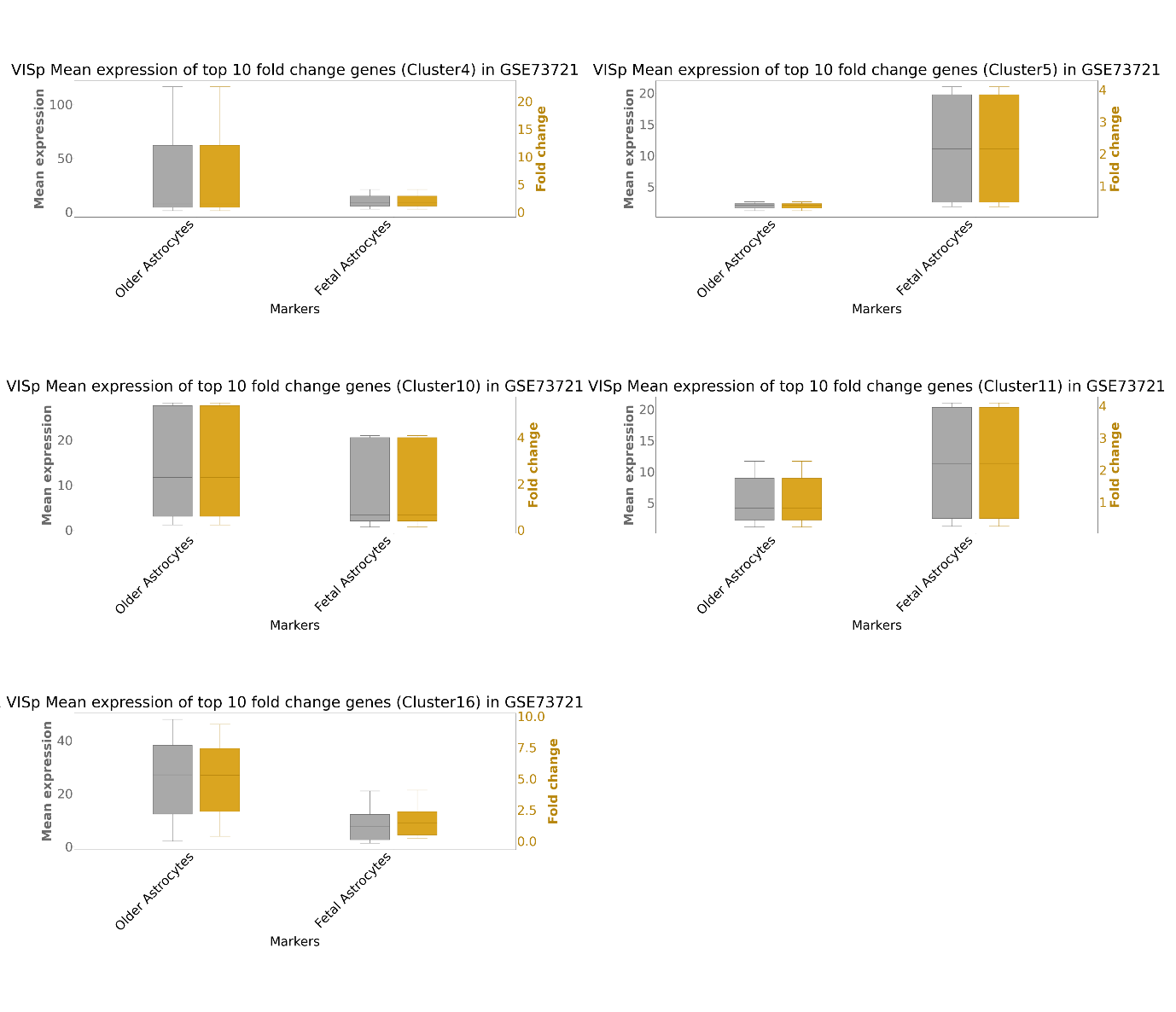


**Supplementary Figure. 19| Mean expression of top fold change genes in clusters across ACC, MTG, VISP regions.** Mean expression (grey) and fold change (yellow) in adult and fetal astrocytes (GSE73721).


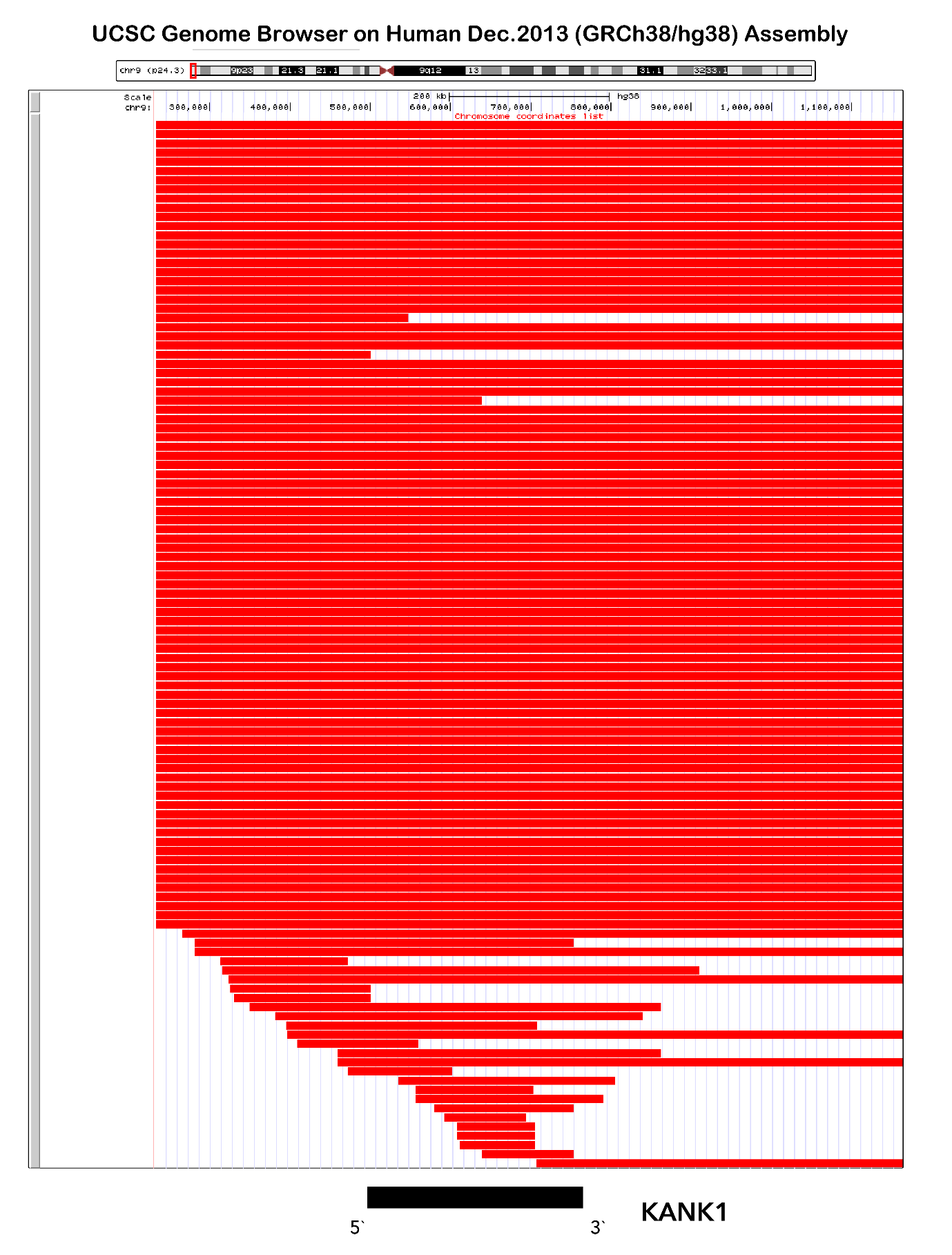


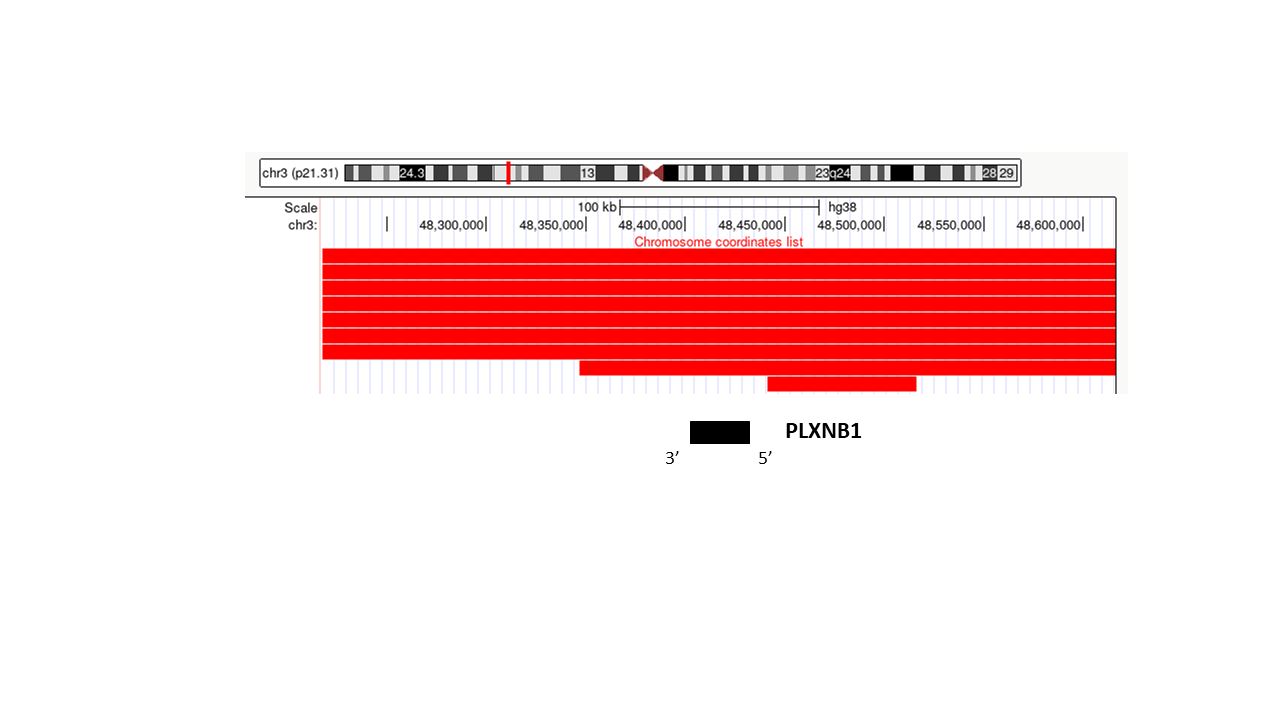


**Supplementary Figure. 20| Genes impacted with clinically relevant mutations with restricted non-neuronal brain cell expression.** CNVs affecting A) *KANK1* and B) *PLXNB1* gene collected from literature and ClinVar database.

**ACC**


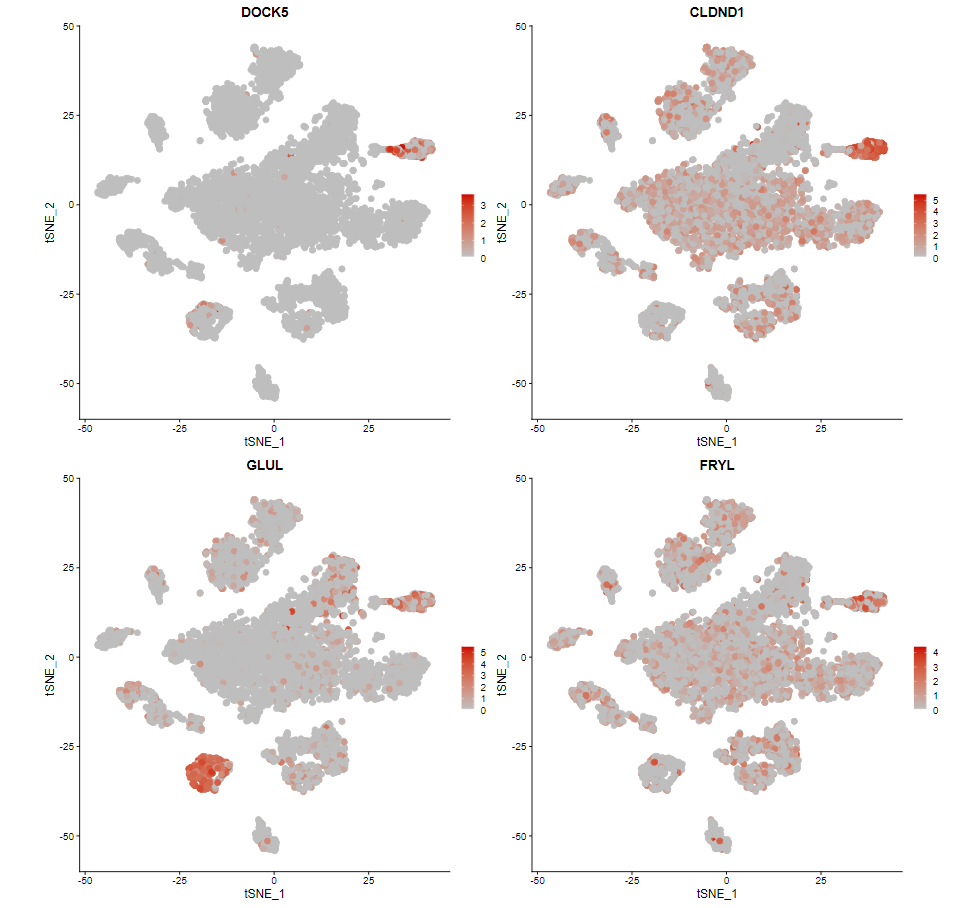


**MTG**


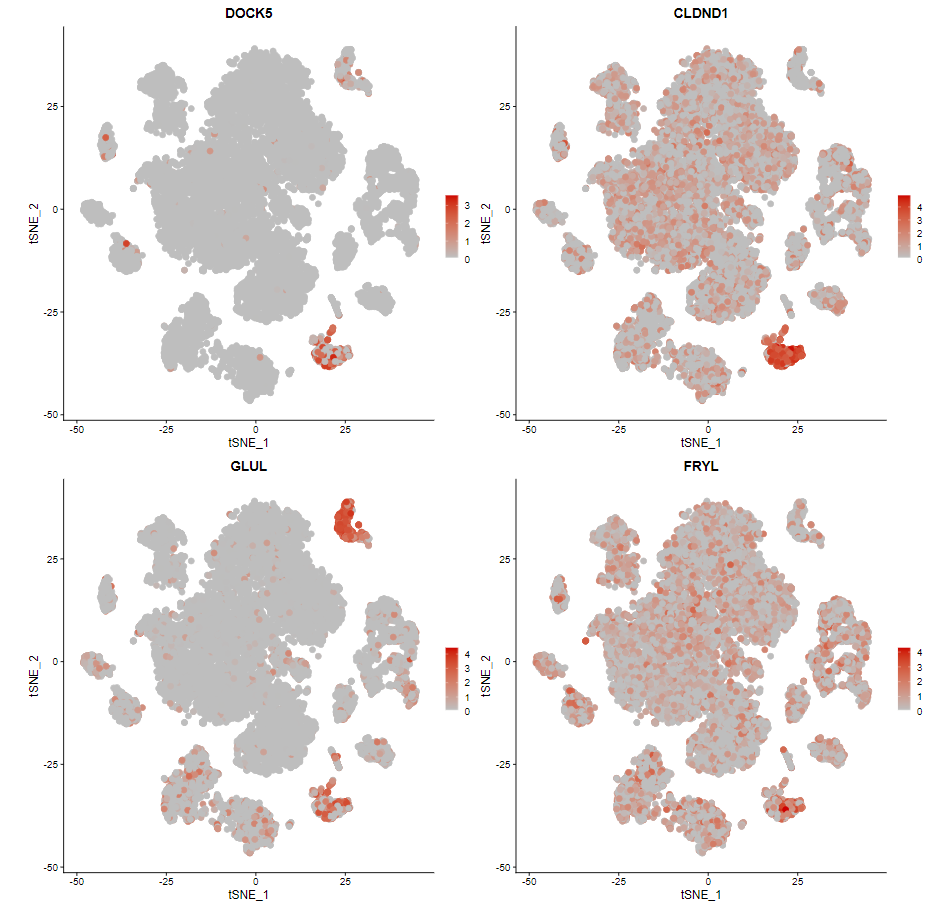


**VISP**


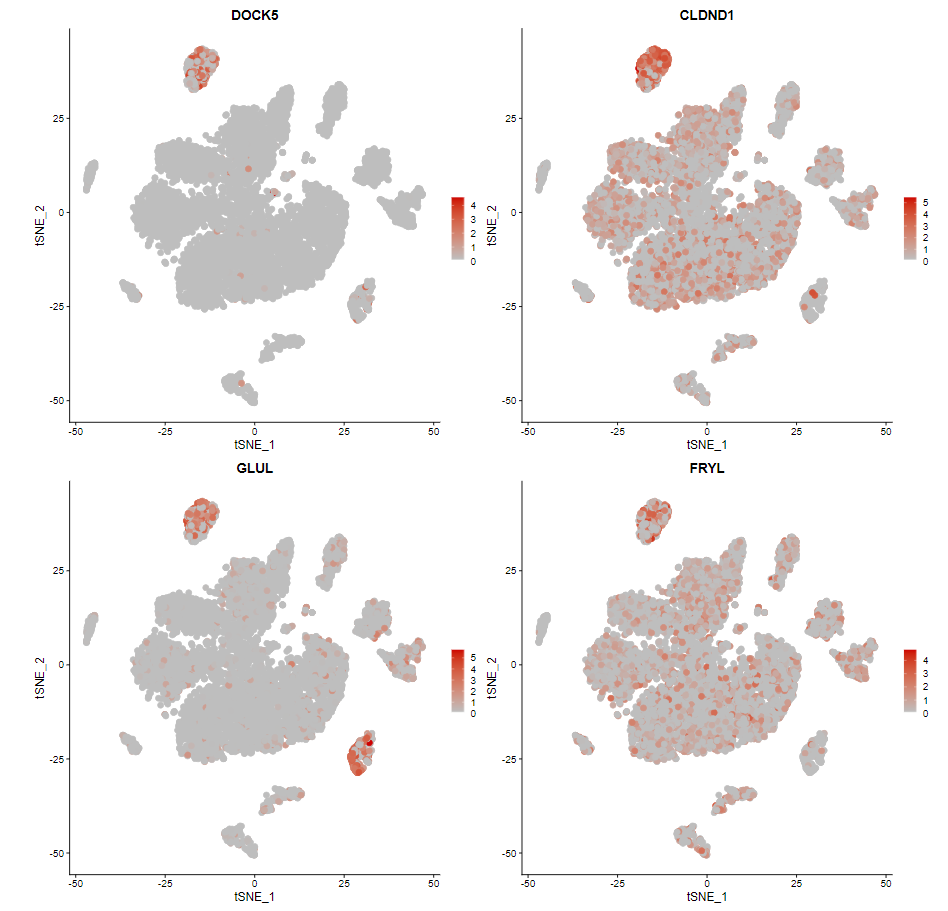


**Supplementary Figure. 21| Expression of top non-neuronal bias genes across clusters in ACC, MTG and VISP.** Feature plot of *DOCK5*, *CLDND1, GLUL* and *FRYL*.


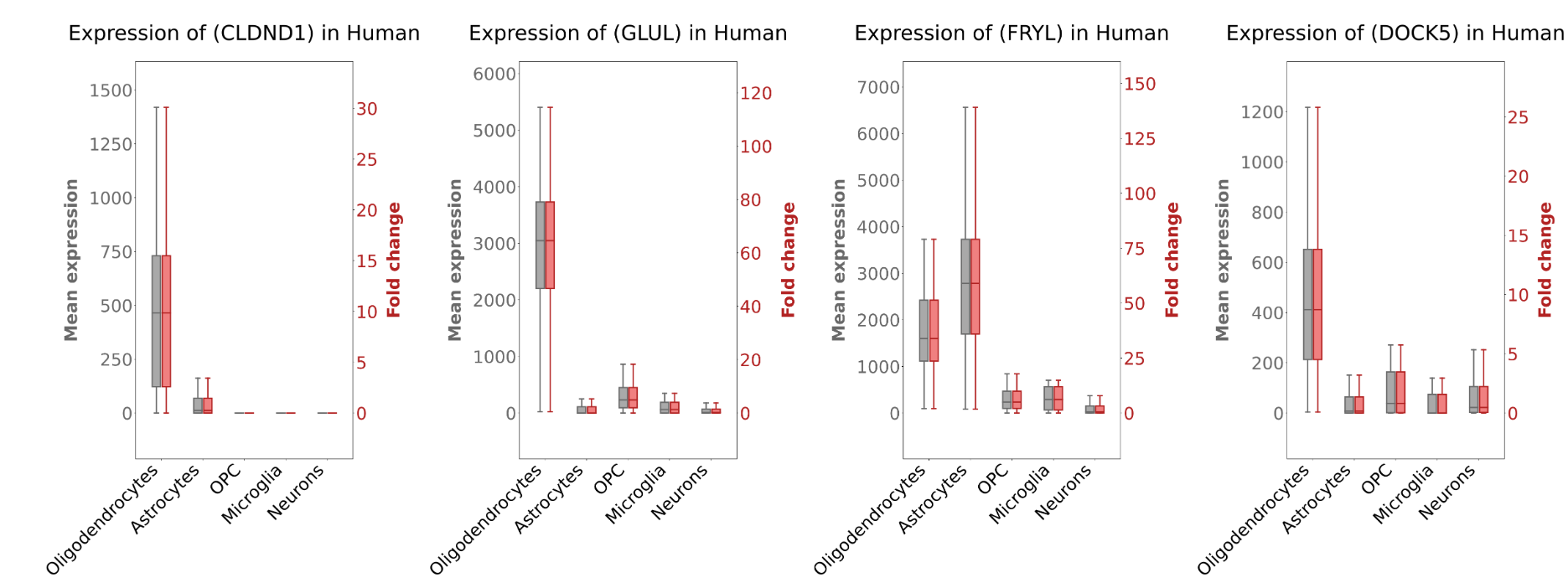


**Supplementary Figure. 22| Mean expression and fold change of non-neuronal bias genes across cell type in human (GSE67835).** Fold change (red) calculated wrt neurons across oligodendrocytes, astrocytes, OPC, microglia and neurons.


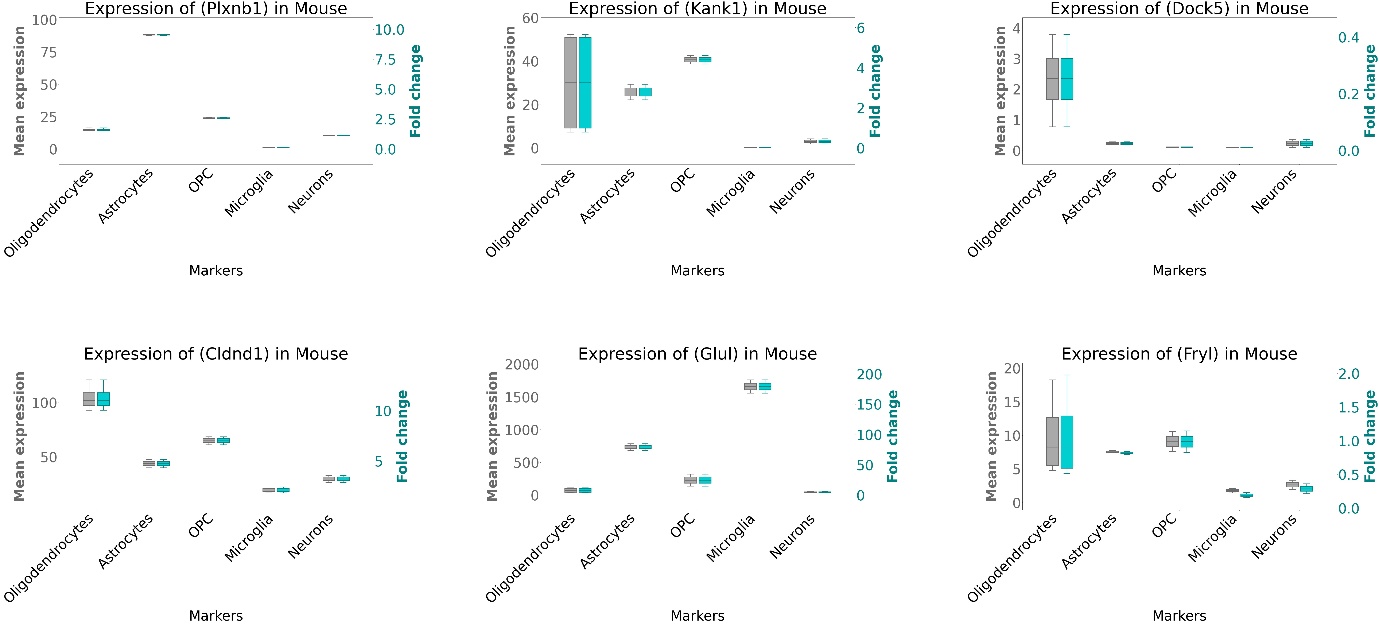


**Supplementary Figure. 23| Mean expression and fold change of non-neuronal bias genes across cell type in mouse (GSE52564).** Fold change (blue) calculated wrt neurons across oligodendrocytes, astrocytes, OPC, microglia and neurons.


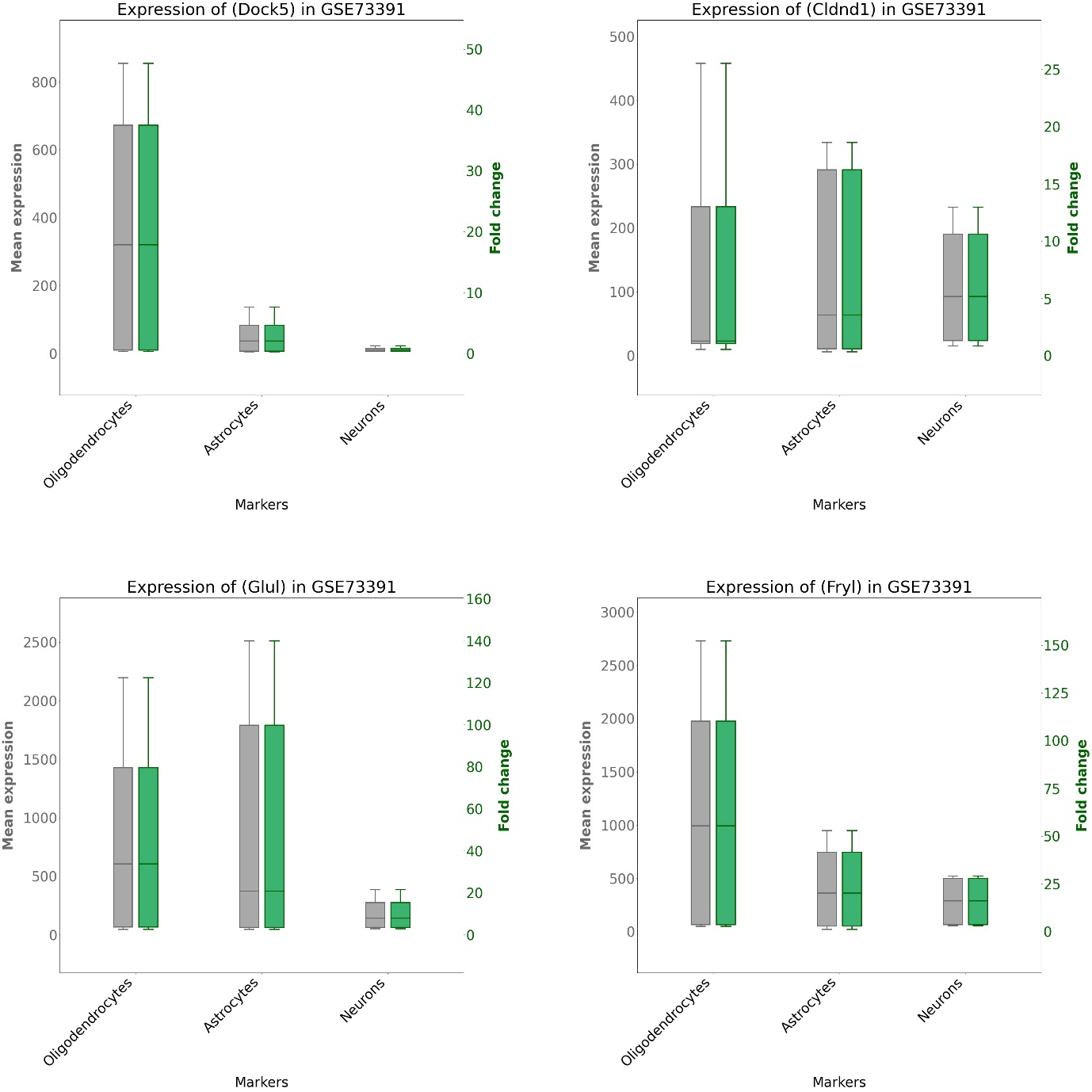


**Supplementary Figure. 24| Mean expression and fold change of non-neuronal bias genes across cell type in mouse (GSE73391).** Fold change (green) calculated wrt neurons across oligodendrocytes, astrocytes, and neurons.


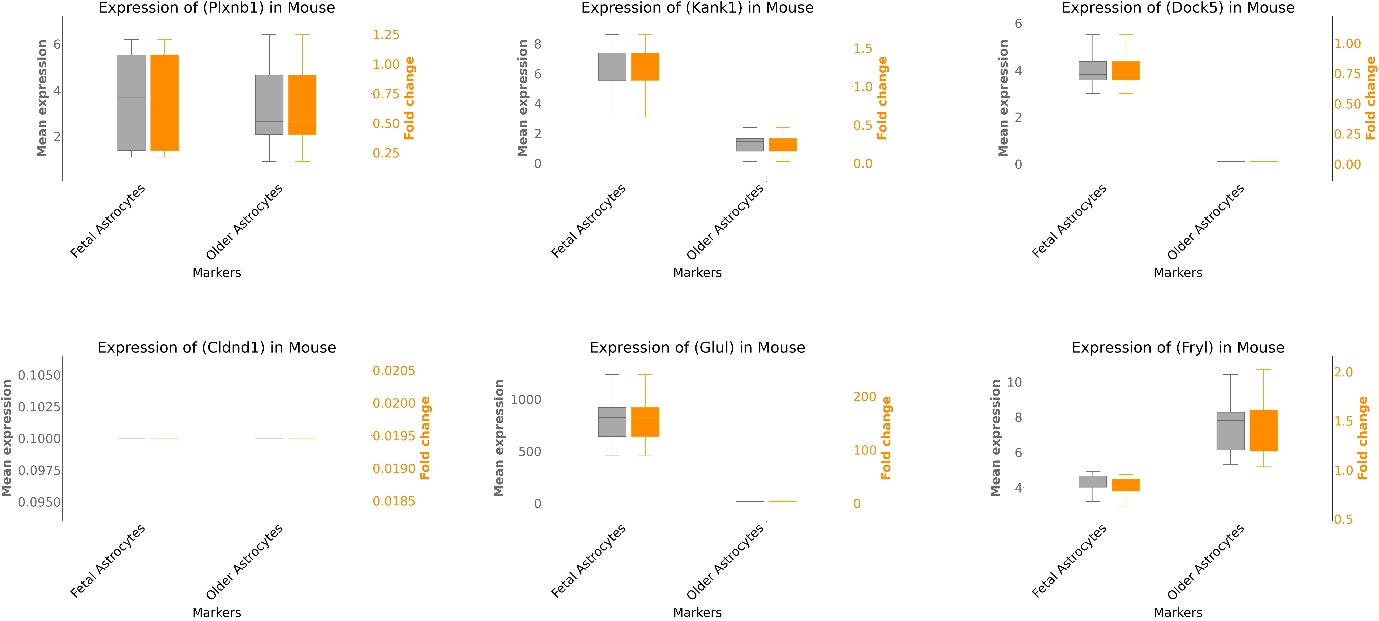


**Supplementary Figure. 25| Mean expression and fold change of non-neuronal bias genes across cell type in mouse (GSE73721).** Fold change (orange) calculated wrt adult astrocytes across fetal and older astrocytes.


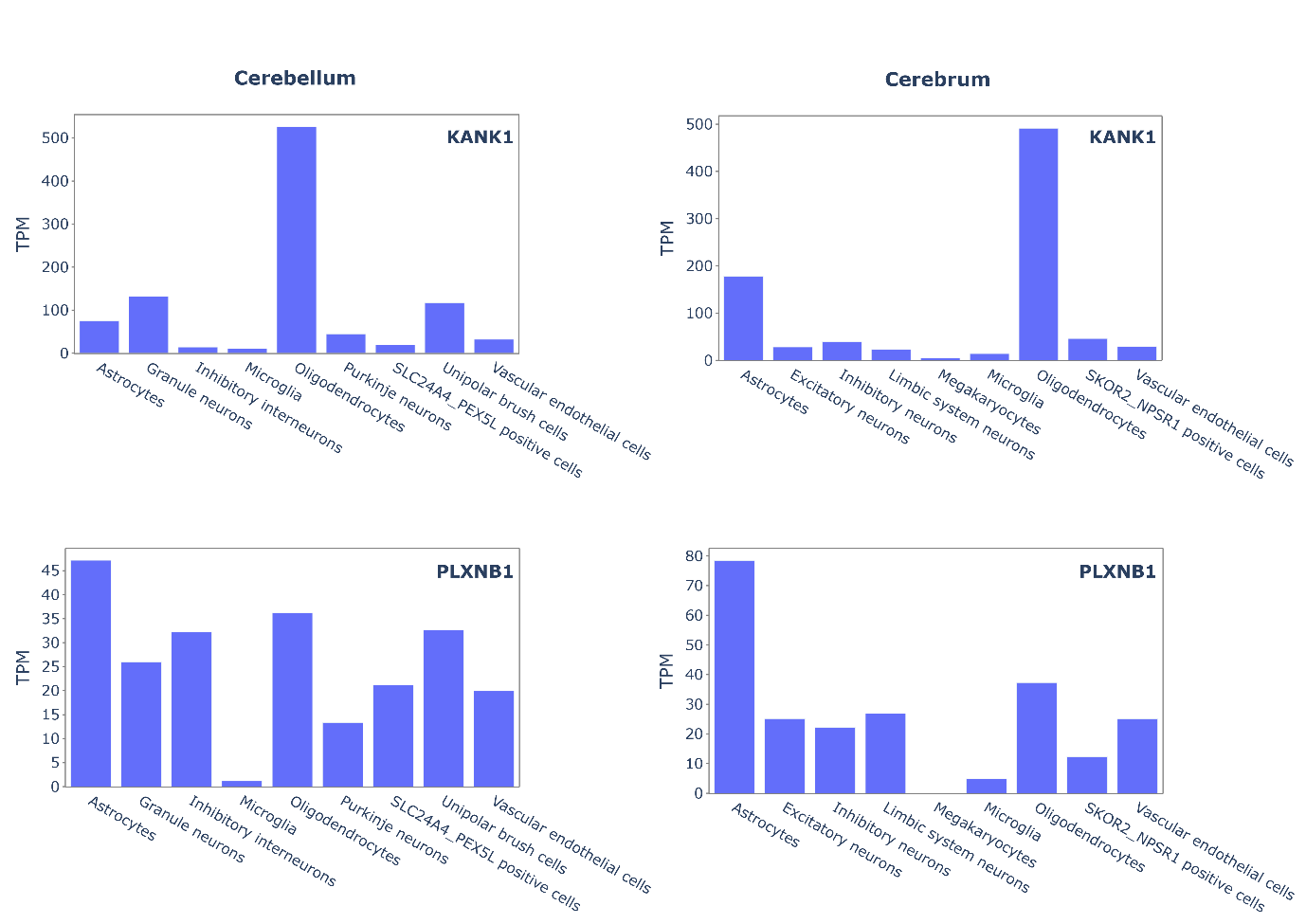


**Supplementary Figure. 26|** Expression (TPM) of *KANK1* and *PLXNB1* across cell types of cerebrum and cerebellum regions of fetal brain.


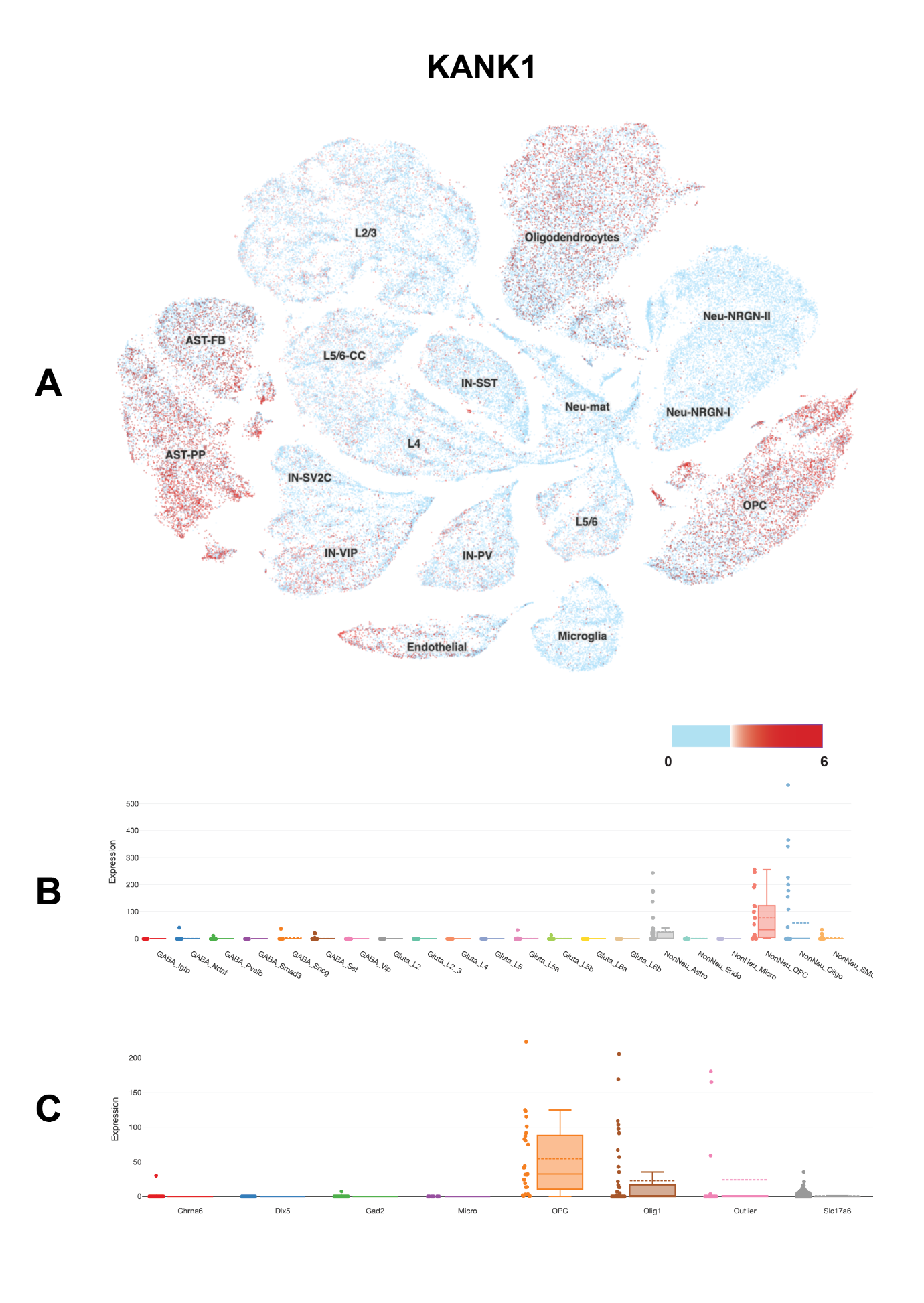

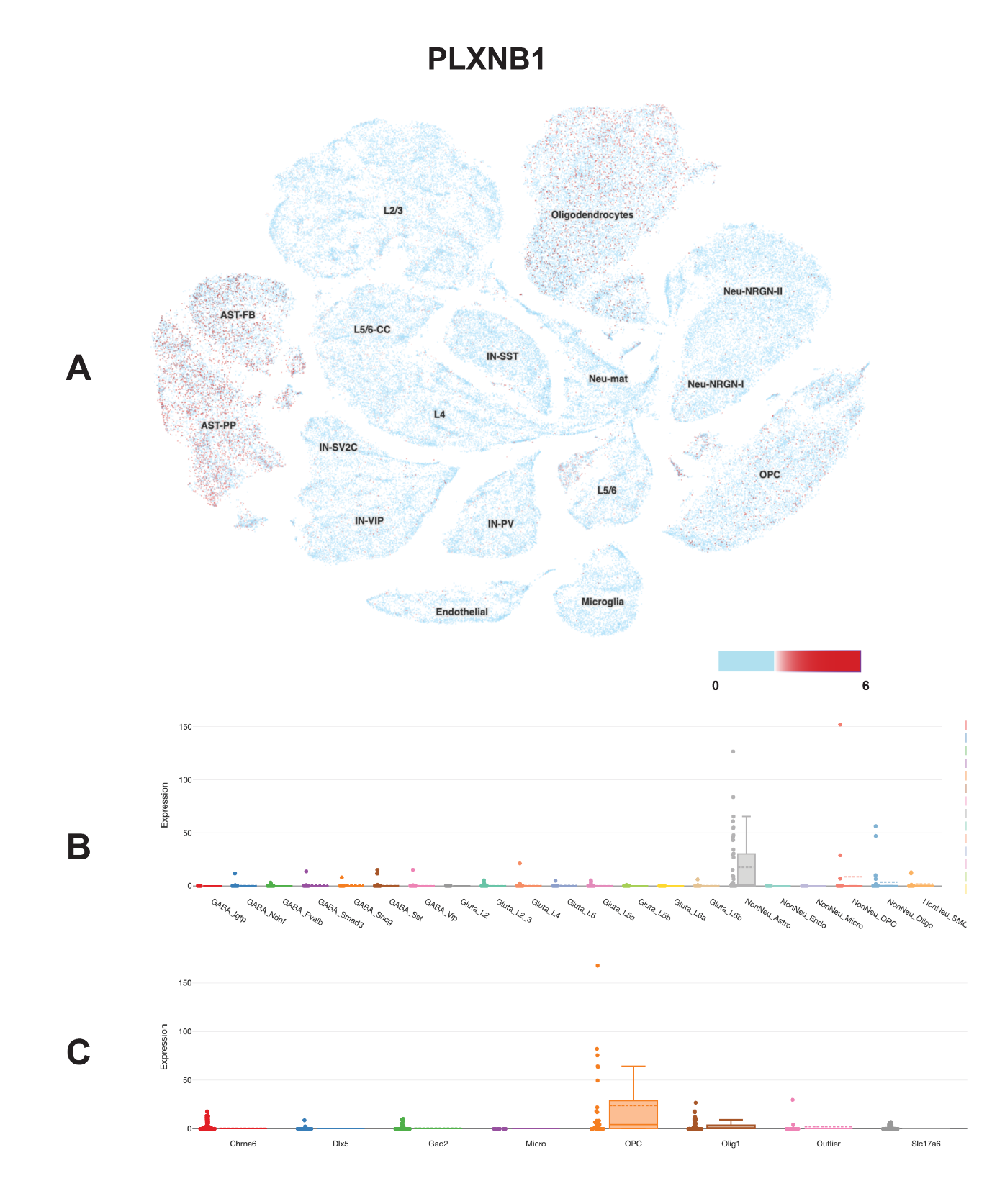


**Supplementary Figure. 27| Expression of *KANK1* and *PLXNB1* across brain cell types.** A) Feature plot of expression of *KANK1* and *PLXNB1* from UCSC single cell browser. B) Boxplot showing expression of *KANK1* and *PLXNB1* in adult mouse visual cortex single cell data. C) Boxplot showing expression of *KANK1* and *PLXNB1* in adult mouse lateral geniculate complex single cell data.

**Supplementary Tables**

Supplementary Table 1: Summary of mutation data collected from literature. Data extraction employed to collect key information from all 29 articles from the literature search.

Supplementary Table 2: ASD missense, LOF mutation genes.

Supplementary Table 3: Pathway enrichment of ASD LOF genes.

Supplementary Table 4: List of differentially expressed genes overlapped in 4 tests across clusters, top 20 DE, top 10 fold change genes in non-neuronal from 3 significant clusters across brain region.

Supplementary Table 5: Enrichment of cluster genes with brain critical exons across different developmental stages.

Supplementary Table 6: Enrichment of cluster genes with high pLI genes.

Supplementary Table 7: Enrichment of LOF genes across developmental time (early/ late).

Supplementary Table 8: Enrichment of ASD missense and LOF mutation genes across clusters.

Supplementary Table 9: Enrichment of ASD exonic, denovo and multiple LOF mutation genes.

Supplementary Table 10: List of fmRI, epilepsy and ID LOF genes, pathway genes related to ASD, housekeeping genes.

Supplementary Table 11: Enrichment of fmRI, epilepsy and ID LOF mutation genes across clusters.

Supplementary Table 12: Enrichment of pathway genes related to ASD across cluster genes.

Supplementary Table 13: List of known marker genes.

Supplementary Table 14: Composition of LOF genes (neuronal/ non neuronal).

Supplementary Table 15: Enrichment of critical exons across spatiotemporal brain landscape.

Supplementary Table 16: Pathway enrichment of cluster genes.

Supplementary Table 17: List of *KANK1* variants from cohorts, SNVs and CNVs from databases.

Supplementary Table 18: List of *PLXNB1* variants from cohorts, SNVs and CNVs from databases.

Supplementary Table 19: Association of genes and mutation types.

**References:**

1. Zhou, W., et al., *TransVar: a multilevel variant annotator for precision genomics.* Nat Methods, 2015. **12**(11): p. 1002-3.

2. Al-Mubarak, B., et al., *Whole exome sequencing reveals inherited and de novo variants in autism spectrum disorder: a trio study from Saudi families.* Sci Rep, 2017. **7**(1): p. 5679.

3. Wang, K., M. Li, and H. Hakonarson, *ANNOVAR: functional annotation of genetic variants from high-throughput sequencing data.* Nucleic Acids Res, 2010. **38**(16): p. e164.

4. Stuart, T., et al., *Comprehensive Integration of Single-Cell Data.* Cell, 2019. **177**(7): p. 1888-1902 e21.

5. Lek, M., et al., *Analysis of protein-coding genetic variation in 60,706 humans.* Nature, 2016. **536**(7616): p. 285-91.

6. Weddington, N., et al., *ReplicationDomain: a visualization tool and comparative database for genome-wide replication timing data.* BMC Bioinformatics, 2008. **9**: p. 530.
